# Patient-Centered Communication in Telehealth Settings

**DOI:** 10.1001/jamanetworkopen.2025.56291

**Published:** 2026-01-29

**Authors:** Lydia Tesfaye, Zahra Ansari, Michael Curry, Dennis Buckman, Eliseo J. Pérez-Stable, Sherine El-Toukhy

**Affiliations:** 1 Division of Intramural Research, National Institute on Minority Health and Health Disparities, National Institutes of Health, Bethesda, Maryland; 2 Now with School of Medicine, Emory University, Atlanta, Georgia; 3 Dell Medical School, University of Texas, Austin, Texas; 4 Information Management Services, Calverton, Maryland

## Abstract

**Importance:**

Patient-centered communication (PCC) is key to high-quality health care but remains understudied in telehealth visits.

**Objectives:**

To examine associations between individual-level characteristics and optimal levels of 7 PCC items and to assess whether these associations differ by county-level vulnerability to public health emergencies per the Minority Health Social Vulnerability Index (MHSVI).

**Design, Setting, and Participants:**

This was a cross-sectional, online survey of a nonprobability sample of adults, 18 years of age or older, who resided in MHSVI least- or most-vulnerable US counties. Data were collected between February 23 and August 26, 2022, and analyzed between January 10 and May 5, 2025.

**Exposures:**

Participant characteristics and county-level vulnerability.

**Main Outcomes and Measures:**

Self-reported optimal levels of 7 PCC items. For each item, multivariable logistic regression models were fit for the overall sample and stratified by MHSVI.

**Results:**

The survey participation rate was 17.0%. A total of 2754 adults (mean [SE] age, 43.9 [0.3] years) had at least 1 telehealth visit in the past year and resided in 649 of the MHSVI least- or most-vulnerable counties. The sample included 1568 females (56.9%), with 1650 participants (59.9%) self-identifying as White and 1104 participants (40.1%) self-identifying as being in a racial or ethnic minority group (Black or African American, 465 [16.9%]; Hispanic or Latino, 501 [18.2%]; and other, 138 [5.0%]). Optimal PCC ranged from 1069 participants (38.8%) self-reporting that clinicians spent sufficient time with them to 1372 participants (49.8%) reporting that clinicians ensured their understanding. Decreased odds of reporting optimal PCC (eg, clinicians ensuring understanding) were associated with educational attainment (adjusted odds ratio [AOR], 0.86 [95% CI, 0.77-0.96]) and not being proficient in English (AOR, 0.39 [95% CI, 0.28-0.53]). Increased odds were associated with digital health literacy domains (eg, access to digital services that work AOR, 1.49 [95% CI, 1.09-2.04]). Hispanic or Latino and Black or African American participants reported optimal PCC that was largely comparable with White participants. Associations between individual-level characteristics and optimal PCC differed by MHSVI. For example, Black or African American participants were less likely to report optimal time spent with clinicians in the MHSVI most-vulnerable counties (AOR, 0.73 [95% CI, 0.54-0.98]), but more likely to report that clinicians explained things in least-vulnerable counties (AOR, 1.74 [95% CI, 1.03-2.94]). Educational attainment was associated with lower odds of reporting optimal PCC in the MHSVI most-vulnerable counties only (eg, clinicians ensured understanding AOR, 0.85 [95% CI, 0.74-0.98]), whereas associations with English nonproficiency (eg, clinicians ensured understanding AOR, 0.40 [95% CI, 0.27-0.59] for most vulnerable and 0.35 [95% CI, 0.20-0.59] for least vulnerable) and digital health literacy (eg, clinicians gave the opportunity to ask questions for access to digital services that work AOR, 2.29 [95% CI, 1.50-3.49] for most vulnerable and 2.09 [95% CI, 1.31-3.36] for least vulnerable) were largely consistent across both MHSVI strata.

**Conclusions and Relevance:**

In this cross-sectional study, optimal PCC was comparable by race and ethnicity but not by educational attainment or English proficiency. Overall, the findings suggest that telehealth may facilitate optimal PCC.

## Key Points

**Question:**

Are individual-level characteristics associated with optimal patient-centered communication (PCC) in telehealth visits and do these associations differ by county-level factors per the Minority Health Social Vulnerability Index (MHSVI)?

**Findings:**

In this cross-sectional study of 2754 US adults, approximately 40% to 50% self-reported optimal levels of 7 PCC items, which were largely comparable by race and ethnicity and MHSVI strata, whereas English nonproficiency and educational attainment were associated with lower odds of optimal PCC. When stratified by MHSVI, associations were evident across both strata for English nonproficiency but only in the most vulnerable counties for educational attainment.

**Meaning:**

Findings indicate that telehealth may facilitate optimal PCC.

## Teaser

This cross-sectional study assesses whether individual-level characteristics are associated with optimal patient-centered communication in telehealth visits and whether any associations differ by county-level factors stratified by the Minority Health Social Vulnerability Index among US adults.

## Introduction

The rise in telehealth use necessitates an understanding of patient-centered communication (PCC) in virtual settings. PCC, in which clinicians understand and address patients’ needs and values, employ shared decision-making, and display empathy, is key to quality health care.^1,2,3^ PCC can favorably affect health by enhancing patient trust and medication adherence.^3,4,5,6^ Emphasis on PCC reflects a shift toward a patient-oriented model of medicine that affords patients greater autonomy and involvement in their health care.^7,8^

Telehealth, the delivery of health care using digital technologies, can improve health care access and quality by addressing issues such as health care costs, clinician shortages, and aging populations.^9^ Telehealth bypasses some access barriers to in-person care (eg, travel time).^10,11^ There is some evidence that telehealth can cost-effectively improve health outcomes, often producing results comparable with in-person care.^12,13^ Patients largely prefer in-person medical encounters^14,15^ vs telehealth visits^16^ but are willing to try telehealth visits.^14,17,18^

Although telehealth use has decreased since its peak in 2020, it remains higher than pre-2020 levels.^19,20^ Most PCC studies focus on in-person medical encounters or specialized populations (eg, patients with cancer).^21,22^ However, less is known about PCC in telehealth settings. Some studies of PCC in telehealth settings show that patients’ perceptions of PCC were equivalent to or better than in-person encounters,^16,23,24^ while other studies show dissatisfaction especially among selected populations (eg, older patients) and clinicians who interact with them.^25,26,27^ Evidence is scarce on the associations between macro-level factors and telehealth-based PCC, despite macro-level factors being important determinants of PCC within in-person clinical encounters^28,29^ and of health care access and outcomes.^30,31,32,33,34^ In addition, measures of PCC in telehealth settings are often qualitative or unstructured.^26,27,35^ When structured measures are used, aggregate results are often reported without differentiation between individual PCC functions.^16,24,25,36^ In this study, we used a validated scale^2,37^ to identify individual-level characteristics associated with optimal levels of 7 distinct PCC items in telehealth visits and to examine whether these associations differed by county-level vulnerability.

## Methods

The National Institutes of Health Institutional Review Board exempted this cross-sectional online survey study from review because the study met the federal criteria for exemption category §45 CFR 46.104(d)(2). We adhered to the Strengthening the Reporting of Observational Studies in Epidemiology (STROBE) reporting guideline.^38^ All participants e-consented to participate in the study and received monetary compensation.

### Study Sample

A nonprobability sample of 5559 US adults completed an online survey focused on understanding the disparities in digital technologies access and use between February 23, and August 26, 2022. Qualtrics email invited their survey panels who were at least 18 years of age and resided in 1571 counties that were in the top or bottom quartiles of the 2018 Minority Health Social Vulnerability Index (MHSVI), an index of county-level vulnerability to public health emergencies.^39^ Participants who did not reside in these counties were not allowed to complete the survey.

The MHSVI is composed of 34 indicators grouped into 6 themes: (1) socioeconomic status (eg, persons living below the federal poverty line), (2) household composition and disability (eg, population with a disability), (3) minority status and language (eg, Spanish speakers who speak English less than very well), (4) housing type and transportation (eg, households with no vehicle available), (5) health care infrastructure and access (eg, persons without health insurance), and (6) medical vulnerability (eg, cardiovascular disease mortality per 100 000, persons without internet access).^39^ Indicators were derived from various data sources (eg, American Community Survey, Centers for Disease Control and Prevention Interactive Atlas of Heart Disease and Stroke).^39^ To enable each theme to contribute equally to the MHSVI score, we summed the percentile ranking of each theme and scaled the sums to values between 0 and 1, with 1 representing greater vulnerability.

We used data from the National Center for Health Statistics to calculate sample targets that reflected the age, sex, and race and ethnicity characteristics of residents in the most and least vulnerable counties of the MHSVI.^40^ We excluded participants who had inconsistent responses to 2 age questions (n = 115) as a data quality measure. We then limited the analytic sample to participants who reported at least 1 telehealth visit in the past year. Description of study methodology appears in other publications based on this project.^41^

### Measures

We used a validated 7-item scale that captured core functions of patient-clinician communication.^2,37^ The root question was, “The following questions are about your remote video or audio communication with all doctors, nurses, or other health professionals you saw during the past 12 months. How often did they do each of the following:” (1) “Give you the chance to ask all the health-related questions you had,” (2) “Give the attention you needed to your feelings and emotions,” (3) “Involve you in decisions about your health care as much as you wanted,” (4) “Make sure you understood the things you needed to do to take care of your health,” (5) “Explain things in a way you could understand,” (6) “Spend enough time with you,” and (7) “Help you deal with feelings of uncertainty about your health or health care.” Response choices were always, usually, sometimes, and never. An a priori decision was made to dichotomize all items into always vs all other responses, with always reflecting optimal PCC.^22,42^

We collected data on age; biological sex; sexual orientation; self-identified race and ethnicity from a list that included Alaska Native or American Indian, Asian American, African American or Black, Hispanic, Latino, or Spanish origin (collapsed to Hispanic or Latino in analyses), Middle Eastern or North African (collapsed with White in analyses), Native Hawaiian or Pacific Islander, and White; educational attainment; 2021 income; English proficiency; general and mental health; having health insurance, a primary care clinician, or underlying medical conditions; prior COVID-19 infection; past-year in-person visits with a health care clinician; lifestyle factors, including physical activity, cigarette and e-cigarette use, marijuana use, alcohol misuse; self-reported body mass index (calculated as weight in kilograms divided by height in meters squared); device and internet access; and digital health literacy (DHL) using the eHealth Literacy Questionnaire.^43^ Cigarette smoking, e-cigarette use, and marijuana use indicated self-reported use on 1 or more days in the past month. Alcohol misuse was defined as 3 or more daily drinks and 2 or more daily drinks for men and women, respectively. Device access included audio- and video-enabled devices (ie, desktop or laptop computer at work or home, tablet computer, and smartphone) and audio-only devices (ie, basic cellphone, landline). Internet access included dial-up telephone line, broadband high-speed internet, satellite internet service, cellular data plan for mobile devices, and free wireless network. DHL was measured using 35 items on a 4-point Likert scale that made up 7 domains. Items under each domain, which ranged from 4 to 6 items, were averaged, with higher mean scores indicating higher DHL.

### Statistical Analysis

Imputing values for 6 variables with missing data, we generated 25 imputed datasets using the SAS MI procedure (SAS Institute Inc) with the fully conditional specification approach and missing-at-random assumption. Differences between participants with and without missing data appear in eTable 1 in Supplement 1. The imputation models incorporated all study variables, including outcome variables. Using the imputed datasets and multiple imputation capabilities in SUDAAN (RTI International), we fit multivariable logistic regression models for the response variables indicating optimal PCC and adjusting for covariates using the overall sample and MHSVI strata. Using the fitted models and predictive margins calculations implemented in SUDAAN, we estimated the predicted marginal probability of each optimal PCC response, adjusting for sociodemographic, health, and technology-related covariates.^44^ Covariates were selected a priori based on subject matter knowledge.

For each model, we report the adjusted odds ratios (AORs), predicted marginal (PM) probabilities, and 95% CIs. Age and DHL domains were treated as continuous variables, income and education as ordinal, and all others as categorical. Due to low frequencies, we grouped Alaska Native or American Indian (n = 32), Asian (n = 98), and Native Hawaiian or Pacific Islander (n = 8) into an other race category. We grouped access to audio-only devices (n = 13) with access to audio and video devices (n = 2700). For a sensitivity analysis, we conducted a complete case analysis, which required assuming data were missing completely at random, and compared the results to the multiple imputation analysis results. We used SAS,^45^ version 9.4, and SUDAAN,^46^ version 11.0.4, for all statistical analyses. Results were considered statistically significant when 95% CIs did not include the null value of 1.

## Results

The survey participation rate was 17.0%. The sample included 2754 participants who resided in 649 MHSVI most- and least-vulnerable counties (1568 female [56.9%], 1186 male [43.1%]), with a mean (SE) age of 43.9 (0.3) years and 889 with a high school education or less (32.3%). Participants self-identified as African American or Black (465 [16.9%]), Hispanic or Latino (501 [18.2%]), White (1650 [59.9%]), and other (138 [5.0%]), which included Alaska Native or American Indian, Asian, and Native Hawaiian or Pacific Islander (Table 1). Differences between participants who had vs did not have telehealth visits appear in eTable 1 in Supplement 1. Characteristics of participants who exclusively had past-year telehealth visits, in-person visits, and both telehealth and in-person visits vs no visits appear in eTable 2 in Supplement 1.

**Table 1.** Participant Sample Characteristics Overall and Stratified by MHSVI^a^

Characteristic	Participants, No. (%)			*P* value
	Overall sample (N = 2754)	MHSVI		
		Most-vulnerable counties (n = 1505)	Least-vulnerable counties (n = 1249)	
Age, mean (SE), y	43.9 (0.3)	41.7 (0.4)	46.6 (0.4)	<.001
18-29	668 (24.3)	402 (26.7)	266 (21.3)	<.001
30-44	807 (29.3)	482 (32.0)	325 (26.0)	
45-59	711 (25.8)	390 (25.9)	321 (25.7)	
≥60	568 (20.6)	231 (15.4)	337 (27.0)	
Sex				
Female	1568 (56.9)	896 (59.5)	672 (53.8)	.002
Male	1186 (43.1)	609 (40.5)	577 (46.2)	
Sexual orientation				
Heterosexual	2384 (86.6)	1306 (86.8)	1078 (86.3)	.71
Gay, lesbian, bisexual	370 (13.4)	199 (13.2)	171 (13.7)	
Race and ethnicity				
Alaska Native or American Indian	32 (1.2)	29 (1.9)	3 (0.2)	<.001
Asian or Native Hawaiian or Pacific Islander	106 (3.8)	44 (2.9)	62 (5.0)	
Black or African American	465 (16.9)	365 (24.3)	100 (8.0)	
Hispanic or Latino	501 (18.2)	388 (25.8)	113 (9.1)	
White	1650 (59.9)	679 (45.1)	971 (77.7)	
Educational attainment				
<High school	144 (5.2)	97 (6.4)	47 (3.8)	<.001
High school graduate	745 (27.1)	460 (30.6)	285 (22.8)	
Vocational school, some college	1015 (36.9)	572 (38.0)	443 (35.5)	
College graduate or higher	850 (30.9)	377 (25.0)	473 (37.9)	
Income, $				
<20 000	708 (25.7)	494 (32.8)	214 (17.1)	<.001
20 000 to 49 999	856 (31.1)	506 (33.6)	350 (28.0)	
50 000 to 74 999	478 (17.4)	227 (15.1)	251 (20.1)	
≥75 000	712 (25.9)	279 (18.5)	434 (34.7)	
English proficiency				
Very well	2466 (89.5)	1323 (87.9)	1143 (91.5)	.001
Well, not well, not at all	288 (10.5)	182 (12.1)	106 (8.5)	
Health insurance				
Insured	2539 (92.2)	1346 (89.4)	1193 (95.5)	<.001
Uninsured	215 (7.8)	159 (10.6)	56 (4.5)	
General health				
Excellent, very good, good	2011 (73.0)	1090 (72.4)	921 (73.7)	.43
Fair, poor	743 (27.0)	415 (27.6)	328 (26.3)	
Mental health				
Excellent, very good, good	1932 (70.2)	1048 (69.6)	884 (70.8)	.51
Fair, poor	822 (29.8)	457 (30.4)	365 (29.2)	
Has a primary care clinician				
Yes	1524 (55.3)	792 (52.6)	732 (58.6)	.001
No	1230 (44.7)	713 (47.4)	517 (41.4)	
Presence of underlying clinical conditions				
Yes	1058 (38.4)	516 (34.3)	542 (43.4)	<.001
No	1696 (61.6)	989 (65.7)	707 (56.6)	
History of COVID-19 infection				
Yes	843 (30.6)	466 (31.0)	377 (30.2)	.64
No	1911 (69.4)	1039 (69.0)	872 (69.8)	
No. of past-year in-person visits with health care clinician				
0	220 (8.0)	107 (7.1)	113 (9.1)	.06
≥1	2534 (92.0)	1398 (92.9)	1136 (90.9)	
Body mass index^b^				
Healthy (18.5 to <25)	776 (28.2)	406 (27.0)	370 (29.6)	.15
Unhealthy (<18.5 or ≥25)	1978 (71.8)	1099 (73.0)	879 (70.4)	
Physical activity				
Sufficient (≥150 min/wk)	1898 (68.9)	1077 (71.6)	821 (65.7)	.001
Insufficient (<150 min/wk)	856 (31.1)	428 (28.4)	428 (34.3)	
Past mo cigarette smoking				
Yes	1019 (37.0)	661 (43.9)	358 (28.7)	<.001
No	1735 (63.0)	844 (56.1)	891 (71.3)	
Past mo e-cigarette use				
Yes	674 (24.5)	422 (28.0)	252 (20.2)	<.001
No	2080 (75.5)	1083 (72.0)	997 (79.8)	
Alcohol misuse^c^				
Yes	185 (6.7)	97 (6.5)	88 (7.1)	.24
No	2430 (88.2)	1323 (87.9)	1107 (88.6)	
Not applicable (<21 y of age)	139 (5.1)	85 (5.7)	54 (4.3)	
Past mo marijuana use				
Yes	878 (31.9)	557 (37.0)	321 (25.7)	<.001
No	1876 (68.1)	948 (63.0)	928 (74.3)	
Device access				
Video and audio access	2700 (98.0)	1469 (97.6)	1231 (98.6)	.18
Audio-only access	13 (0.5)	9 (0.6)	4 (0.3)	
None	41 (1.5)	27 (1.8)	14 (1.1)	
Internet access				
Yes	2700 (98.0)	1470 (97.7)	1230 (98.5)	.12
No	54 (2.0)	35 (2.3)	19 (1.5)	
Digital health literacy, mean (SE)^d^				
Using technology to process health information	3.02 (0.01)	3.03 (0.01)	3.00 (0.01)	.12
Understanding of health concepts and language	3.05 (0.01)	3.06 (0.01)	3.05 (0.01)	.63
Ability to actively engage with digital services	3.02 (0.01)	3.04 (0.01)	3.01 (0.02)	.12
Feel safe and in control	2.90 (0.01)	2.93 (0.02)	2.87 (0.02)	.009
Motivated to engage with digital services	2.99 (0.01)	3.01 (0.01)	2.97 (0.01)	.03
Access to digital services that work	3.01 (0.01)	3.02 (0.01)	3.00 (0.01)	.39
Digital services that suit individual needs	2.92 (0.01)	2.95 (0.02)	2.87 (0.02)	<.001

Abbreviation: MHSVI, Minority Health Social Vulnerability Index.

^a^ Based on imputed data.

^b^ Body mass index calculated as weight in kilograms divided by height in meters squared.

^c^ Alcohol misuse was defined as 3 or more daily drinks and 2 or more daily drinks for men and women, respectively.

^d^ Measured using 35 items on a 4-point Likert scale that made up 7 domains, with 4 to 6 items in each domain; higher mean scores indicate greater digital health literacy.

### Factors Associated With PCC

Roughly half of 2754 participants (1372 [49.8%]) reported that clinicians always ensured patient understanding, and 38.8% (1069 participants) reported that clinicians always spent enough time with them (Table 2). Similar findings were observed across MHSVI most- and least-vulnerable counties.

**Table 2.** Distribution of Optimal vs Suboptimal PCC Among Participants Who Had 1 or More Telehealth Visits in the Past Year

PCC item^a^	Participants, No. (%)			*P* value
	Overall sample (N = 2754)	MHSVI		
		Most-vulnerable counties (n = 1505)	Least-vulnerable counties (n = 1249)	
PCC 1: Give you the chance to ask questions				
Always	1257 (45.6)	677 (45.0)	580 (46.4)	.44
All other	1497 (54.4)	828 (55.0)	669 (53.6)	
PCC 2: Give attention to your feelings				
Always	1151 (41.8)	639 (42.5)	512 (41.0)	.43
All other	1603 (58.2)	866 (57.5)	737 (59.0)	
PCC 3: Involve you in decisions				
Always	1236 (44.9)	665 (44.2)	571 (45.7)	.42
All other	1518 (55.1)	840 (55.8)	678 (54.3)	
PCC 4: Make sure you understand				
Always	1372 (49.8)	731 (48.6)	641 (51.3)	.15
All other	1382 (50.2)	774 (51.4)	608 (48.7)	
PCC 5: Explain things				
Always	1369 (49.7)	728 (48.4)	641 (51.3)	.12
All other	1385 (50.3)	777 (51.6)	608 (48.7)	
PCC 6: Spend enough time				
Always	1069 (38.8)	565 (37.5)	504 (40.4)	.13
All other	1685 (61.2)	940 (62.5)	745 (59.6)	
PCC 7: Help deal with uncertainty				
Always	1096 (39.8)	609 (40.5)	487 (39.0)	.43
All other	1658 (60.2)	896 (59.5)	762 (61.0)	

Abbreviations: MHSVI, Minority Health Social Vulnerability Index; PCC, patient-centered communication.

^a^ Always reflects optimal PCC, whereas all other includes response choices usually, sometimes, and never, reflecting suboptimal PCC.

Overall, race and ethnicity, educational attainment, and English proficiency were associated with most PCC items (Table 3; eFigure in Supplement 1). Hispanic or Latino and Black or African American participants reported optimal PCC comparable with White participants on 7 and 6 items, respectively. Compared with White participants, Black or African American participants were less likely to report that clinicians always spent enough time with them (AOR, 0.74 [95% CI, 0.57-0.95]; PM, 40.8% vs 34.7%). Conversely, compared with White participants, Alaska Native or American Indian, Asian, and Native Hawaiian or Pacific Islander (collapsed as other race) were less likely to report that clinicians always addressed their feelings (AOR, 0.50 [95% CI, 0.34-0.75]; PM, 43.3% vs 29.7%), involved them in decision-making (AOR, 0.52 [95% CI, 0.35-0.77]; PM, 45.6% vs 32.2%), explained things (AOR, 0.62 [95% CI, 0.42-0.90]; PM, 50.1% vs 40.1%), spent ample time with them (AOR, 0.58 [95% CI, 0.39-0.88]; PM, 40.8% vs 30.2%), or managed uncertainties (AOR, 0.50 [95% CI, 0.33-0.74]; PM, 40.4% vs 26.9%). Greater educational attainment was associated with lower odds of reporting optimal levels of 4 PCC items (eg, clinician ensuring understanding AOR, 0.86 [95% CI, 0.77-0.96]), with 55.7% of participants with less than a high school degree reporting that clinicians always involved them in decision-making vs 46.6% of participants with a college degree or higher. Participants with limited English proficiency (vs proficient individuals) had lower odds of reporting optimal PCC across all 7 items (eg, clinician ensuring understanding AOR, 0.39 [95% CI, 0.28-0.53]; PM, 31.8% vs 51.7%).

**Table 3.** Factors Associated With Optimal PCC Among 2754 Participants Who Had 1 or More Telehealth Visits in the Past Year^a^

Characteristic	AOR (95% CI)^b^						
	PCC 1^c^	PCC 2^c^	PCC 3^c^	PCC 4^c^	PCC 5^c^	PCC 6^c^	PCC 7^c^
Age	1.00 (0.99-1.01)	1.00 (0.99-1.01)	1.00 (0.99-1.01)	1.00 (1.00-1.01)	1.00 (1.00-1.01)	1.01 (1.00-1.01)	1.00 (0.99-1.01)
Sex							
Female	1.17 (0.98-1.40)	1.34 (1.12-1.60)	1.24 (1.04-1.47)	1.18 (0.99-1.40)	1.26 (1.05-1.50)	1.26 (1.06-1.51)	1.15 (0.96-1.37)
Male	1 [Reference]	1 [Reference]	1 [Reference]	1 [Reference]	1 [Reference]	1 [Reference]	1 [Reference]
Race and ethnicity							
Alaska Native or American Indian, Asian, or Native Hawaiian or Pacific Islander	0.69 (0.48-1.01)	0.50 (0.34-0.75)	0.52 (0.35-0.77)	0.81 (0.56-1.19)	0.62 (0.42-0.90)	0.58 (0.39-0.88)	0.50 (0.33-0.74)
Black or African American	0.85 (0.66-1.08)	0.90 (0.70-1.15)	0.95 (0.75-1.22)	1.02 (0.80-1.30)	1.11 (0.87-1.42)	0.74 (0.57-0.95)	0.95 (0.74-1.22)
Hispanic or Latino	1.08 (0.85-1.38)	0.87 (0.68-1.11)	1.00 (0.79-1.28)	1.05 (0.82-1.34)	0.91 (0.71-1.17)	0.89 (0.69-1.14)	1.04 (0.82-1.33)
White	1 [Reference]	1 [Reference]	1 [Reference]	1 [Reference]	1 [Reference]	1 [Reference]	1 [Reference]
Educational attainment	0.92 (0.83-1.03)	0.93 (0.84-1.04)	0.85 (0.76-0.95)	0.86 (0.77-0.96)	0.91 (0.82-1.01)	0.87 (0.77-0.97)	0.86 (0.77-0.96)
Income	0.96 (0.88-1.05)	0.97 (0.89-1.06)	0.99 (0.91-1.08)	0.97 (0.89-1.06)	1.00 (0.92-1.09)	0.98 (0.89-1.07)	0.93 (0.85-1.01)
English proficiency							
Very well	1 [Reference]	1 [Reference]	1 [Reference]	1 [Reference]	1 [Reference]	1 [Reference]	1 [Reference]
Well, not well, not at all	0.43 (0.31-0.58)	0.66 (0.48-0.89)	0.47 (0.34-0.64)	0.39 (0.28-0.53)	0.48 (0.35-0.66)	0.56 (0.40-0.77)	0.46 (0.33-0.63)
Has a primary care clinician							
Yes	1 [Reference]	1 [Reference]	1 [Reference]	1 [Reference]	1 [Reference]	1 [Reference]	1 [Reference]
No	0.59 (0.50-0.71)	0.63 (0.53-0.76)	0.59 (0.50-0.71)	0.56 (0.47-0.67)	0.65 (0.54-0.77)	0.65 (0.54-0.78)	0.68 (0.57-0.82)
Digital health literacy							
Using technology to process health information	0.89 (0.65-1.21)	0.76 (0.55-1.05)	0.70 (0.51-0.95)	0.67 (0.49-0.90)	0.62 (0.45-0.85)	0.75 (0.54-1.04)	0.66 (0.48-0.90)
Understanding of health concepts and language	1.28 (0.95-1.71)	1.37 (1.01-1.85)	1.19 (0.88-1.60)	1.52 (1.13-2.05)	2.02 (1.49-2.74)	1.32 (0.97-1.80)	1.48 (1.09-2.01)
Ability to actively engage with digital services	0.92 (0.72-1.20)	0.86 (0.66-1.12)	1.42 (1.09-1.84)	1.04 (0.80-1.34)	1.22 (0.94-1.59)	0.86 (0.65-1.13)	0.81 (0.62-1.06)
Feel safe and in control	1.12 (0.91-1.37)	1.49 (1.20-1.83)	1.36 (1.11-1.66)	1.34 (1.10-1.64)	1.21 (0.99-1.48)	1.60 (1.28-1.99)	1.45 (1.17-1.80)
Motivated to engage with digital services	1.02 (0.74-1.41)	1.28 (0.92-1.77)	1.14 (0.83-1.58)	1.13 (0.83-1.53)	1.15 (0.83-1.60)	1.08 (0.77-1.51)	1.35 (0.97-1.88)
Access to digital services that work	2.16 (1.58-2.96)	1.62 (1.17-2.25)	1.52 (1.10-2.09)	1.49 (1.09-2.04)	1.45 (1.06-1.99)	1.55 (1.11-2.16)	1.64 (1.17-2.31)
Digital services that suit individual needs	1.23 (0.95-1.59)	1.43 (1.10-1.87)	1.23 (0.95-1.61)	1.37 (1.06-1.77)	1.16 (0.89-1.50)	1.58 (1.21-2.07)	1.36 (1.04-1.78)
MHSVI							
Least-vulnerable counties	1 [Reference]	1 [Reference]	1 [Reference]	1 [Reference]	1 [Reference]	1 [Reference]	1 [Reference]
Most-vulnerable counties	1.01 (0.84-1.20)	1.11 (0.93-1.33)	0.99 (0.82-1.18)	0.93 (0.77-1.11)	0.96 (0.81-1.15)	0.94 (0.78-1.13)	1.04 (0.87-1.25)

Abbreviations: AOR, adjusted odds ratio; MHSVI, Minority Health Social Vulnerability Index; PCC, patient-centered communication.

^a^ Based on imputed data.

^b^ Logistic regression analysis modeled the probability of 1 equal to always. Models adjusted for sexual orientation, health insurance, general and mental health, presence of underlying clinical conditions, history of COVID-19 infection, past-year in-person visit with health care clinician, lifestyle factors (ie, body mass index; physical activity; past-month cigarette smoking, e-cigarette use, marijuana or cannabis use; alcohol misuse), device and internet access.

^c^ PCC 1, gives you the chance to ask questions; PCC 2, gives attention to your feelings; PCC 3, involves you in decisions; PCC 4, makes sure you understand; PCC 5, explains things; PCC 6, spends enough time; and PCC 7, helps deal with uncertainty.

Higher scores on 5 DHL domains of (1) having access to digital services that work (eg, clinicians ensuring understanding AOR, 1.49 [95% CI, 1.09-2.04]), (2) feeling safe and in control (eg, clinicians managed uncertainties AOR, 1.45 [95% CI, 1.17-1.80]), (3) understanding of health concepts and language (eg, clinicians explained things AOR, 2.02 [95% CI, 1.49-2.74]), (4) having digital services that suit individual needs (eg, clinicians spent enough time AOR, 1.58 [95% CI, 1.21-2.07]), and (5) having the ability to actively engage with digital services (eg, clinicians involved them in decisions AOR, 1.42 [95% CI, 1.09-1.84]) were associated with higher odds of reporting optimal PCC. Conversely, higher scores on the DHL domain “using technology to process health information” was associated with lower odds of reporting optimal involvement in decision-making (AOR, 0.70 [95% CI, 0.51-0.95]), ensuring understanding (AOR, 0.67 [95% CI, 0.49-0.90]), explaining things (AOR, 0.62 [95% CI, 0.45-0.85]), and managing uncertainty (AOR, 0.66 [95% CI, 0.48-0.90]). The DHL domain, “motivated to engage with digital services” was not associated with optimal reporting of any PCC items.

### Factors Associated With PCC Stratified by MHSVI

MHSVI strata were not associated with any PCC item (Table 3). Of MHSVI individual themes, only county-level socioeconomic status was associated with lower odds of participants reporting that clinicians always explained things (AOR, 0.49 [95% CI, 0.25-0.95]) (eTable 3 in Supplement 1).

Some associations between individual-level characteristics and optimal PCC varied by MHSVI. In most vulnerable counties, educational attainment was associated with lower odds of reporting that clinicians gave chances to ask questions (AOR, 0.86 [95% CI, 0.75-0.99]), involved participants in decision-making (AOR, 0.83 [95% CI, 0.73-0.96]), ensured their understanding (AOR, 0.85 [95% CI, 0.74-0.98]), spent ample time (AOR, 0.85 [95% CI, 0.74-0.99]), and managed uncertainty (AOR, 0.84 [95% CI, 0.73-0.96] (Table 4). No associations were observed between educational attainment and PCC in least vulnerable counties (Table 5). Compared with White participants, Black or African American participants had higher odds of reporting that clinicians always explained things in least-vulnerable counties (AOR, 1.74 [95% CI, 1.03-2.94]) but had lower odds of reporting that clinicians always spent ample time with them in most-vulnerable counties (AOR, 0.73 [95% CI, 0.54-0.98]). In least-vulnerable counties, Alaska Native or American Indian, Asian, and Native Hawaiian or Pacific Islander (collapsed as other race) had lower odds of reporting optimal levels of 6 PCC items (eg, clinicians always gave chances to ask questions AOR, 0.51 [95% CI, 0.28-0.92]). They also had lower odds of reporting that clinicians always addressed their feelings (AOR, 0.59 [95% CI, 0.35-0.99]) and involved them in decision-making (AOR, 0.56 [95% CI, 0.33-0.93]) in most vulnerable counties. Sensitivity analyses showed that Asian participants were the primary drivers of the observed associations between the other race group and PCC (eTables 4 through 6 in Supplement 1). Other associations similar across MHSVI strata included English nonproficiency (eg, clinicians ensured understanding AOR, 0.40 [95% CI, 0.27-0.59] for most vulnerable and 0.35 [95% CI, 0.20-0.59] for least vulnerable) and digital health literacy (eg, clinicians gave the opportunity to ask questions for access to digital services that work AOR, 2.29 [95% CI, 1.50-3.49] for most vulnerable and 2.09 [95% CI, 1.31-3.36] for least vulnerable).

**Table 4.** Factors Associated With Optimal PCC Among 1505 Participants Who Had 1 or More Telehealth Visits in the Past Year and Resided in the MHSVI Most-Vulnerable Counties^a^

Characteristics	AOR (95% CI)^b^						
	PCC 1^c^	PCC 2^c^	PCC 3^c^	PCC 4^c^	PCC 5^c^	PCC 6^c^	PCC 7^c^
Age	1.00 (0.99-1.01)	1.00 (0.99-1.00)	1.00 (0.99-1.01)	0.99 (0.99-1.00)	1.00 (0.99-1.01)	1.00 (0.99-1.01)	1.00 (0.99-1.01)
Sex							
Female	1.07 (0.84-1.36)	1.24 (0.97-1.57)	1.08 (0.85-1.37)	0.93 (0.73-1.18)	1.17 (0.92-1.49)	1.31 (1.02-1.67)	1.14 (0.89-1.44)
Male	1 [Reference]	1 [Reference]	1 [Reference]	1 [Reference]	1 [Reference]	1 [Reference]	1 [Reference]
Race and ethnicity							
Alaska Native or American Indian, Asian, or Native Hawaiian or Pacific Islander	0.91 (0.54-1.53)	0.59 (0.35-0.99)	0.56 (0.33-0.93)	1.00 (0.59-1.68)	0.80 (0.47-1.34)	0.93 (0.56-1.53)	0.63 (0.38-1.05)
Black or African American	0.79 (0.59-1.06)	0.88 (0.66-1.18)	0.89 (0.66-1.19)	0.92 (0.69-1.22)	0.95 (0.71-1.26)	0.73 (0.54-0.98)	0.85 (0.63-1.13)
Hispanic or Latino	1.05 (0.78-1.41)	0.83 (0.62-1.11)	0.98 (0.73-1.31)	0.99 (0.73-1.33)	0.84 (0.62-1.14)	0.88 (0.65-1.19)	1.05 (0.78-1.41)
White	1 [Reference]	1 [Reference]	1 [Reference]	1 [Reference]	1 [Reference]	1 [Reference]	1 [Reference]
Educational attainment	0.86 (0.75-0.99)	0.89 (0.77-1.02)	0.83 (0.73-0.96)	0.85 (0.74-0.98)	0.90 (0.78-1.04)	0.85 (0.74-0.99)	0.84 (0.73-0.96)
Income	0.94 (0.83-1.05)	0.98 (0.87-1.10)	1.03 (0.91-1.16)	0.94 (0.84-1.06)	0.99 (0.88-1.12)	0.98 (0.87-1.11)	0.93 (0.82-1.04)
English proficiency							
Very well	1 [Reference]	1 [Reference]	1 [Reference]	1 [Reference]	1 [Reference]	1 [Reference]	1 [Reference]
Well, not well, not at all	0.52 (0.35-0.76)	0.75 (0.51-1.09)	0.58 (0.39-0.85)	0.40 (0.27-0.59)	0.61 (0.40-0.91)	0.61 (0.41-0.92)	0.49 (0.33-0.73)
Has a primary care clinician							
Yes	1 [Reference]	1 [Reference]	1 [Reference]	1 [Reference]	1 [Reference]	1 [Reference]	1 [Reference]
No	0.59 (0.46-0.75)	0.65 (0.51-0.82)	0.60 (0.47-0.76)	0.60 (0.47-0.76)	0.65 (0.51-0.83)	0.63 (0.49-0.81)	0.71 (0.56-0.91)
Digital health literacy							
Using technology to process health information	0.88 (0.59-1.33)	0.72 (0.47- 1.11)	0.75 (0.50-1.12)	0.78 (0.52-1.17)	0.85 (0.56-1.28)	0.74 (0.48-1.14)	0.76 (0.50-1.17)
Understanding of health concepts and language	1.37 (0.91-2.05)	1.26 (0.84-1.91)	1.11 (0.73- 1.68)	1.43 (0.95-2.16)	2.01 (1.32-3.05)	1.46 (0.96-2.22)	1.35 (0.89-2.06)
Ability to actively engage with digital services	0.80 (0.57-1.14)	0.78 (0.55-1.11)	1.42 (0.99-2.02)	0.82 (0.58-1.16)	1.03 (0.72-1.46)	0.71 (0.49-1.01)	0.77 (0.53-1.10)
Feel safe and in control	0.91 (0.68-1.22)	1.58 (1.17-2.13)	1.40 (1.06-1.86)	1.32 (0.99-1.75)	1.33 (1.00-1.76)	1.51 (1.12-2.04)	1.51 (1.12-2.03)
Motivated to engage with digital services	1.10 (0.71-1.69)	1.31 (0.84-2.03)	1.06 (0.67-1.65)	1.07 (0.69-1.66)	1.03 (0.65-1.63)	1.00 (0.64-1.57)	1.22 (0.77-1.92)
Access to digital services that work	2.29 (1.50-3.49)	1.42 (0.91-2.21)	1.36 (0.87-2.12)	1.72 (1.12-2.65)	1.27 (0.82-1.95)	1.62 (1.03-2.53)	1.36 (0.85-2.17)
Digital services that suit individual needs	1.26 (0.90-1.76)	1.70 (1.20-2.41)	1.29 (0.92-1.81)	1.38 (0.99-1.92)	1.14 (0.81-1.61)	1.86 (1.32-2.62)	1.59 (1.12-2.25)

Abbreviations: AOR, adjusted odds ratio; MHSVI, Minority Health Social Vulnerability Index; PCC, patient-centered communication.

^a^ Based on imputed data.

^b^ Logistic regression analysis modeled the probability of 1 equal to always. Models adjusted for sexual orientation, health insurance, general and mental health, presence of underlying clinical conditions, history of COVID-19 infection, past-year in-person visit with health care clinician, lifestyle factors (ie, body mass index; physical activity; past-month cigarette smoking, e-cigarette use, marijuana or cannabis use; alcohol misuse), device and internet access.

^c^ PCC 1, gives you the chance to ask questions; PCC 2, gives attention to your feelings; PCC 3, involves you in decisions; PCC 4, makes sure you understand; PCC 5, explains things; PCC 6, spends enough time; and PCC 7, helps deal with uncertainty.

**Table 5.** Factors Associated With Optimal PCC Among 1249 Participants Who Had 1 or More Telehealth Visits in the Past Year and Resided in the MHSVI Least-Vulnerable Counties^a^

Characteristic	AOR (95% CI)^b^						
	PCC 1^c^	PCC 2^c^	PCC 3^c^	PCC 4^c^	PCC 5^c^	PCC 6^c^	PCC 7^c^
Age	1.00 (0.99-1.01)	1.00 (0.99-1.01)	1.01 (1.00-1.02)	1.01 (1.01-1.02)	1.01 (1.00-1.02)	1.01 (1.00-1.02)	1.00 (0.99-1.01)
Sex							
Female	1.33 (1.01-1.74)	1.50 (1.15-1.96)	1.51 (1.16-1.97)	1.62 (1.24-2.12)	1.42 (1.09-1.84)	1.25 (0.95-1.64)	1.19 (0.90-1.56)
Male	1 [Reference]	1 [Reference]	1 [Reference]	1 [Reference]	1 [Reference]	1 [Reference]	1 [Reference]
Race and ethnicity							
Alaska Native or American Indian, Asian, or Native Hawaiian or Pacific Islander	0.51 (0.28-0.92)	0.42 (0.22-0.80)	0.47 (0.24-0.92)	0.61 (0.34-1.12)	0.47 (0.26-0.84)	0.31 (0.15-0.64)	0.32 (0.17-0.63)
Black or African American	1.13 (0.69-1.85)	0.94 (0.56-1.57)	1.18 (0.73-1.89)	1.50 (0.93-2.43)	1.74 (1.03-2.94)	0.82 (0.50-1.35)	1.38 (0.84-2.28)
Hispanic or Latino	1.13 (0.71-1.79)	0.91 (0.57-1.43)	1.02 (0.65-1.60)	1.07 (0.68-1.69)	0.93 (0.60-1.45)	0.88 (0.56-1.38)	0.88 (0.55-1.39)
White	1 [Reference]	1 [Reference]	1 [Reference]	1 [Reference]	1 [Reference]	1 [Reference]	1 [Reference]
Educational level	1.00 (0.84-1.19)	0.99 (0.83-1.19)	0.87 (0.73-1.04)	0.86 (0.72-1.03)	0.92 (0.77-1.09)	0.88 (0.73-1.05)	0.88 (0.74-1.06)
Income	0.99 (0.87-1.13)	0.97 (0.85-1.12)	0.97 (0.84-1.11)	1.02 (0.89-1.17)	1.03 (0.90-1.17)	0.99 (0.86-1.13)	0.95 (0.83-1.09)
English proficiency							
Very well	1 [Reference]	1 [Reference]	1 [Reference]	1 [Reference]	1 [Reference]	1 [Reference]	1 [Reference]
Well, not well, not at all	0.28 (0.15-0.50)	0.48 (0.28-0.84)	0.30 (0.17-0.53)	0.35 (0.20-0.59)	0.31 (0.18-0.55)	0.47 (0.27-0.83)	0.39 (0.21-0.69)
Has a primary care clinician							
Yes	1 [Reference]	1 [Reference]	1 [Reference]	1 [Reference]	1 [Reference]	1 [Reference]	1 [Reference]
No	0.61 (0.47-0.81)	0.61 (0.46-0.80)	0.57 (0.43-0.75)	0.50 (0.38-0.65)	0.63 (0.48-0.83)	0.64 (0.49-0.85)	0.62 (0.47-0.82)
Digital health literacy							
Using technology to process health information	0.95 (0.58-1.53)	0.79 (0.48-1.32)	0.63 (0.39-1.00)	0.54 (0.34-0.86)	0.41 (0.25-0.68)	0.78 (0.47-1.28)	0.53 (0.32-0.87)
Understanding of health concepts and language	1.21 (0.78-1.88)	1.52 (0.96-2.41)	1.33 (0.86-2.05)	1.73 (1.10-2.70)	2.16 (1.37-3.40)	1.20 (0.75-1.93)	1.67 (1.05-2.65)
Ability to actively engage with digital services	1.09 (0.73-1.62)	0.98 (0.66-1.47)	1.47 (0.98-2.19)	1.45 (0.98-2.15)	1.52 (1.02-2.26)	1.12 (0.74-1.70)	0.90 (0.60-1.37)
Feel safe and in control	1.42 (1.05-1.92)	1.46 (1.07-1.98)	1.34 (1.00-1.80)	1.42 (1.07-1.90)	1.11 (0.82-1.50)	1.76 (1.27-2.44)	1.43 (1.04-1.97)
Motivated to engage with digital services	0.97 (0.58-1.61)	1.30 (0.79-2.15)	1.31 (0.80-2.12)	1.23 (0.78-1.92)	1.39 (0.85-2.28)	1.12 (0.67-1.89)	1.69 (1.02-2.79)
Access to digital services that work	2.09 (1.31-3.36)	2.03 (1.25-3.29)	1.80 (1.12-2.89)	1.26 (0.79-2.02)	1.63 (1.02-2.62)	1.56 (0.94-2.59)	2.11 (1.27-3.51)
Digital services that suit individual needs	1.07 (0.71-1.62)	1.06 (0.70-1.58)	1.13 (0.74-1.71)	1.24 (0.83-1.85)	1.16 (0.77-1.75)	1.23 (0.80-1.90)	1.05 (0.69-1.58)

Abbreviations: AOR, adjusted odds ratio; MHSVI, Minority Health Social Vulnerability Index; PCC, patient-centered communication.

^a^ Based on imputed data.

^b^ Logistic regression analysis modeled the probability of 1 equal to always. Models adjusted for sexual orientation, health insurance, general and mental health, presence of underlying clinical conditions, history of COVID-19 infection, past-year in-person visit with health care clinician, lifestyle factors (ie, body mass index; physical activity; past-month cigarette smoking, e-cigarette use, marijuana or cannabis use; alcohol misuse), device and internet access.

^c^ PCC 1, gives you the chance to ask questions; PCC 2, gives attention to your feelings; PCC 3, involves you in decisions; PCC 4, makes sure you understand; PCC 5, explains things; PCC 6, spends enough time; and PCC 7, helps deal with uncertainty.

Results for all covariates, including PMs, appear in eTables 7 though 9 and eTables 10 though 12 in Supplement 1 for imputed and complete case analyses, respectively. Sensitivity analyses showed that model results based on complete case analysis were largely consistent with those based on the multiple imputation analysis.

## Discussion

This cross-sectional online survey study examined individual-level characteristics associated with optimal PCC in telehealth settings and whether these associations varied by county-level vulnerability. Overall, 38.8% to 49.8% of participants reported optimal PCC. Self-reported optimal PCC was largely equivalent by race and ethnicity and county-level vulnerability. English nonproficiency and higher educational attainment were associated with suboptimal PCC. English proficiency differences persisted in both most- and least-vulnerable counties, whereas educational attainment differences were observed in the most-vulnerable counties only. With upward trends in telehealth use,^19,20,41^ our results suggest that telehealth can potentially facilitate optimal PCC necessary for ensuring high-quality health care.

We found largely nonexistent associations between self-identifying as Hispanic or Latino and Black or African American and optimal PCC and between county-level vulnerability and PCC. There were almost no associations between the individual themes of the MHSVI and PCC, including 2 health themes. Noteworthy, Alaska Native or American Indian, Asian, and Native Hawaiian or Pacific Islander individuals, grouped as other race, reported PCC disparities that persisted across both MHSVI strata, although these disparities were observed for more PCC items in the least-vulnerable counties. Evidence shows that Asian patients have reported worse PCC and quality of care compared with White and other racial and ethnic minority populations.^47,48^ The “model minority” health stereotype of Asian Americans may explain this, as clinicians may be underestimating their health care needs.^49^

English nonproficient participants and those with higher educational attainment self-reported receiving suboptimal PCC. While results for English proficiency were evident regardless of county-level vulnerability, greater educational attainment was associated with less than optimal PCC in the MHSVI most-vulnerable counties only. These results suggest that language-based communication differences documented in in-person encounters carry over to telehealth settings.^50,51^ Furthermore, evidence has shown higher education being associated with patient dissatisfaction and poorer perceived communication quality.^22,42^ Potential explanations include having preconceived notions of care informed by patients’ online health information–seeking behaviors, which can affect the patient-clinician relationship.^52,53^ In addition, evidence shows that limited DHL can be a barrier to uptake of digital-based services.^41,54,55^ We found that higher scores on most DHL domains were associated with optimal PCC. The only negative association was documented for the DHL domain of using technology to process health information. This finding could be attributed to the higher chances of experiencing poor PCC with increased use of digital technologies for health purposes. Patient-facing DHL interventions are needed to facilitate the use of technology-based health services.^56,57^

Spending enough time with patients and helping manage uncertainty were the PCC items with the lowest percentages of optimal reporting by participants. This is consistent with the results of a study conducted in 2011 through 2013 involving a nationally representative sample of US adults in which helping with uncertainty (42%), addressing feelings (45%), and spending enough time (46%) had the lowest percentages of optimal reporting by participants of all 7 PCC items.^42^ Although there was no explicit indication that the results were based on in-person medical encounters, one can presume that given the low rates of telehealth use at the time (eg, 0.26% among Medicaid beneficiaries in 2011).^58^ Visit length is set by the primary care system, approximately 15 to 20 minutes per visit,^59^ with mixed evidence regarding differences in visit length by patient demographic groups.^60,61,62^ However, consistent with the published literature, perceived inadequacy of time spent was evident in our study, which carries implications for patient satisfaction and patient-clinician relationships^63,64^ that outweigh objective visit length.^65^ Some studies indicate visit length is inadequate, especially for meeting the needs of patients with complex medical and socioeconomic profiles.^66,67^ Consistent with our findings, evidence shows that clinicians may be underprepared to engage in emotional conversations or manage patient feelings and uncertainties.^22,42,68,69^ The affective dimension of patient-clinician communication is valued by patients, warranting strategies for its improvement generally and in telehealth settings.^6,21,70^ Potential strategies to improve telehealth-based PCC include alerting clinicians to address PCC functions (eg, encouraging patients to ask questions) and educating clinicians and patients on best telehealth practices (eg, looking at the camera to mimic eye-contact).^35^ Research should gauge clinicians’ input on strategies that can equip them to achieve optimal telehealth-based PCC.^26,27^

### Strengths and Limitations

The strengths of this study included examining individual-level characteristics associated with optimal PCC and how these associations differed by a comprehensive index of county-level vulnerability.^71^ We used a validated measure that captured PCC functions, extended its use to telehealth settings, and reported factors associated with individual PCC items rather than in aggregate. Research should replicate our findings among nationally representative samples and parse out individual and county-level factors that can affect telehealth-based PCC, their interactions, and underlying mechanisms.

The limitations of this study included the use of self-reported PCC,^6,72^ which is subject to recall and reporting biases (eg, responses based on memorable encounters rather than averaged over multiple ones). Most participants had both a telehealth and in-person health care visit; thus, their telehealth-based PCC perceptions could have been influenced by in-person visits. We did not distinguish between audio and video settings vs audio-only settings and did not account for several factors that can affect PCC.^6,61^ We combined Alaska Native or American Indian, Asian, and Native Hawaiian or Pacific Islander into 1 group. Our results are dependent on measures used to capture PCC and community-level factors^73,74^ and may be unique to 2022 when data collection occurred. The results are not generalizable to the US population, and we cannot infer causal relationships. Some variables showed inconsistent associations with PCC across the imputed and complete case models. We did not adjust for multiple testing.^75^ People with limited internet access or digital competencies may not have been able to participate. The survey was available only in English, and we did not have access to certain information (eg, characteristics of people who did not respond to the survey).

## Conclusions

In this cross-sectional study, approximately half of participants perceived PCC in telehealth settings as optimal, indicating that its core functions were mostly met, although improvements are needed for time spent with patients and uncertainty management. While results show the promise of telehealth in facilitating optimal PCC, mitigating differences in PCC by language proficiency and educational attainment and among Asian Americans should be prioritized, especially in most vulnerable counties. Individual and county factors are essential to improve telehealth-based PCC.

## Notes

Supplement 1. **eTable 1.** Differences between participants excluded and included in the analyses, between those who had (vs. did not have) telehealth visits in the past year, and between those with any missing data and those with complete data **eTable 2.** Multinomial logit model for type of healthcare visit, N = 5444 **eTable 3.** Associations between individual themes of the Minority Health Social Vulnerability Index and optimal patient-centered communication **eTable 4.** Factors associated with optimal patient-centered communication among those who had 1 or more telehealth visits in the past year, N = 2714, sensitivity analysis with self-identifying Asian participants as a separate racial/ethnic group **eTable 5.** Factors associated with optimal patient-centered communication among those who had 1 or more telehealth visits in the past year and resided in MHSVI most vulnerable counties, n = 1471, sensitivity analysis with self-identifying Asian participants as a separate racial/ethnic group **eTable 6.** Factors associated with optimal patient-centered communication among those who had 1 or more telehealth visits in the past year and resided in MHSVI least vulnerable counties, n = 1243, sensitivity analysis with self-identifying Asian participants as a separate racial/ethnic group **eTable 7.** Factors associated with optimal patient-centered communication among those who had 1 or more telehealth visits in the past year, N = 2754, imputed data **eTable 8.** Factors associated with optimal patient-centered communication among those who had 1 or more telehealth visits in the past year and resided in MHSVI most vulnerable counties, n = 1505, imputed data **eTable 9.** Factors associated with optimal patient-centered communication among those who had 1 or more telehealth visits in the past year and resided in MHSVI least vulnerable counties, n = 1249, imputed data **eTable 10.** Factors associated with optimal patient-centered communication among those who had 1 or more telehealth visits in the past year, N = 2165, complete case **eTable 11.** Factors associated with optimal patient-centered communication among those who had 1 or more telehealth visits in the past year and resided in MHSVI most vulnerable counties, n = 1165, complete case **eTable 12.** Factors associated with optimal patient-centered communication among those who had 1 or more telehealth visits in the past year and resided in MHSVI least vulnerable counties, n = 1000, complete case **eFigure.** Adjusted odds ratios for participants’ reporting of optimal patient-centered communication among those who had 1 or more telehealth visits in the past year, N = 2754

Supplement 2.
Data Sharing Statemen
