# Supplementary material for: Patient-Centered Communication in Telehealth Settings
Source: JAMA Netw Open. 2026 Jan 29;9(1):e2556291. doi: 10.1001/jamanetworkopen.2025.56291 (PMC12856684; doi:10.1001/jamanetworkopen.2025.56291)
Supplement: Supplement 1. — eTable 1. Differences between participants excluded and included in the analyses, between those who had (vs. did not have) telehealth visits in the past year, and between those with any missing data and those with complete data eTable 2. Multinomial logit model for type of healthcare visit, N = 5444 eTable 3. Associations between individual themes of the Minority Health Social Vulnerability Index and optimal patient-centered communication eTable 4. Factors associated with optimal patient-centered communication among those who had 1 or more telehealth visits in the past year, N = 2714, sensitivity analysis with self-identifying Asian participants as a separate racial/ethnic group eTable 5. Factors associated with optimal patient-centered communication among those who had 1 or more telehealth visits in the past year and resided in MHSVI most vulnerable counties, n = 1471, sensitivity analysis with self-identifying Asian participants as a separate racial/ethnic group eTable 6. Factors associated with optimal patient-centered communication among those who had 1 or more telehealth visits in the past year and resided in MHSVI least vulnerable counties, n = 1243, sensitivity analysis with self-identifying Asian participants as a separate racial/ethnic group eTable 7. Factors associated with optimal patient-centered communication among those who had 1 or more telehealth visits in the past year, N = 2754, imputed data eTable 8. Factors associated with optimal patient-centered communication among those who had 1 or more telehealth visits in the past year and resided in MHSVI most vulnerable counties, n = 1505, imputed data eTable 9. Factors associated with optimal patient-centered communication among those who had 1 or more telehealth visits in the past year and resided in MHSVI least vulnerable counties, n = 1249, imputed data eTable 10. Factors associated with optimal patient-centered communication among those who had 1 or more telehealth visits in the past year, N = 2165, [file jamanetwopen-e2556291-s001.pdf]

Supplementary Online Content

Tesfaye L, Ansari Z, Curry M, Buckman D, Pérez-Stable EJ, El-Toukhy S. Patient-centered communication in telehealth settings. *JAMA Netw Open*. 2026;9(1):e2556291. doi:10.1001/jamanetworkopen.2025.56291

eTable 1. Differences between participants excluded and included in the analyses, between those who had (vs. did not have) telehealth visits in the past year, and between those with any missing data and those with complete data

eTable 2. Multinomial logit model for type of healthcare visit, N = 5444

eTable 3. Associations between individual themes of the Minority Health Social Vulnerability Index and optimal patient-centered communication

eTable 4. Factors associated with optimal patient-centered communication among those who had 1 or more telehealth visits in the past year, N = 2714, sensitivity analysis with self-identifying Asian participants as a separate racial/ethnic group

eTable 5. Factors associated with optimal patient-centered communication among those who had 1 or more telehealth visits in the past year and resided in MHSVI most vulnerable counties, n = 1471, sensitivity analysis with self-identifying Asian participants as a separate racial/ethnic group

eTable 6. Factors associated with optimal patient-centered communication among those who had 1 or more telehealth visits in the past year and resided in MHSVI least vulnerable counties, n = 1243, sensitivity analysis with self-identifying Asian participants as a separate racial/ethnic group

eTable 7. Factors associated with optimal patient-centered communication among those who had 1 or more telehealth visits in the past year, N = 2754, imputed data

eTable 8. Factors associated with optimal patient-centered communication among those who had 1 or more telehealth visits in the past year and resided in MHSVI most vulnerable counties, n = 1505, imputed data

eTable 9. Factors associated with optimal patient-centered communication among those who had 1 or more telehealth visits in the past year and resided in MHSVI least vulnerable counties, n = 1249, imputed data

eTable 10. Factors associated with optimal patient-centered communication among those who had 1 or more telehealth visits in the past year, N = 2165, complete case

eTable 11. Factors associated with optimal patient-centered communication among those who had 1 or more telehealth visits in the past year and resided in MHSVI most vulnerable counties, n = 1165, complete case

© 2026 Tesfaye L et al. *JAMA Network Open.*

eTable 12. Factors associated with optimal patient-centered communication among those who had 1 or more telehealth visits in the past year and resided in MHSVI least vulnerable counties, n = 1000, complete case

eFigure. Adjusted odds ratios for participants' reporting of optimal patient-centered communication among those who had 1 or more telehealth visits in the past year, N = 2754

This supplementary material has been provided by the authors to give readers additional information about their work.

© 2026 Tesfaye L et al. *JAMA Network Open*.

eTable 1. Differences between participants excluded and included in the analyses, between those who had (vs. did not have) telehealth visits in the past year, and between those with any missing data and those with complete data

Characteristics	Excluded (n= 115)[a]	Included (n= 5444)[a]	*p*	Had telehealth visit (*n*= 2754)[b]	Did not have telehealth visit (*n*= 2690)[b]	*p*	With missing data (*n*= 589)[c]	With complete data (*n*= 2165)[c]	*p*
	**No. (%)**	**No. (%)**		**No. (%)**	**No. (%)**		**No. (%)**	**No. (%)**	
**Age[d]**			NA			**<0.001**			**<.0001**
18-29	NA	1222 (22.4)		668 (24.2)	554 (20.5)		244 (41.4)	424 (19.5)	
30-44	NA	1517 (27.8)		807 (29.3)	710 (26.3)		158 (26.8)	649 (29.9)	
45-59	NA	1387 (25.4)		711 (25.8)	676 (25.1)		111 (18.8)	600 (27.7)	
≥60	NA	1318 (24.2)		568 (20.6)	750 (27.8)		76 (12.9)	492 (22.7)	
**Sex**			0.37			**<0.001**			**<.0001**
Female	57 (49.5)	2927 (53.7)		1568 (56.9)	1359 (50.5)		378 (64.1)	1190 (54.9)	
Male	58 (50.4)	2517 (46.2)		1186 (43.0)	1331 (49.4)		211 (35.8)	975 (45.0)	
**Sexual orientation**			**0.001**			**<0.001**			**0.0004**
Heterosexual	90 (78.2)	4746 (87.1)		2384 (86.5)	2449 (91.0)		429 (82.6)	1914 (88.4)	
Gay, lesbian, bisexual	24 (20.8)	577 (10.6)		370 (13.4)	241 (8.95)		90 (17.3)	251 (11.5)	
Prefer not to answer / I don't know	1 (0.8)	121 (2.2)							
**Race and/or ethnicity**			0.33			**<0.001**			**<.0001**
AIAN	0 (0)	58 (1.0)		32 (1.1)	26 (0.9)		2 (0.3)	30 (1.3)	
Asian or NHPI	2 (1.7)	208 (3.8)		106 (3.8)	102 (3.7)		23 (3.9)	83 (3.8)	
Black or AA	14 (12.1)	798 (14.6)		465 (16.8)	333 (12.3)		138 (23.4)	327 (15.1)	
Hispanic or Latino	23 (20.0)	838 (15.3)		501 (18.1)	337 (12.5)		148 (25.1)	353 (16.3)	
White	76 (66.0)	3542 (65.0)		1650 (59.9)	1892 (70.3)		278 (47.2)	1372 (63.3)	
**Education**			0.60			0.50			0.06
<High school	11 (9.5)	279 (5.1)		144 (5.2)	140 (5.2)		50 (8.8)	91 (4.2)	
High school graduate	39 (33.9)	1444 (26.5)		745 (27.0)	714 (26.5)		192 (34.0)	544 (25.1)	
Vocational school, some college	34 (29.5)	1978 (36.3)		1015 (36.8)	976 (36.2)		191 (33.8)	816 (37.6)	
College graduate or higher	28 (24.3)	1700 (31.2)		850 (30.8)	859 (31.9)		131 (23.2)	714 (32.9)	

© 2026 Tesfaye L et al. *JAMA Network Open*.

Characteristics	Excluded (n= 115)[a]	Included (n= 5444)[a]	*p*	Had telehealth visit (*n*= 2754)[b]	Did not have telehealth visit (*n*= 2690)[b]	*p*	With missing data (*n*= 589)[c]	With complete data (*n*= 2165)[c]	*p*
	**No. (%)**	**No. (%)**		**No. (%)**	**No. (%)**		**No. (%)**	**No. (%)**	
Prefer not to answer / I don't know	3 (2.6)	43 (0.7)							
**Income**			0.83			**0.04**			0.21
<$20,000	27 (23.4)	1215 (22.3)		708 (25.6)	622 (23.1)		167 (38.1)	493 (22.7)	
$20,000 to $49,999	34 (29.5)	1588 (29.1)		856 (31.0)	854 (31.7)		132 (30.1)	672 (31.0)	
$50,000 to $74,999	20 (17.3)	891 (16.3)		478 (17.3)	471 (17.5)		52 (11.8)	404 (18.6)	
≥$75,000	27 (23.4)	1383 (25.4)		712 (25.8)	743 (27.6)		87 (19.8)	596 (27.5)	
Prefer not to answer/I don't know	7 (6.0)	367 (6.7)							
**English proficiency**			**<0.001**			**<0.001**			**<.0001**
Very well	92 (80.0)	4984 (91.5)		2466 (89.5)	2518 (93.6)		444 (75.3)	2022 (93.3)	
Well, not well, not at all	23 (20.0)	460 (8.4)		288 (10.4)	172 (6.3)		145 (24.6)	143 (6.6)	
**Health insurance**			0.92			**<0.001**			**0.0001**
Insured	102 (88.7)	4844 (88.9)		2539 (92.1)	2305 (85.6)		521 (88.4)	2018 (93.2)	
Uninsured	13 (11.3)	600 (11.0)		215 (7.8)	385 (14.3)		68 (11.5)	147 (6.7)	
**General health**			0.32			**<0.001**			0.841
Excellent, very good, good	83 (72.1)	4144 (76.1)		2011 (73.0)	2133 (79.2)		432 (73.3)	1579 (72.9)	
Fair, poor	32 (27.8)	1300 (23.8)		743 (26.9)	557 (20.7)		157 (26.6)	586 (27.0)	
**Mental health**			0.15			**<0.001**			0.935
Excellent, very good, good	77 (66.9)	3972 (72.9)		1932 (70.1)	2040 (75.8)		414 (70.2)	1518 (70.1)	
Fair, poor	38 (33.0)	1472 (27.0)		822 (29.8)	650 (24.1)		175 (29.7)	647 (29.8)	
**Has a primary care clinician**			0.36			**<0.001**			**<.0001**
Yes	60 (52.1)	2609 (47.9)		1524 (55.3)	1085 (40.3)		273 (46.3)	1251 (57.7)	
No	55 (47.8)	2835 (52.0)		1230 (44.6)	1605 (59.6)		316 (53.6)	914 (42.2)	

© 2026 Tesfaye L et al. *JAMA Network Open*.

Characteristics	Excluded (n= 115)[a]	Included (n= 5444)[a]	*p*	Had telehealth visit (*n*= 2754)[b]	Did not have telehealth visit (*n*= 2690)[b]	*p*	With missing data (*n*= 589)[c]	With complete data (*n*= 2165)[c]	*p*
	**No. (%)**	**No. (%)**		**No. (%)**	**No. (%)**		**No. (%)**	**No. (%)**	
**Presence of underlying clinical conditions**			0.71			**<0.001**			**<.0001**
Yes	38 (33.0)	1887 (34.6)		1058 (38.4)	829 (30.8)		170 (28.8)	888 (41.0)	
No	77 (66.9)	3557 (65.3)		1696 (61.5)	1861 (69.1)		419 (71.1)	1277 (58.9)	
**Past year in-person visit(s) with healthcare clinician**			0.06			**<0.001**			0.992
0 times	16 (13.9)	1146 (21.0)		220 (7.9)	926 (34.4)		47 (7.9)	173 (7.9)	
1 or more times	99 (86.0)	4298 (78.9)		2534 (92.0)	1764 (65.5)		542 (92.0)	1992 (92.0)	
**History of COVID-19 infection**			0.08			**<0.001**			**0.0027**
Yes	40 (34.7)	1396 (25.6)		843 (30.6)	596 (22.1)		127 (24.4)	675 (31.1)	
No	72 (62.6)	3911 (71.8)		1911 (69.3)	2094 (77.8)		392 (75.5)	1490 (68.8)	
Prefer not to answer/I don't know	3 (2.6)	137 (2.5)							
**Body mass index[e]**			0.06			0.11			0.271
Healthy (18.5 to <25)	37 (32.1)	1385 (25.4)		776 (28.1)	814 (30.2)		57 (31.8)	606 (27.9)	
Unhealthy (<18.5 or ≥25)	58 (50.4)	3329 (61.1)		1978 (71.8)	1876 (69.7)		122 (68.1)	1559 (72.0)	
Unknown	20 (17.3)	730 (13.4)					410	0	
**Physical activity**			**0.04**			**<0.001**			0.225
Sufficient (≥150 minutes per week)	84 (73.0)	3494 (64.1)		1898 (68.9)	1596 (59.3)		418 (70.9)	1480 (68.3)	
Insufficient (<150 minutes per week)	31 (26.9)	1950 (35.8)		856 (31.0)	1094 (40.6)		171 (29.0)	685 (31.6)	
**Past month cigarette smoking**			**<0.001**			**<0.001**			**<.0001**
Yes	60 (52.1)	1601 (29.4)		1019 (37.0)	582 (21.6)		264 (44.8)	755 (34.8)	
No	55 (47.8)	3843 (70.5)		1735 (63.0)	2108 (78.3)		325 (55.1)	1410 (65.1)	

© 2026 Tesfaye L et al. *JAMA Network Open*.

Characteristics	Excluded (n= 115)[a]	Included (n= 5444)[a]	*p*	Had telehealth visit (*n*= 2754)[b]	Did not have telehealth visit (*n*= 2690)[b]	*p*	With missing data (*n*= 589)[c]	With complete data (*n*= 2165)[c]	*p*
	**No. (%)**	**No. (%)**		**No. (%)**	**No. (%)**		**No. (%)**	**No. (%)**	
**Past month e-cigarette use**			**<0.001**			**<0.001**			**<.0001**
Yes	45 (39.1)	918 (16.8)		674 (24.4)	244 (9.0)		220 (37.3)	454 (20.9)	
No	70 (60.8)	4526 (83.1)		2080 (75.5)	2446 (90.9)		369 (62.6)	1711 (79.0)	
**Alcohol misuse[d]**			NA			0.10			**<.0001**
Yes	NA	350 (6.4)		185 (6.7)	165 (6.1)		39 (6.6)	146 (6.7)	
No	NA	4848 (89.0)		2430 (88.2)	2418 (89.8)		487 (82.6)	1943 (89.7)	
Not applicable (<21 years)	NA	246 (4.5)		139 (5.0)	107 (3.9)		63 (10.7)	76 (3.5)	
**Past month marijuana or cannabis use**			**<0.001**			**<0.001**			**<.0001**
Yes	52 (45.2)	1350 (24.8)		878 (31.8)	472 (17.5)		245 (41.6)	633 (29.2)	
No	63 (54.7)	4094 (75.2)		1876 (68.1)	2218 (82.4)		344 (58.4)	1532 (70.7)	
**Device access**			0.18			0.61			**<.0001**
Video and audio access	110 (95.6)	5334 (97.9)		2700 (98.0)	2634 (97.9)		551 (93.5)	2149 (99.2)	
Audio access only	1 (0.8)	31 (0.5)		13 (0.4)	18 (0.6)		9 (1.5)	4 (0.1)	
No access	4 (3.4)	79 (1.4)		41 (1.4)	38 (1.4)		29 (4.9)	12 (0.5)	
**Internet access**			0.33			0.25			**<.0001**
Yes	114 (99.1)	5325 (97.8)		2700 (98.0)	2625 (97.5)		565 (95.9)	2135 (98.6)	
No	1 (0.8)	119 (2.1)		54 (1.9)	65 (2.4)		24 (4.0)	30 (1.3)	
**Digital health literacy, M (SE)**									
Using technology to process health information	2.94 (0.05)	2.91 (0.007)	0.53	3.02 (0.01)	2.81 (0.01)	**<0.001**	2.91 (0.02)	3.04 (0.01)	**<0.001**
Understanding of health concepts and language	2.96 (0.05)	2.98 (0.006)	0.66	3.05 (0.01)	2.93 (0.01)	**<0.001**	2.93 (0.02)	3.08 (0.01)	**<0.001**

© 2026 Tesfaye L et al. *JAMA Network Open*.

Characteristics	Excluded (n= 115)[a]	Included (n= 5444)[a]	*p*	Had telehealth visit (*n*= 2754)[b]	Did not have telehealth visit (*n*= 2690)[b]	*p*	With missing data (*n*= 589)[c]	With complete data (*n*= 2165)[c]	*p*
	**No. (%)**	**No. (%)**		**No. (%)**	**No. (%)**		**No. (%)**	**No. (%)**	
Ability to actively engage with digital services	2.97 (0.05)	2.95 (0.007)	0.74	3.02 (0.01)	2.88 (0.01)	**<0.001**	2.91 (0.02)	3.05 (0.01)	**<0.001**
Feel safe and in control	2.87 (0.05)	2.83 (0.008)	0.47	2.90 (0.01)	2.75 (0.01)	**<0.001**	2.81 (0.02)	2.93 (0.01)	**<0.001**
Motivated to engage with digital services	2.93 (0.05)	2.88 (0.007)	0.40	2.99 (0.01)	2.78 (0.01)	**<0.001**	2.87 (0.02)	3.02 (0.01)	**<0.001**
Access to digital services that work	2.96 (0.04)	2.92 (0.006)	0.46	3.01 (0.01)	2.84 (0.01)	**<0.001**	2.89 (0.02)	3.04 (0.01)	**<0.001**
Digital services that suit individual needs	2.86 (0.05)	2.80 (0.008)	0.30	2.92 (0.01)	2.69 (0.01)	**<0.001**	2.84 (0.02)	2.93 (0.01)	**0.001**

Abbreviations: AA= African American; AIAN= American Indian, Alaska Native; NHPI= Native Hawaiian, Pacific Islander; M=Mean, SE= Standard Error
Bolded cells represent significant results at $p<0.05$.
[a] Based on a sample of 5559 participants.
[b] Based on a sample of 5444 participants.
[c] Based on a sample of 2754 participants.
[d] Excluded participants inconsistently reported their age. Frequencies, percentages, and chi-square were not produced.
[e] Body mass index was derived from height and weight with 0.25% exclusive missingness on height (*n*= 14) and 13.15% missingness on weight (*n*= 716).

© 2026 Tesfaye L et al. *JAMA Network Open*.

eTable 2. Multinomial logit model for type of healthcare visit, *N* = 5444[a]

Characteristics	Telehealth visits only (n=220)	In-person & telehealth visits (n=2534)	In-person visits only (n=1764)
	**aOR (95% CI)**	**aOR (95% CI)**	**aOR (95% CI)**
**Age**	0.99 (0.98, 1.00)	0.99 (0.99, 1.00)	1.00 (0.99, 1.00)
**Sex**			
Female	1.25 (0.91, 1.71)	**1.28 (1.07, 1.54)**	1.21 (1.00, 1.46)
Male	Ref	Ref	Ref
**Sexual orientation**			
Heterosexual	Ref	Ref	Ref
Gay, lesbian, bisexual	0.73 (0.41, 1.31)	1.27 (0.93, 1.74)	0.97 (0.70, 1.34)
**Race and/or ethnicity**			
AIAN, Asian, NHPI	0.77 (0.38, 1.55)	0.84 (0.59, 1.22)	**0.57 (0.38, 0.85)**
Black or AA	1.30 (0.79, 2.12)	**1.79 (1.36, 2.37)**	1.24 (0.93, 1.65)
Hispanic or Latino	1.40 (0.87, 2.24)	**1.84 (1.40, 2.41)**	1.30 (0.98, 1.72)
White	Ref	Ref	Ref
**Education**	1.05 (0.87, 1.25)	1.09 (0.97, 1.22)	1.01 (0.90, 1.13)
**Income**	1.14 (0.96, 1.35)	**1.19 (1.08, 1.31)**	**1.23 (1.12, 1.36)**
**English proficiency**			
Very well	Ref	Ref	Ref
Well, not well, not at all	0.89 (0.48, 1.63)	**1.47 (1.07, 2.02)**	0.85 (0.59, 1.21)
**Health insurance**			
Insured	Ref	Ref	Ref
Uninsured	**0.35 (0.22, 0.57)**	**0.27 (0.21, 0.35)**	**0.32 (0.25, 0.42)**
**General health**			
Excellent, very good, good	Ref	Ref	Ref
Fair, poor	1.06 (0.70, 1.59)	1.22 (0.96, 1.56)	0.92 (0.71, 1.18)
**Mental health**			
Excellent, very good, good	Ref	Ref	Ref
Fair, poor	1.44 (1.00, 2.06)	1.11 (0.88, 1.39)	1.02 (0.81, 1.29)
**Has a primary care clinician**			
Yes	Ref	Ref	Ref
No	**0.32 (0.22, 0.46)**	**0.19 (0.15, 0.24)**	**0.26 (0.20, 0.32)**
**Presence of underlying clinical conditions**			
Yes	**1.84 (1.22, 2.79)**	**2.34 (1.85, 2.97)**	**2.26 (1.78, 2.86)**
No	Ref	Ref	Ref

© 2026 Tesfaye L et al. *JAMA Network Open*.

Characteristics	Telehealth visits only (n=220)	In-person & telehealth visits (n=2534)	In-person visits only (n=1764)
	aOR (95% CI)	aOR (95% CI)	aOR (95% CI)
**History of COVID-19 infection**			
Yes	**1.48 (1.04, 2.09)**	**1.72 (1.39, 2.12)**	1.21 (0.97, 1.50)
No	Ref	Ref	Ref
**Body mass index**			
Healthy (18.5 to <25)	Ref	Ref	Ref
Unhealthy (<18.5 or ≥25)	1.16 (0.80, 1.67)	1.19 (0.97, 1.45)	1.00 (0.81, 1.23)
**Physical activity**			
Sufficient (≥150 minutes per week)	Ref	Ref	Ref
Insufficient (<150 minutes per week)	**0.65 (0.46, 0.91)**	**0.67 (0.56, 0.81)**	**0.80 (0.66, 0.96)**
**Past month cigarette smoking**			
Yes	1.40 (0.92, 2.11)	**1.59 (1.27, 1.99)**	0.95 (0.75, 1.20)
No	Ref	Ref	Ref
**Past month e-cigarette use**			
Yes	**2.27 (1.39, 3.70)**	**2.44 (1.81, 3.28)**	1.21 (0.87, 1.68)
No	Ref	Ref	Ref
**Alcohol misuse**			
Yes	1.22 (0.67, 2.23)	0.94 (0.66, 1.35)	1.11 (0.77, 1.60)
No	Ref	Ref	Ref
Not applicable (<21 years)	0.79 (0.35, 1.74)	1.00 (0.64, 1.56)	0.99 (0.61, 1.60)
**Past month marijuana or cannabis use**			
Yes	0.89 (0.57, 1.38)	**1.40 (1.10, 1.77)**	0.99 (0.77, 1.27)
No	Ref	Ref	Ref
**Device access**			
Yes	Ref	Ref	Ref
No	0.45 (0.09, 2.07)	1.25 (0.62, 2.52)	0.47 (0.20, 1.13)
**Internet access**			
Yes	Ref	Ref	Ref
No	1.19 (0.46, 3.07)	0.67 (0.38, 1.19)	0.57 (0.29, 1.12)
**Digital health literacy**			
Using technology to process health information	1.77 (0.99, 3.19)	**1.66 (1.21, 2.27)**	1.35 (0.99, 1.84)
Understanding of health concepts and language	1.57 (0.91, 2.69)	1.09 (0.82, 1.46)	1.18 (0.88, 1.58)
Ability to actively engage with digital services	0.68 (0.44, 1.06)	**0.75 (0.57, 0.97)**	0.82 (0.63, 1.07)
Feel safe and in control	1.00 (0.70, 1.42)	1.02 (0.83, 1.25)	1.20 (0.98, 1.47)

© 2026 Tesfaye L et al. *JAMA Network Open*.

Characteristics	Telehealth visits only (n=220)	In-person & telehealth visits (n=2534)	In-person visits only (n=1764)
	**aOR (95% CI)**	**aOR (95% CI)**	**aOR (95% CI)**
Motivated to engage with digital services	0.64 (0.37, 1.10)	1.09 (0.79, 1.49)	0.73 (0.53, 1.00)
Access to digital services that work	0.65 (0.38, 1.13)	**1.62 (1.19, 2.21)**	**1.41 (1.04, 1.92)**
Digital services that suit individual needs	**2.11 (1.33, 3.34)**	1.11 (0.85, 1.44)	0.95 (0.72, 1.24)
**MHSVI**			
Most vulnerable counties	0.91 (0.65, 1.27)	1.14 (0.94, 1.38)	1.04 (0.86, 1.26)
Least vulnerable counties	Ref	Ref	Ref

Abbreviations: MHSVI= Minority Health Social Vulnerability Index, aOR= adjusted odds ratio, CI= confidence interval, Ref= reference group, AIAN= American Indian or Alaska Native, NHPI= Native Hawaiian or Pacific Islander, AA= African American.

Reference group for multinomial logit model was those who had no healthcare visits (*n*=926). Bolded cells represent significant results where the confidence interval does not include the null value, 1. Age and digital health literacy domains were entered as continuous variables, income and education were entered as ordinal variables, whereas all other variables (e.g., sex) were entered as categorical variables.

[a] Based on imputed data.

© 2026 Tesfaye L et al. *JAMA Network Open*.

eTable 3. Associations between individual themes of the Minority Health Social Vulnerability Index and optimal patient-centered communication

Theme	Give you the chance to ask questions	Give attention to your feelings	Involve you in decisions	Make sure you understand	Explain things	Spend enough time	Help deal with uncertainty
	aOR (95% CI)	aOR (95% CI)	aOR (95% CI)	aOR (95% CI)	aOR (95% CI)	aOR (95% CI)	aOR (95% CI)
Socioeconomic status	0.66 (0.34, 1.29)	1.32 (0.68, 2.57)	0.77 (0.40, 1.51)	0.88 (0.46, 1.71)	**0.49 (0.25, 0.95)**	0.97 (0.49, 1.92)	0.78 (0.40, 1.54)
Household Composition & Disability	1.29 (0.41, 4.02)	0.42 (0.13, 1.33)	1.25 (0.40, 3.87)	1.00 (0.32, 3.07)	1.03 (0.33, 3.17)	0.67 (0.21, 2.13)	1.46 (0.46, 4.58)
Minority Status & Language	1.39 (0.82, 2.35)	0.77 (0.45, 1.30)	0.81 (0.48, 1.36)	0.93 (0.55, 1.56)	1.40 (0.83, 2.36)	0.69 (0.40, 1.18)	0.93 (0.55, 1.58)
Housing Type & Transportation	1.02 (0.37, 2.76)	1.82 (0.66, 4.97)	1.15 (0.42, 3.09)	1.11 (0.41, 2.97)	1.37 (0.51, 3.69)	0.96 (0.35, 2.65)	1.40 (0.51, 3.86)
Health Care Infrastructure & Access	1.24 (0.53, 2.88)	1.29 (0.55, 3.01)	1.34 (0.57, 3.11)	1.44 (0.62, 3.33)	1.08 (0.47, 2.52)	0.95 (0.40, 2.24)	0.93 (0.39, 2.18)
Medical Vulnerability	1.39 (0.68, 2.83)	1.00 (0.49, 2.03)	1.01 (0.50, 2.05)	0.85 (0.42, 1.72)	1.82 (0.90, 3.68)	1.25 (0.60, 2.58)	1.23 (0.60, 2.50)

© 2026 Tesfaye L et al. *JAMA Network Open*.

eTable 4. Factors associated with optimal patient-centered communication among those who had 1 or more telehealth visits in the past year, N = 2714, sensitivity analysis with self-identifying Asian participants as a separate racial/ethnic group

Characteristics	PCC1	PCC2	PCC3	PCC4	PCC5	PCC6	PCC7
	aOR (95% CI)	aOR (95% CI)	aOR (95% CI)	aOR (95% CI)	aOR (95% CI)	aOR (95% CI)	aOR (95% CI)
**Age**	1.01 (0.99, 1.01)	1.00 (0.99, 1.01)	1.01 (0.99, 1.01)	1.01 (1.00, 1.02)	1.01 (1.00, 1.02)	**1.01 (1.01, 1.02)**	1.00 (0.99, 1.01)
**Sex**							
Female	1.15 (0.96, 1.37)	**1.34 (1.12, 1.60)**	**1.23 (1.03, 1.47)**	1.16 (0.97, 1.38)	**1.24 (1.04, 1.48)**	**1.25 (1.04, 1.49)**	1.14 (0.95, 1.36)
Male	Ref	Ref	Ref	Ref	Ref	Ref	Ref
**Sexual orientation**							
Heterosexual	Ref	Ref	Ref	Ref	Ref	Ref	Ref
Gay, lesbian, bisexual	1.17 (0.90, 1.52)	1.05 (0.82, 1.36)	1.04 (0.80, 1.35)	1.05 (0.81, 1.35)	**1.41 (1.08, 1.85)**	1.22 (0.94, 1.58)	1.06 (0.82, 1.37)
**Race and/or ethnicity**							
Asian	0.66 (0.42, 1.03)	**0.49 (0.31, 0.78)**	**0.41 (0.25, 0.67)**	0.70 (0.45, 1.10)	**0.53 (0.34, 0.83)**	**0.50 (0.31, 0.83)**	**0.39 (0.24, 0.64)**
Black or AA	0.86 (0.67, 1.09)	0.91 (0.71, 1.16)	0.96 (0.75, 1.23)	1.04 (0.81, 1.32)	1.12 (0.88, 1.43)	**0.75 (0.58, 0.96)**	0.96 (0.75, 1.23)
Hispanic or Latino	1.09 (0.85, 1.39)	0.87 (0.69, 1.12)	1.01 (0.79, 1.28)	1.06 (0.83, 1.36)	0.92 (0.72, 1.17)	0.89 (0.70, 1.14)	1.05 (0.82, 1.33)
White	Ref	Ref	Ref	Ref	Ref	Ref	Ref
**Education**	0.94 (0.85, 1.05)	0.95 (0.85, 1.06)	**0.88 (0.79, 0.98)**	**0.88 (0.79, 0.98)**	0.93 (0.83, 1.03)	**0.89 (0.79, 0.99)**	**0.88 (0.79, 0.98)**
**Income**	0.88 (0.65, 1.21)	0.78 (0.56, 1.07)	**0.72 (0.53, 0.98)**	**0.68 (0.50, 0.93)**	**0.63 (0.46, 0.86)**	0.78 (0.56, 1.08)	**0.67 (0.49, 0.92)**
**English proficiency**							
Very well	Ref	Ref	Ref	Ref	Ref	Ref	Ref
Well, not well, not at all	**0.44 (0.32, 0.60)**	**0.65 (0.48, 0.89)**	**0.47 (0.34, 0.65)**	**0.39 (0.28, 0.53)**	**0.50 (0.36, 0.69)**	**0.58 (0.42, 0.80)**	**0.48 (0.34, 0.66)**
**Health insurance**							
Insured	Ref	Ref	Ref	Ref	Ref	Ref	Ref
Uninsured	0.88 (0.63, 1.24)	0.82 (0.59, 1.16)	0.81 (0.57, 1.14)	0.74 (0.52, 1.04)	0.88 (0.63, 1.24)	1.01 (0.72, 1.43)	**0.68 (0.48, 0.97)**
**General health**							
Excellent, very good, good	Ref	Ref	Ref	Ref	Ref	Ref	Ref

© 2026 Tesfaye L et al. *JAMA Network Open.*

Characteristics	PCC1 aOR (95% CI)	PCC2 aOR (95% CI)	PCC3 aOR (95% CI)	PCC4 aOR (95% CI)	PCC5 aOR (95% CI)	PCC6 aOR (95% CI)	PCC7 aOR (95% CI)
Fair, poor	1.03 (0.83, 1.28)	0.96 (0.77, 1.19)	1.06 (0.86, 1.32)	1.10 (0.89, 1.36)	1.01 (0.81, 1.25)	1.01 (0.81, 1.26)	0.92 (0.74, 1.15)
**Mental health**							
Excellent, very good, good	Ref	Ref	Ref	Ref	Ref	Ref	Ref
Fair, poor	0.82 (0.67, 1.01)	**0.79 (0.65, 0.97)**	0.82 (0.67, 1.01)	**0.77 (0.63, 0.95)**	**0.79 (0.65, 0.97)**	**0.67 (0.55, 0.83)**	**0.73 (0.60, 0.90)**
**Has a primary care clinician**							
Yes	Ref	Ref	Ref	Ref	Ref	Ref	Ref
No	**0.59 (0.50, 0.71)**	**0.63 (0.53, 0.76)**	**0.59 (0.50, 0.71)**	**0.56 (0.47, 0.67)**	**0.65 (0.54, 0.77)**	**0.65 (0.54, 0.78)**	**0.68 (0.57, 0.82)**
**Presence of underlying clinical conditions**							
Yes	**1.27 (1.05, 1.54)**	1.06 (0.87, 1.29)	1.12 (0.92, 1.36)	0.99 (0.82, 1.21)	1.14 (0.94, 1.39)	1.09 (0.89, 1.32)	1.04 (0.86, 1.27)
No	Ref	Ref	Ref	Ref	Ref	Ref	Ref
**Past year in-person visit(s) with healthcare clinician**							
0 times	Ref	Ref	Ref	Ref	Ref	Ref	Ref
1 or more times	1.25 (0.91, 1.71)	1.22 (0.89, 1.68)	1.20 (0.88, 1.64)	1.15 (0.85, 1.56)	1.08 (0.80, 1.47)	1.10 (0.80, 1.51)	1.12 (0.81, 1.54)
**History of COVID-19 infection**							
Yes	1.17 (0.97, 1.40)	**1.22 (1.02, 1.47)**	**1.33 (1.10, 1.59)**	1.16 (0.96, 1.39)	1.14 (0.95, 1.37)	1.20 (1.00, 1.45)	1.14 (0.95, 1.37)
No	Ref	Ref	Ref	Ref	Ref	Ref	Ref
**Body mass index**							
Healthy (18.5 to <25)	Ref	Ref	Ref	Ref	Ref	Ref	Ref
Unhealthy (<18.5 or ≥25)	1.15 (0.95, 1.41)	1.07 (0.87, 1.31)	1.04 (0.85, 1.28)	1.11 (0.91, 1.35)	1.07 (0.88, 1.29)	0.93 (0.76, 1.13)	1.05 (0.85, 1.29)
**Physical activity**							
Sufficient (≥150 minutes per week)	Ref	Ref	Ref	Ref	Ref	Ref	Ref
Insufficient (<150 minutes per week)	0.90 (0.75, 1.09)	1.06 (0.88, 1.28)	0.96 (0.79, 1.15)	0.90 (0.75, 1.09)	1.09 (0.90, 1.31)	1.02 (0.84, 1.24)	0.99 (0.82, 1.20)

© 2026 Tesfaye L et al. *JAMA Network Open*.

Characteristics	PCC1 aOR (95% CI)	PCC2 aOR (95% CI)	PCC3 aOR (95% CI)	PCC4 aOR (95% CI)	PCC5 aOR (95% CI)	PCC6 aOR (95% CI)	PCC7 aOR (95% CI)
**Past month cigarette smoking**							
Yes	0.84 (0.68, 1.04)	0.98 (0.79, 1.21)	0.92 (0.74, 1.14)	0.84 (0.68, 1.04)	0.95 (0.77, 1.17)	1.03 (0.83, 1.28)	0.89 (0.72, 1.10)
No	Ref	Ref	Ref	Ref	Ref	Ref	Ref
**Past month e-cigarette use**							
Yes	0.85 (0.67, 1.09)	0.80 (0.63, 1.03)	0.81 (0.63, 1.03)	**0.78 (0.61, 0.99)**	**0.78 (0.61, 0.99)**	0.84 (0.66, 1.08)	1.09 (0.85, 1.38)
No	Ref	Ref	Ref	Ref	Ref	Ref	Ref
**Alcohol misuse**							
Yes	1.06 (0.76, 1.47)	0.99 (0.71, 1.37)	1.17 (0.84, 1.64)	0.90 (0.65, 1.24)	0.96 (0.69, 1.33)	0.84 (0.59, 1.18)	0.88 (0.63, 1.25)
No	Ref	Ref	Ref	Ref	Ref	Ref	Ref
Not applicable (<21 years)	0.94 (0.62, 1.44)	1.28 (0.84, 1.96)	0.76 (0.49, 1.18)	0.90 (0.59, 1.38)	0.76 (0.49, 1.18)	1.02 (0.66, 1.58)	0.98 (0.64, 1.50)
**Past month marijuana or cannabis use**							
Yes	0.94 (0.76, 1.16)	0.89 (0.72, 1.11)	**0.77 (0.62, 0.95)**	0.88 (0.71, 1.09)	**0.77 (0.63, 0.96)**	0.82 (0.66, 1.02)	0.90 (0.73, 1.11)
No	Ref	Ref	Ref	Ref	Ref	Ref	Ref
**Device access**							
Yes	Ref	Ref	Ref	Ref	Ref	Ref	Ref
No	0.46 (0.16, 1.34)	0.42 (0.14, 1.30)	0.50 (0.17, 1.45)	0.87 (0.35, 2.15)	0.80 (0.32, 2.03)	0.59 (0.22, 1.60)	0.52 (0.17, 1.64)
**Internet access**							
Yes	Ref	Ref	Ref	Ref	Ref	Ref	Ref
No	1.07 (0.54, 2.15)	0.78 (0.36, 1.71)	1.00 (0.46, 2.20)	0.86 (0.41, 1.79)	1.16 (0.57, 2.36)	1.40 (0.68, 2.92)	1.53 (0.74, 3.16)
**Digital health literacy**							
Using technology to process health information	0.88 (0.65, 1.21)	0.78 (0.56, 1.07)	**0.72 (0.53, 0.98)**	**0.68 (0.50, 0.93)**	**0.63 (0.46, 0.86)**	0.78 (0.56, 1.08)	**0.67 (0.49, 0.92)**
Understanding of health concepts and language	1.28 (0.95, 1.71)	1.34 (0.99, 1.82)	1.18 (0.87, 1.59)	**1.49 (1.10, 2.00)**	**2.01 (1.49, 2.72)**	1.30 (0.96, 1.77)	**1.48 (1.09, 2.02)**
Ability to actively engage with digital services	0.94 (0.73, 1.22)	0.87 (0.67, 1.13)	**1.42 (1.09, 1.85)**	1.07 (0.83, 1.38)	1.22 (0.94, 1.59)	0.87 (0.66, 1.14)	0.83 (0.63, 1.08)

Characteristics	PCC1 aOR (95% CI)	PCC2 aOR (95% CI)	PCC3 aOR (95% CI)	PCC4 aOR (95% CI)	PCC5 aOR (95% CI)	PCC6 aOR (95% CI)	PCC7 aOR (95% CI)
Feel safe and in control	1.12 (0.91, 1.38)	**1.47 (1.19, 1.82)**	**1.35 (1.10, 1.65)**	**1.33 (1.09, 1.63)**	1.21 (0.99, 1.48)	**1.61 (1.29, 2.01)**	**1.45 (1.18, 1.80)**
Motivated to engage with digital services	1.05 (0.76, 1.45)	1.34 (0.97, 1.85)	1.15 (0.83, 1.60)	1.14 (0.84, 1.56)	1.18 (0.84, 1.65)	1.09 (0.78, 1.53)	1.38 (0.99, 1.93)
Access to digital services that work	**2.10 (1.53, 2.87)**	**1.62 (1.17, 2.25)**	**1.51 (1.09, 2.09)**	**1.48 (1.08, 2.03)**	**1.48 (1.07, 2.03)**	**1.50 (1.07, 2.10)**	**1.59 (1.13, 2.24)**
Digital services that suit individual needs	1.25 (0.96, 1.62)	**1.41 (1.08, 1.84)**	1.23 (0.94, 1.61)	**1.37 (1.06, 1.76)**	1.16 (0.89, 1.51)	**1.60 (1.22, 2.10)**	**1.37 (1.04, 1.79)**
**MHSVI**							
Most vulnerable counties	1.02 (0.86, 1.22)	1.12 (0.94, 1.35)	0.99 (0.83, 1.18)	0.93 (0.78, 1.11)	0.97 (0.81, 1.16)	0.94 (0.79, 1.13)	1.04 (0.87, 1.25)
Least vulnerable counties	Ref	Ref	Ref	Ref	Ref	Ref	Ref

Abbreviations: MHSVI= Minority Health Social Vulnerability Index, aOR= adjusted odds ratio, CI= confidence interval, Ref= reference group, AA= African American.
PCC1: Give you the chance to ask questions, PCC2: Give attention to your feelings, PCC3: Involve you in decisions, PCC4: Make sure you understand, PCC5: Explain things, PCC6: Spend enough time, PCC7: Help deal with uncertainty.
Logistic regression analysis modeled the probability of 1= always. Bolded cells represent significant results where the confidence interval does not include the null value, 1.
Due to poor convergence in the MHSVI stratified models with a five-level race and/or ethnicity variable, we present this sensitivity analysis using a four-level race and/or ethnicity variable with the other (non-Asian) participants excluded (i.e., American Indian, Alaska Native, Native Hawaiian, Pacific Islander participants).

© 2026 Tesfaye L et al. *JAMA Network Open*.

eTable 5. Factors associated with optimal patient-centered communication among those who had 1 or more telehealth visits in the past year and resided in MHSVI most vulnerable counties, n = 1471, sensitivity analysis with self-identifying Asian participants as a separate racial/ethnic group

Characteristics	PCC1	PCC2	PCC3	PCC4	PCC5	PCC6	PCC7
	aOR (95% CI)	aOR (95% CI)	aOR (95% CI)	aOR (95% CI)	aOR (95% CI)	aOR (95% CI)	aOR (95% CI)
**Age**	1.00 (0.99, 1.01)	1.00 (0.99, 1.01)	1.00 (0.99, 1.01)	1.00 (0.99, 1.01)	1.00 (0.99, 1.01)	1.01 (0.99, 1.02)	1.00 (0.99, 1.01)
**Sex**							
Female	1.03 (0.81, 1.31)	1.24 (0.97, 1.57)	1.07 (0.84, 1.36)	0.90 (0.71, 1.15)	1.14 (0.89, 1.46)	1.28 (0.99, 1.64)	1.11 (0.87, 1.42)
Male	Ref	Ref	Ref	Ref	Ref	Ref	Ref
**Sexual orientation**							
Heterosexual	Ref	Ref	Ref	Ref	Ref	Ref	Ref
Gay, lesbian, bisexual	1.23 (0.86, 1.76)	0.99 (0.69, 1.40)	0.99 (0.69, 1.42)	1.22 (0.85, 1.74)	**1.47 (1.01, 2.12)**	1.17 (0.82, 1.69)	0.96 (0.68, 1.37)
**Race and/or ethnicity**							
Asian	1.29 (0.65, 2.57)	0.74 (0.37, 1.45)	**0.46 (0.23, 0.92)**	0.99 (0.50, 1.97)	0.89 (0.44, 1.80)	1.12 (0.57, 2.20)	0.59 (0.30, 1.18)
Black or AA	0.81 (0.60, 1.07)	0.89 (0.67, 1.19)	0.90 (0.67, 1.20)	0.94 (0.70, 1.25)	0.96 (0.72, 1.28)	**0.74 (0.55, 0.99)**	0.86 (0.64, 1.15)
Hispanic or Latino	1.07 (0.79, 1.44)	0.84 (0.62, 1.12)	0.99 (0.74, 1.32)	1.01 (0.75, 1.36)	0.85 (0.63, 1.15)	0.89 (0.66, 1.20)	1.06 (0.79, 1.42)
White	Ref	Ref	Ref	Ref	Ref	Ref	Ref
**Education**	**0.86 (0.75, 0.99)**	0.89 (0.77, 1.02)	**0.86 (0.75, 0.99)**	**0.86 (0.74, 0.99)**	0.91 (0.79, 1.05)	0.87 (0.75, 1.00)	**0.86 (0.75, 0.99)**
**Income**	0.94 (0.83, 1.06)	0.98 (0.87, 1.10)	1.02 (0.90, 1.15)	0.95 (0.84, 1.07)	0.99 (0.88, 1.12)	0.99 (0.87, 1.12)	0.92 (0.82, 1.04)
**English proficiency**							
Very well	Ref	Ref	Ref	Ref	Ref	Ref	Ref
Well, not well, not at all	**0.53 (0.36, 0.79)**	0.74 (0.51, 1.09)	**0.59 (0.40, 0.87)**	**0.40 (0.27, 0.60)**	**0.63 (0.42, 0.95)**	**0.64 (0.42, 0.95)**	**0.52 (0.36, 0.77)**
**Health insurance**							
Insured	Ref	Ref	Ref	Ref	Ref	Ref	Ref
Uninsured	0.89 (0.60, 1.31)	0.84 (0.57, 1.24)	0.79 (0.53, 1.16)	0.73 (0.50, 1.08)	0.89 (0.60, 1.31)	1.13 (0.77, 1.67)	0.69 (0.46, 1.02)
**General health**							
Excellent, very good, good	Ref	Ref	Ref	Ref	Ref	Ref	Ref

© 2026 Tesfaye L et al. *JAMA Network Open.*

Characteristics	PCC1<br>aOR (95% CI)	PCC2<br>aOR (95% CI)	PCC3<br>aOR (95% CI)	PCC4<br>aOR (95% CI)	PCC5<br>aOR (95% CI)	PCC6<br>aOR (95% CI)	PCC7<br>aOR (95% CI)
Fair, poor	0.88 (0.65, 1.18)	0.91 (0.67, 1.22)	0.90 (0.67, 1.21)	0.90 (0.67, 1.21)	0.91 (0.67, 1.22)	0.83 (0.61, 1.12)	0.80 (0.59, 1.07)
**Mental health**							
Excellent, very good, good	Ref	Ref	Ref	Ref	Ref	Ref	Ref
Fair, poor	0.86 (0.65, 1.14)	0.84 (0.64, 1.10)	0.90 (0.69, 1.19)	0.82 (0.62, 1.08)	0.77 (0.58, 1.01)	0.84 (0.63, 1.12)	0.88 (0.67, 1.16)
**Has a primary care clinician**							
Yes	Ref	Ref	Ref	Ref	Ref	Ref	Ref
No	**0.58 (0.46, 0.74)**	**0.66 (0.52, 0.84)**	**0.61 (0.48, 0.78)**	**0.62 (0.48, 0.79)**	**0.65 (0.51, 0.83)**	**0.63 (0.49, 0.81)**	**0.71 (0.56, 0.91)**
**Presence of underlying clinical conditions**							
Yes	**1.35 (1.03, 1.77)**	1.02 (0.78, 1.35)	1.24 (0.94, 1.62)	1.08 (0.82, 1.43)	**1.35 (1.02, 1.78)**	1.19 (0.90, 1.58)	0.98 (0.74, 1.29)
No	Ref	Ref	Ref	Ref	Ref	Ref	Ref
**Past year in-person visit(s) with healthcare clinician**							
0 times	Ref	Ref	Ref	Ref	Ref	Ref	Ref
1 or more times	0.95 (0.62, 1.46)	1.06 (0.69, 1.63)	0.99 (0.65, 1.52)	0.95 (0.62, 1.44)	0.96 (0.63, 1.46)	1.17 (0.74, 1.84)	1.12 (0.72, 1.74)
**History of COVID-19 infection**							
Yes	1.20 (0.94, 1.53)	1.19 (0.93, 1.52)	1.14 (0.89, 1.45)	1.14 (0.89, 1.46)	1.14 (0.89, 1.47)	1.10 (0.85, 1.41)	1.09 (0.86, 1.40)
No	Ref	Ref	Ref	Ref	Ref	Ref	Ref
**Body mass index**							
Healthy (18.5 to <25)	Ref	Ref	Ref	Ref	Ref	Ref	Ref
Unhealthy (<18.5 or ≥25)	1.23 (0.94, 1.62)	1.08 (0.82, 1.43)	1.06 (0.81, 1.41)	1.19 (0.90, 1.58)	1.18 (0.90, 1.56)	1.01 (0.76, 1.34)	1.17 (0.89, 1.55)
**Physical activity**							
Sufficient (≥150 minutes per week)	Ref	Ref	Ref	Ref	Ref	Ref	Ref
Insufficient (<150 minutes per week)	0.98 (0.75, 1.27)	1.05 (0.81, 1.37)	1.03 (0.80, 1.34)	0.89 (0.69, 1.16)	1.03 (0.79, 1.34)	1.05 (0.80, 1.37)	1.07 (0.82, 1.39)

© 2026 Tesfaye L et al. *JAMA Network Open*.

Characteristics	PCC1 aOR (95% CI)	PCC2 aOR (95% CI)	PCC3 aOR (95% CI)	PCC4 aOR (95% CI)	PCC5 aOR (95% CI)	PCC6 aOR (95% CI)	PCC7 aOR (95% CI)
**Past month cigarette smoking**							
Yes	0.81 (0.62, 1.07)	0.83 (0.63, 1.10)	0.79 (0.60, 1.04)	**0.70 (0.53, 0.92)**	0.90 (0.68, 1.19)	1.12 (0.84, 1.49)	0.77 (0.58, 1.02)
No	Ref	Ref	Ref	Ref	Ref	Ref	Ref
**Past month e-cigarette use**							
Yes	0.93 (0.68, 1.28)	0.78 (0.57, 1.08)	0.74 (0.54, 1.01)	0.83 (0.60, 1.14)	**0.69 (0.50, 0.95)**	0.80 (0.58, 1.12)	1.04 (0.76, 1.44)
No	Ref	Ref	Ref	Ref	Ref	Ref	Ref
**Alcohol misuse**							
Yes	1.36 (0.87, 2.13)	1.31 (0.82, 2.09)	1.14 (0.72, 1.79)	0.89 (0.57, 1.40)	1.07 (0.67, 1.72)	0.68 (0.41, 1.12)	0.87 (0.54, 1.40)
No	Ref	Ref	Ref	Ref	Ref	Ref	Ref
Not applicable (<21 years)	0.72 (0.41, 1.26)	1.04 (0.61, 1.77)	0.68 (0.38, 1.22)	0.65 (0.37, 1.13)	**0.56 (0.32, 0.99)**	0.85 (0.49, 1.47)	0.74 (0.43, 1.27)
**Past month marijuana or cannabis use**							
Yes	1.03 (0.78, 1.36)	0.99 (0.74, 1.31)	0.88 (0.66, 1.16)	0.85 (0.64, 1.13)	0.85 (0.64, 1.13)	0.82 (0.61, 1.10)	1.03 (0.78, 1.37)
No	Ref	Ref	Ref	Ref	Ref	Ref	Ref
**Device access**							
Yes	Ref	Ref	Ref	Ref	Ref	Ref	Ref
No	0.43 (0.12, 1.51)	0.25 (0.06, 1.06)	0.44 (0.13, 1.52)	0.92 (0.32, 2.64)	0.86 (0.30, 2.48)	0.62 (0.19, 1.95)	0.68 (0.21, 2.23)
**Internet access**							
Yes	Ref	Ref	Ref	Ref	Ref	Ref	Ref
No	1.14 (0.51, 2.58)	0.75 (0.29, 1.90)	1.13 (0.44, 2.87)	0.70 (0.30, 1.61)	1.10 (0.45, 2.68)	0.84 (0.32, 2.21)	1.49 (0.64, 3.46)
**Digital health literacy**							
Using technology to process health information	0.88 (0.58, 1.33)	0.75 (0.49, 1.16)	0.79 (0.53, 1.20)	0.81 (0.54, 1.23)	0.86 (0.57, 1.30)	0.78 (0.51, 1.21)	0.80 (0.52, 1.22)
Understanding of health concepts and language	1.36 (0.90, 2.05)	1.23 (0.81, 1.87)	1.10 (0.72, 1.67)	1.37 (0.90, 2.06)	**1.98 (1.30, 3.02)**	1.43 (0.94, 2.17)	1.37 (0.90, 2.09)
Ability to actively engage with digital services	0.83 (0.58, 1.17)	0.77 (0.54, 1.10)	1.41 (0.99, 2.02)	0.86 (0.61, 1.23)	1.02 (0.71, 1.45)	0.70 (0.49, 1.01)	0.77 (0.54, 1.10)

© 2026 Tesfaye L et al. *JAMA Network Open*.

Characteristics	PCC1 aOR (95% CI)	PCC2 aOR (95% CI)	PCC3 aOR (95% CI)	PCC4 aOR (95% CI)	PCC5 aOR (95% CI)	PCC6 aOR (95% CI)	PCC7 aOR (95% CI)
Feel safe and in control	0.92 (0.68, 1.23)	**1.57 (1.16, 2.11)**	**1.39 (1.05, 1.85)**	1.31 (0.99, 1.75)	1.33 (1.00, 1.77)	**1.55 (1.14, 2.11)**	**1.52 (1.12, 2.05)**
Motivated to engage with digital services	1.14 (0.74, 1.76)	1.34 (0.86, 2.09)	1.04 (0.66, 1.62)	1.07 (0.69, 1.66)	1.05 (0.66, 1.66)	0.97 (0.62, 1.52)	1.20 (0.76, 1.90)
Access to digital services that work	**2.16 (1.41, 3.31)**	1.45 (0.93, 2.28)	1.35 (0.86, 2.13)	**1.72 (1.11, 2.67)**	1.30 (0.84, 2.01)	1.57 (1.00, 2.48)	1.32 (0.82, 2.11)
Digital services that suit individual needs	1.29 (0.92, 1.80)	**1.63 (1.15, 2.32)**	1.29 (0.91, 1.81)	1.38 (0.99, 1.91)	1.13 (0.80, 1.60)	**1.87 (1.32, 2.64)**	**1.58 (1.11, 2.24)**

Abbreviations: MHSVI= Minority Health Social Vulnerability Index, aOR= adjusted odds ratio, CI= confidence interval, Ref= reference group, AA= African American.
PCC1: Give you the chance to ask questions, PCC2: Give attention to your feelings, PCC3: Involve you in decisions, PCC4: Make sure you understand, PCC5: Explain things, PCC6: Spend enough time, PCC7: Help deal with uncertainty.
Logistic regression analysis modeled the probability of 1= always. Bolded cells represent significant results where the confidence interval does not include the null value, 1.
Due to poor convergence in the MHSVI stratified models with a five-level race and/or ethnicity variable, we present this sensitivity analysis using a four-level race and/or ethnicity variable with the other (non-Asian) participants excluded (i.e., American Indian, Alaska Native, Native Hawaiian, Pacific Islander participants).

© 2026 Tesfaye L et al. *JAMA Network Open*.

eTable 6. Factors associated with optimal patient-centered communication among those who had 1 or more telehealth visits in the past year and resided in MHSVI least vulnerable counties, n = 1243, sensitivity analysis with self-identifying Asian participants as a separate racial/ethnic group

Characteristics	PCC1 aOR (95% CI)	PCC2 aOR (95% CI)	PCC3 aOR (95% CI)	PCC4 aOR (95% CI)	PCC5 aOR (95% CI)	PCC6 aOR (95% CI)	PCC7 aOR (95% CI)
**Age**	1.01 (0.99, 1.02)	1.01 (0.99, 1.02)	1.01 (1.00, 1.02)	**1.02 (1.01, 1.03)**	**1.02 (1.01, 1.03)**	**1.01 (1.01, 1.02)**	1.00 (0.99, 1.01)
**Sex**							
Female	**1.32 (1.01, 1.73)**	**1.50 (1.15, 1.97)**	**1.51 (1.15, 1.97)**	**1.61 (1.23, 2.12)**	**1.41 (1.08, 1.84)**	1.25 (0.96, 1.64)	1.19 (0.90, 1.56)
Male	Ref	Ref	Ref	Ref	Ref	Ref	Ref
**Sexual orientation**							
Heterosexual	Ref	Ref	Ref	Ref	Ref	Ref	Ref
Gay, lesbian, bisexual	1.15 (0.78, 1.70)	1.16 (0.79, 1.70)	1.13 (0.77, 1.67)	0.88 (0.60, 1.29)	1.48 (0.99, 2.20)	1.27 (0.86, 1.86)	1.24 (0.83, 1.83)
**Race and/or ethnicity**							
Asian	**0.40 (0.21, 0.74)**	**0.35 (0.18, 0.68)**	**0.37 (0.18, 0.74)**	**0.53 (0.28, 0.99)**	**0.37 (0.20, 0.68)**	**0.25 (0.12, 0.53)**	**0.25 (0.12, 0.50)**
Black or AA	1.13 (0.70, 1.84)	0.94 (0.57, 1.57)	1.17 (0.73, 1.89)	1.50 (0.93, 2.42)	**1.74 (1.03, 2.93)**	0.82 (0.50, 1.35)	1.39 (0.84, 2.29)
Hispanic or Latino	1.13 (0.71, 1.79)	0.90 (0.57, 1.43)	1.02 (0.65, 1.60)	1.07 (0.68, 1.69)	0.93 (0.60, 1.45)	0.88 (0.56, 1.38)	0.88 (0.55, 1.38)
White	Ref	Ref	Ref	Ref	Ref	Ref	Ref
**Education**	1.04 (0.87, 1.24)	1.03 (0.86, 1.22)	0.90 (0.76, 1.07)	0.89 (0.74, 1.06)	0.95 (0.80, 1.13)	0.90 (0.76, 1.08)	0.91 (0.76, 1.09)
**Income**	0.99 (0.87, 1.13)	0.97 (0.85, 1.12)	0.97 (0.84, 1.11)	1.02 (0.89, 1.17)	1.02 (0.90, 1.17)	0.99 (0.86, 1.13)	0.95 (0.83, 1.09)
**English proficiency**							
Very well	Ref	Ref	Ref	Ref	Ref	Ref	Ref
Well, not well, not at all	**0.27 (0.15, 0.49)**	**0.47 (0.27, 0.83)**	**0.29 (0.16, 0.53)**	**0.34 (0.20, 0.59)**	**0.31 (0.18, 0.55)**	**0.46 (0.26, 0.82)**	**0.37 (0.21, 0.67)**
**Health insurance**							
Insured	Ref	Ref	Ref	Ref	Ref	Ref	Ref
Uninsured	0.80 (0.41, 1.56)	0.74 (0.36, 1.50)	0.94 (0.46, 1.94)	0.66 (0.30, 1.45)	0.83 (0.41, 1.68)	0.68 (0.31, 1.51)	0.60 (0.27, 1.36)
**General health**							
Excellent, very good, good	Ref	Ref	Ref	Ref	Ref	Ref	Ref

© 2026 Tesfaye L et al. *JAMA Network Open*.

Characteristics	PCC1<br>aOR (95% CI)	PCC2<br>aOR (95% CI)	PCC3<br>aOR (95% CI)	PCC4<br>aOR (95% CI)	PCC5<br>aOR (95% CI)	PCC6<br>aOR (95% CI)	PCC7<br>aOR (95% CI)
Fair, poor	1.24 (0.90, 1.71)	1.02 (0.73, 1.43)	1.32 (0.95, 1.84)	**1.43 (1.03, 1.99)**	1.17 (0.84, 1.62)	1.28 (0.91, 1.80)	1.12 (0.80, 1.57)
**Mental health**							
Excellent, very good, good	Ref	Ref	Ref	Ref	Ref	Ref	Ref
Fair, poor	0.77 (0.56, 1.05)	**0.72 (0.53, 0.99)**	**0.71 (0.52, 0.97)**	**0.72 (0.53, 0.98)**	0.81 (0.59, 1.11)	**0.51 (0.37, 0.70)**	**0.55 (0.40, 0.77)**
**Has a primary care clinician**							
Yes	Ref	Ref	Ref	Ref	Ref	Ref	Ref
No	**0.61 (0.47, 0.81)**	**0.60 (0.46, 0.80)**	**0.57 (0.43, 0.75)**	**0.50 (0.38, 0.65)**	**0.63 (0.48, 0.83)**	**0.64 (0.48, 0.84)**	**0.61 (0.46, 0.81)**
**Presence of underlying clinical conditions**							
Yes	1.21 (0.91, 1.60)	1.11 (0.84, 1.48)	0.99 (0.74, 1.32)	0.90 (0.68, 1.19)	0.93 (0.70, 1.24)	0.99 (0.75, 1.32)	1.11 (0.83, 1.48)
No	Ref	Ref	Ref	Ref	Ref	Ref	Ref
**Past year in-person visit(s) with healthcare clinician**							
0 times	Ref	Ref	Ref	Ref	Ref	Ref	Ref
1 or more times	**1.63 (1.02, 2.61)**	1.41 (0.88, 2.24)	1.45 (0.91, 2.31)	1.35 (0.86, 2.12)	1.19 (0.76, 1.85)	1.04 (0.65, 1.65)	1.10 (0.68, 1.76)
**History of COVID-19 infection**							
Yes	1.12 (0.84, 1.49)	1.26 (0.95, 1.68)	**1.60 (1.20, 2.12)**	1.20 (0.90, 1.59)	1.12 (0.85, 1.48)	1.28 (0.97, 1.69)	1.16 (0.88, 1.54)
No	Ref	Ref	Ref	Ref	Ref	Ref	Ref
**Body mass index**							
Healthy (18.5 to <25)	Ref	Ref	Ref	Ref	Ref	Ref	Ref
Unhealthy (<18.5 or ≥25)	1.10 (0.81, 1.48)	1.04 (0.77, 1.41)	1.03 (0.76, 1.39)	1.02 (0.76, 1.38)	0.94 (0.70, 1.25)	0.82 (0.61, 1.12)	0.92 (0.67, 1.25)
**Physical activity**							
Sufficient (≥150 minutes per week)	Ref	Ref	Ref	Ref	Ref	Ref	Ref
Insufficient (<150 minutes per week)	0.81 (0.61, 1.07)	1.07 (0.81, 1.41)	0.89 (0.67, 1.18)	0.90 (0.68, 1.19)	1.14 (0.87, 1.51)	0.98 (0.74, 1.29)	0.90 (0.67, 1.20)

© 2026 Tesfaye L et al. *JAMA Network Open*.

Characteristics	PCC1 aOR (95% CI)	PCC2 aOR (95% CI)	PCC3 aOR (95% CI)	PCC4 aOR (95% CI)	PCC5 aOR (95% CI)	PCC6 aOR (95% CI)	PCC7 aOR (95% CI)
**Past month cigarette smoking**							
Yes	0.94 (0.67, 1.33)	1.28 (0.90, 1.81)	1.22 (0.86, 1.73)	1.15 (0.82, 1.63)	1.10 (0.78, 1.54)	1.01 (0.71, 1.42)	1.23 (0.87, 1.73)
No	Ref	Ref	Ref	Ref	Ref	Ref	Ref
**Past month e-cigarette use**							
Yes	0.75 (0.50, 1.11)	0.80 (0.54, 1.18)	0.91 (0.61, 1.34)	0.74 (0.50, 1.09)	0.95 (0.64, 1.40)	0.91 (0.61, 1.35)	1.16 (0.79, 1.70)
No	Ref	Ref	Ref	Ref	Ref	Ref	Ref
**Alcohol misuse**							
Yes	0.86 (0.52, 1.44)	0.76 (0.46, 1.27)	1.27 (0.76, 2.12)	0.96 (0.58, 1.57)	0.85 (0.53, 1.36)	1.02 (0.62, 1.68)	0.88 (0.53, 1.48)
No	Ref	Ref	Ref	Ref	Ref	Ref	Ref
Not applicable (<21 years)	1.31 (0.67, 2.59)	1.70 (0.84, 3.43)	0.85 (0.42, 1.71)	1.31 (0.67, 2.58)	1.04 (0.50, 2.17)	1.20 (0.58, 2.50)	1.37 (0.65, 2.93)
**Past month marijuana or cannabis use**							
Yes	0.84 (0.60, 1.16)	0.77 (0.55, 1.08)	**0.63 (0.46, 0.89)**	0.89 (0.64, 1.23)	**0.69 (0.50, 0.96)**	0.83 (0.59, 1.17)	0.75 (0.54, 1.04)
No	Ref	Ref	Ref	Ref	Ref	Ref	Ref
**Device access**							
Yes	Ref	Ref	Ref	Ref	Ref	Ref	Ref
No	0.52 (0.06, 4.59)	1.15 (0.20, 6.68)	0.58 (0.06, 5.75)	0.65 (0.10, 4.34)	0.62 (0.09, 4.36)	0.47 (0.05, 4.21)	0.12 (0.00, 5.45)
**Internet access**							
Yes	Ref	Ref	Ref	Ref	Ref	Ref	Ref
No	0.96 (0.22, 4.25)	0.91 (0.23, 3.60)	0.74 (0.15, 3.55)	1.26 (0.28, 5.67)	1.23 (0.35, 4.36)	3.56 (1.02, 12.40)	1.99 (0.43, 9.19)
**Digital health literacy**							
Using technology to process health information	0.94 (0.58, 1.52)	0.78 (0.47, 1.29)	**0.62 (0.39, 0.99)**	**0.54 (0.34, 0.85)**	**0.41 (0.25, 0.67)**	0.77 (0.47, 1.26)	**0.52 (0.32, 0.85)**
Understanding of health concepts and language	1.21 (0.78, 1.88)	1.49 (0.94, 2.37)	1.32 (0.85, 2.04)	**1.72 (1.10, 2.68)**	**2.15 (1.37, 3.39)**	1.18 (0.73, 1.90)	**1.64 (1.04, 2.61)**
Ability to actively engage with digital services	1.09 (0.73, 1.63)	1.01 (0.67, 1.51)	1.48 (0.99, 2.21)	1.46 (0.99, 2.16)	**1.52 (1.02, 2.27)**	1.15 (0.76, 1.75)	0.93 (0.61, 1.41)

© 2026 Tesfaye L et al. *JAMA Network Open*.

**Characteristics**	**PCC1**	**PCC2**	**PCC3**	**PCC4**	**PCC5**	**PCC6**	**PCC7**
	aOR (95% CI)	aOR (95% CI)	aOR (95% CI)	aOR (95% CI)	aOR (95% CI)	aOR (95% CI)	aOR (95% CI)
Feel safe and in control	**1.42 (1.05, 1.92)**	**1.45 (1.07, 1.97)**	1.34 (0.99, 1.79)	**1.42 (1.06, 1.90)**	1.10 (0.81, 1.50)	**1.75 (1.27, 2.43)**	**1.43 (1.04, 1.96)**
Motivated to engage with digital services	1.01 (0.61, 1.69)	1.41 (0.86, 2.33)	1.38 (0.85, 2.24)	1.28 (0.82, 2.01)	1.46 (0.89, 2.39)	1.22 (0.73, 2.04)	**1.84 (1.12, 3.03)**
Access to digital services that work	**2.08 (1.30, 3.35)**	**1.94 (1.19, 3.16)**	**1.78 (1.10, 2.87)**	1.25 (0.78, 2.00)	**1.62 (1.01, 2.61)**	1.50 (0.90, 2.48)	**2.03 (1.22, 3.38)**
Digital services that suit individual needs	1.08 (0.72, 1.63)	1.07 (0.71, 1.61)	1.14 (0.75, 1.72)	1.25 (0.84, 1.86)	1.17 (0.77, 1.76)	1.25 (0.81, 1.93)	1.06 (0.70, 1.61)

Abbreviations: MHSVI= Minority Health Social Vulnerability Index, aOR= adjusted odds ratio, CI= confidence interval, Ref= reference group, AA= African American.
PCC1: Give you the chance to ask questions, PCC2: Give attention to your feelings, PCC3: Involve you in decisions, PCC4: Make sure you understand, PCC5: Explain things, PCC6: Spend enough time, PCC7: Help deal with uncertainty.
Logistic regression analysis modeled the probability of 1= always. Bolded cells represent significant results where the confidence interval does not include the null value, 1.
Due to poor convergence in the MHSVI stratified models with a five-level race and/or ethnicity variable, we present this sensitivity analysis using a four-level race and/or ethnicity variable with the other (non-Asian) participants excluded (i.e., American Indian, Alaska Native, Native Hawaiian, Pacific Islander participants).

© 2026 Tesfaye L et al. *JAMA Network Open*.

eTable 7. Factors associated with optimal patient-centered communication among those who had 1 or more telehealth visits in the past year, *N* = 2754, imputed data

Characteristics	Give chance to ask questions		Give attention to feelings		Involve you in decisions	
	PM (95%CI)	aOR (95% CI)	PM (95%CI)	aOR (95% CI)	PM (95%CI)	aOR (95% CI)
**Age**						
18	0.42 (0.38, 0.46)	1.00 (0.99, 1.01)	0.39 (0.35, 0.43)	1.00 (0.99, 1.01)	0.41 (0.37, 0.45)	1.00 (0.99, 1.01)
30	0.43 (0.41, 0.46)		0.40 (0.37, 0.43)		0.43 (0.40, 0.45)	
45	0.45 (0.43, 0.47)		0.41 (0.40, 0.43)		0.44 (0.43, 0.46)	
60	0.47 (0.44, 0.50)		0.43 (0.40, 0.46)		0.46 (0.44, 0.49)	
**Sex**						
Female	0.47 (0.44, 0.49)	1.17 (0.98, 1.40)	0.44 (0.42, 0.46)	**1.34 (1.12, 1.60)**	0.46 (0.44, 0.49)	**1.24 (1.04, 1.47)**
Male	0.43 (0.40, 0.46)	Ref	0.38 (0.35, 0.41)	Ref	0.42 (0.39, 0.45)	Ref
**Sexual orientation**						
Heterosexual	0.45 (0.43, 0.47)	Ref	0.41 (0.39, 0.43)	Ref	0.44 (0.42, 0.46)	Ref
Gay, lesbian, bisexual	0.48 (0.43, 0.53)	1.15 (0.89, 1.50)	0.42 (0.37, 0.47)	1.05 (0.81, 1.35)	0.45 (0.40, 0.50)	1.04 (0.80, 1.35)
**Race and/or ethnicity**						
AIAN, Asian, NHPI	0.38 (0.31, 0.46)	0.69 (0.48, 1.01)	0.29 (0.23, 0.37)	**0.50 (0.34, 0.75)**	0.32 (0.25, 0.40)	**0.52 (0.35, 0.77)**
Black or AA	0.42 (0.38, 0.47)	0.85 (0.66, 1.08)	0.41 (0.36, 0.45)	0.90 (0.70, 1.15)	0.44 (0.40, 0.49)	0.95 (0.75, 1.22)
Hispanic or Latino	0.48 (0.43, 0.52)	1.08 (0.85, 1.38)	0.40 (0.36, 0.44)	0.87 (0.68, 1.11)	0.45 (0.41, 0.50)	1.00 (0.79, 1.28)
White	0.46 (0.43, 0.48)	Ref	0.43 (0.40, 0.45)	Ref	0.45 (0.43, 0.48)	Ref
**Education**						
<High school	0.48 (0.43, 0.53)	0.92 (0.83, 1.03)	0.44 (0.39, 0.49)	0.93 (0.84, 1.04)	0.51 (0.46, 0.56)	**0.85 (0.76, 0.95)**
High school graduate	0.47 (0.44, 0.50)		0.43 (0.40, 0.45)		0.48 (0.45, 0.50)	
Vocational school, some college	0.45 (0.43, 0.47)		0.41 (0.39, 0.43)		0.44 (0.42, 0.46)	
College graduate or higher	0.43 (0.41, 0.47)		0.40 (0.37, 0.43)		0.41 (0.38, 0.44)	
**Income**						
<$20,000	0.46 (0.43, 0.50)	0.96 (0.88, 1.05)	0.42 (0.39, 0.45)	0.97 (0.89, 1.06)	0.44 (0.41, 0.48)	0.99 (0.91, 1.08)
$20,000 to $49,999	0.45 (0.43, 0.48)		0.42 (0.40, 0.44)		0.44 (0.42, 0.46)	
$50,000 to $74,999	0.45 (0.43, 0.47)		0.41 (0.39, 0.43)		0.44 (0.42, 0.46)	
≥$75,000	0.44 (0.41, 0.47)		0.41 (0.37, 0.44)		0.44 (0.41, 0.48)	
**English proficiency**						
Very well	0.47 (0.45, 0.49)	Ref	0.42 (0.40, 0.44)	Ref	0.46 (0.44, 0.48)	Ref

© 2026 Tesfaye L et al. *JAMA Network Open.*

Characteristics	Give chance to ask questions		Give attention to feelings		Involve you in decisions	
	PM (95%CI)	aOR (95% CI)	PM (95%CI)	aOR (95% CI)	PM (95%CI)	aOR (95% CI)
Well, not well, not at all	0.29 (0.24, 0.35)	**0.43 (0.31, 0.58)**	0.34 (0.28, 0.40)	**0.66 (0.48, 0.89)**	0.30 (0.25, 0.36)	**0.47 (0.34, 0.64)**
**Health insurance**						
Insured	0.45 (0.43, 0.47)	Ref	0.42 (0.40, 0.44)	Ref	0.45 (0.43, 0.47)	Ref
Uninsured	0.42 (0.35, 0.49)	0.85 (0.61, 1.19)	0.37 (0.31, 0.44)	0.80 (0.57, 1.13)	0.41 (0.34, 0.48)	0.82 (0.59, 1.16)
**General health**						
Excellent, very good, good	0.45 (0.43, 0.47)	Ref	0.42 (0.39, 0.44)	Ref	0.44 (0.42, 0.46)	Ref
Fair, poor	0.46 (0.42, 0.50)	1.03 (0.83, 1.28)	0.40 (0.37, 0.44)	0.94 (0.76, 1.17)	0.45 (0.41, 0.49)	1.04 (0.84, 1.29)
**Mental health**						
Excellent, very good, good	0.47 (0.44, 0.49)	Ref	0.43 (0.41, 0.45)	Ref	0.46 (0.43, 0.48)	Ref
Fair, poor	0.42 (0.38, 0.45)	**0.79 (0.64, 0.97)**	0.38 (0.34, 0.41)	**0.78 (0.64, 0.96)**	0.41 (0.38, 0.45)	0.81 (0.66, 1.00)
**Has a primary care clinician**						
Yes	0.50 (0.48, 0.53)	Ref	0.46 (0.43, 0.48)	Ref	0.49 (0.47, 0.52)	Ref
No	0.39 (0.36, 0.42)	**0.59 (0.50, 0.71)**	0.36 (0.33, 0.39)	**0.63 (0.53, 0.76)**	0.38 (0.35, 0.41)	**0.59 (0.50, 0.71)**
**Presence of underlying clinical conditions**						
Yes	0.48 (0.45, 0.51)	**1.26 (1.04, 1.53)**	0.42 (0.39, 0.45)	1.05 (0.86, 1.28)	0.46 (0.43, 0.49)	1.14 (0.94, 1.38)
No	0.43 (0.41, 0.46)	Ref	0.41 (0.38, 0.43)	Ref	0.43 (0.41, 0.46)	Ref
**Past year in-person visit(s) with healthcare clinician**						
0 times	0.41 (0.35, 0.48)	Ref	0.38 (0.32, 0.45)	Ref	0.41 (0.35, 0.48)	Ref
1 or more times	0.45 (0.44, 0.47)	1.20 (0.88, 1.65)	0.42 (0.40, 0.43)	1.18 (0.86, 1.62)	0.45 (0.43, 0.47)	1.17 (0.85, 1.59)
**History of COVID-19 infection**						
Yes	0.48 (0.44, 0.51)	1.17 (0.98, 1.40)	0.44 (0.41, 0.47)	**1.21 (1.01, 1.45)**	0.49 (0.45, 0.52)	**1.33 (1.11, 1.60)**
No	0.44 (0.42, 0.46)	Ref	0.40 (0.38, 0.42)	Ref	0.42 (0.40, 0.45)	Ref
**Body mass index**						
Healthy (18.5 to <25)	0.43 (0.39, 0.47)	Ref	0.40 (0.37, 0.44)	Ref	0.44 (0.40, 0.47)	Ref

© 2026 Tesfaye L et al. *JAMA Network Open*.

Characteristics	Give chance to ask questions		Give attention to feelings		Involve you in decisions	
	PM (95%CI)	aOR (95% CI)	PM (95%CI)	aOR (95% CI)	PM (95%CI)	aOR (95% CI)
Unhealthy (<18.5 or ≥25)	0.46 (0.44, 0.48)	1.14 (0.94, 1.39)	0.42 (0.39, 0.44)	1.05 (0.86, 1.29)	0.45 (0.42, 0.47)	1.03 (0.85, 1.26)
**Physical activity**						
Sufficient (≥150 minutes per week)	0.46 (0.44, 0.48)	Ref	0.41 (0.39, 0.43)	Ref	0.45 (0.42, 0.47)	Ref
Insufficient (<150 minutes per week)	0.44 (0.40, 0.47)	0.90 (0.75, 1.09)	0.42 (0.39, 0.45)	1.06 (0.87, 1.28)	0.44 (0.41, 0.47)	0.96 (0.80, 1.16)
**Past month cigarette smoking**						
Yes	0.43 (0.40, 0.47)	0.86 (0.69, 1.06)	0.41 (0.37, 0.44)	0.97 (0.78, 1.20)	0.43 (0.40, 0.47)	0.91 (0.73, 1.13)
No	0.46 (0.44, 0.49)	Ref	0.42 (0.39, 0.44)	Ref	0.45 (0.43, 0.48)	Ref
**Past month e-cigarette use**						
Yes	0.42 (0.38, 0.47)	0.84 (0.66, 1.07)	0.38 (0.34, 0.42)	0.80 (0.63, 1.02)	0.41 (0.37, 0.45)	0.80 (0.63, 1.03)
No	0.46 (0.44, 0.48)	Ref	0.42 (0.40, 0.45)	Ref	0.45 (0.43, 0.48)	Ref
**Alcohol misuse**						
Yes	0.46 (0.39, 0.53)	1.04 (0.75, 1.45)	0.41 (0.34, 0.48)	0.97 (0.70, 1.36)	0.48 (0.41, 0.55)	1.18 (0.84, 1.66)
No	0.45 (0.43, 0.47)	Ref	0.41 (0.39, 0.43)	Ref	0.44 (0.42, 0.46)	Ref
Not applicable (<21 years)	0.44 (0.35, 0.52)	0.92 (0.60, 1.41)	0.46 (0.38, 0.55)	1.26 (0.83, 1.92)	0.38 (0.30, 0.47)	0.74 (0.48, 1.15)
**Past month marijuana or cannabis use**						
Yes	0.44 (0.41, 0.48)	0.94 (0.76, 1.15)	0.40 (0.36, 0.43)	0.89 (0.72, 1.10)	0.41 (0.37, 0.44)	**0.77 (0.63, 0.96)**
No	0.46 (0.43, 0.48)	Ref	0.42 (0.40, 0.44)	Ref	0.46 (0.44, 0.48)	Ref
**Device access**						
Yes	0.45 (0.43, 0.47)	Ref	0.41 (0.40, 0.43)	Ref	0.45 (0.43, 0.46)	Ref
No	0.30 (0.14, 0.52)	0.46 (0.16, 1.34)	0.25 (0.11, 0.48)	0.42 (0.13, 1.30)	0.29 (0.14, 0.52)	0.47 (0.16, 1.39)
**Internet access**						
Yes	0.45 (0.43, 0.47)	Ref	0.41 (0.40, 0.43)	Ref	0.44 (0.42, 0.46)	Ref
No	0.46 (0.32, 0.60)	1.01 (0.51, 2.00)	0.35 (0.22, 0.52)	0.74 (0.34, 1.60)	0.46 (0.30, 0.62)	1.06 (0.49, 2.32)

© 2026 Tesfaye L et al. *JAMA Network Open*.

Characteristics	Give chance to ask questions		Give attention to feelings		Involve you in decisions	
	PM (95%CI)	aOR (95% CI)	PM (95%CI)	aOR (95% CI)	PM (95%CI)	aOR (95% CI)
**Digital health literacy**						
Using technology to process health information	0.46 (0.43, 0.48)	0.89 (0.65, 1.21)	0.43 (0.40, 0.45)	0.76 (0.55, 1.05)	0.46 (0.44, 0.48)	**0.70 (0.51, 0.95)**
	0.44 (0.41, 0.47)		0.39 (0.37, 0.42)		0.42 (0.39, 0.45)	
Understanding of health concepts and language	0.44 (0.41, 0.46)	1.28 (0.95, 1.71)	0.39 (0.37, 0.42)	**1.37 (1.01, 1.85)**	0.43 (0.41, 0.46)	1.19 (0.88, 1.60)
	0.47 (0.44, 0.50)		0.43 (0.41, 0.46)		0.46 (0.43, 0.48)	
Ability to actively engage with digital services	0.46 (0.43, 0.48)	0.92 (0.72, 1.20)	0.42 (0.40, 0.44)	0.86 (0.66, 1.12)	0.42 (0.40, 0.45)	**1.42 (1.09, 1.84)**
	0.45 (0.42, 0.47)		0.40 (0.38, 0.43)		0.47 (0.44, 0.50)	
Feel safe and in control	0.44 (0.42, 0.47)	1.12 (0.91, 1.37)	0.38 (0.36, 0.41)	**1.49 (1.20, 1.83)**	0.42 (0.40, 0.45)	**1.36 (1.11, 1.66)**
	0.46 (0.44, 0.48)		0.44 (0.41, 0.46)		0.46 (0.44, 0.48)	
Motivated to engage with digital services	0.45 (0.43, 0.47)	1.02 (0.74, 1.41)	0.40 (0.38, 0.43)	1.28 (0.92, 1.77)	0.44 (0.41, 0.46)	1.14 (0.83, 1.58)
	0.45 (0.42, 0.49)		0.43 (0.40, 0.47)		0.46 (0.42, 0.49)	
Access to digital services that work	0.39 (0.36, 0.42)	**2.16 (1.58, 2.96)**	0.37 (0.34, 0.41)	**1.62 (1.17, 2.25)**	0.41 (0.38, 0.44)	**1.52 (1.10, 2.09)**
	0.51 (0.48, 0.53)		0.44 (0.42, 0.47)		0.47 (0.44, 0.50)	
Digital services that suit individual needs	0.43 (0.40, 0.46)	1.23 (0.95, 1.59)	0.38 (0.35, 0.41)	**1.43 (1.10, 1.87)**	0.42 (0.39, 0.46)	1.23 (0.95, 1.61)
	0.47 (0.44, 0.49)		0.44 (0.41, 0.46)		0.46 (0.43, 0.49)	
**MHSVI**						
Most vulnerable counties	0.45 (0.43, 0.48)	1.01 (0.84, 1.20)	0.42 (0.40, 0.45)	1.11 (0.93, 1.33)	0.44 (0.42, 0.47)	0.99 (0.82, 1.18)
Least vulnerable counties	0.45 (0.42, 0.48)	Ref	0.40 (0.37, 0.43)	Ref	0.44 (0.42, 0.47)	Ref

© 2026 Tesfaye L et al. *JAMA Network Open*.

eTable 7. Factors associated with optimal patient-centered communication among those who had 1 or more telehealth visits in the past year, *N* = 2754, imputed data (continued)

Characteristics	Make sure you understand		Explain things		Spend enough time		Help deal with uncertainty	
	PM (95%CI)	aOR (95% CI)	PM (95% CI)	aOR (95% CI)	PM (95% CI)	aOR (95% CI)	PM (95% CI)	aOR (95% CI)
**Age**								
18	0.45 (0.40, 0.49)	1.00 (1.00, 1.01)	0.44 (0.40, 0.48)	1.00 (1.00, 1.01)	0.32 (0.28, 0.36)	1.01 (1.00, 1.01)	0.37 (0.33, 0.41)	1.00 (0.99, 1.01)
30	0.47 (0.44, 0.49)		0.46 (0.43, 0.49)		0.35 (0.32, 0.37)		0.38 (0.35, 0.41)	
45	0.49 (0.48, 0.51)		0.49 (0.47, 0.51)		0.38 (0.37, 0.40)		0.39 (0.38, 0.41)	
60	0.52 (0.49, 0.55)		0.52 (0.50, 0.55)		0.42 (0.39, 0.45)		0.41 (0.38, 0.43)	
**Sex**								
Female	0.51 (0.48, 0.53)	1.18 (0.99, 1.40)	0.51 (0.49, 0.54)	**1.26 (1.05, 1.50)**	0.40 (0.38, 0.43)	**1.26 (1.06, 1.51)**	0.41 (0.38, 0.43)	1.15 (0.96, 1.37)
Male	0.47 (0.44, 0.50)	Ref	0.46 (0.44, 0.49)	Ref	0.36 (0.33, 0.38)	Ref	0.38 (0.35, 0.40)	Ref
**Sexual orientation**								
Heterosexual	0.49 (0.47, 0.51)	Ref	0.48 (0.46, 0.50)	Ref	0.38 (0.36, 0.40)	Ref	0.39 (0.37, 0.41)	Ref
Gay, lesbian, bisexual	0.50 (0.45, 0.55)	1.04 (0.80, 1.34)	0.55 (0.50, 0.61)	**1.39 (1.06, 1.83)**	0.42 (0.37, 0.47)	1.20 (0.93, 1.57)	0.40 (0.35, 0.45)	1.04 (0.80, 1.35)
**Race and/or ethnicity**								
AIAN, Asian, NHPI	0.45 (0.37, 0.53)	0.81 (0.56, 1.19)	0.40 (0.32, 0.47)	**0.62 (0.42, 0.90)**	0.30 (0.23, 0.37)	**0.58 (0.39, 0.88)**	0.26 (0.20, 0.34)	**0.50 (0.33, 0.74)**
Black or AA	0.50 (0.45, 0.54)	1.02 (0.80, 1.30)	0.52 (0.47, 0.57)	1.11 (0.87, 1.42)	0.34 (0.30, 0.39)	**0.74 (0.57, 0.95)**	0.39 (0.35, 0.44)	0.95 (0.74, 1.22)
Hispanic or Latino	0.50 (0.46, 0.55)	1.05 (0.82, 1.34)	0.48 (0.43, 0.52)	0.91 (0.71, 1.17)	0.38 (0.34, 0.42)	0.89 (0.69, 1.14)	0.41 (0.37, 0.45)	1.04 (0.82, 1.33)
White	0.49 (0.47, 0.52)	Ref	0.50 (0.47, 0.52)	Ref	0.40 (0.38, 0.43)	Ref	0.40 (0.38, 0.42)	Ref
**Education**								
<High school	0.55 (0.50, 0.60)	**0.86 (0.77, 0.96)**	0.53 (0.48, 0.58)	0.91 (0.82, 1.01)	0.44 (0.39, 0.49)	**0.87 (0.77, 0.97)**	0.45 (0.41, 0.50)	**0.86 (0.77, 0.96)**

© 2026 Tesfaye L et al. *JAMA Network Open*.

Characteristics	Make sure you understand		Explain things		Spend enough time		Help deal with uncertainty	
	PM (95%CI)	aOR (95% CI)	PM (95% CI)	aOR (95% CI)	PM (95% CI)	aOR (95% CI)	PM (95% CI)	aOR (95% CI)
High school graduate	0.52 (0.49, 0.55)		0.51 (0.48, 0.54)		0.41 (0.38, 0.44)		0.42 (0.39, 0.45)	
Vocational school, some college	0.49 (0.47, 0.51)		0.49 (0.47, 0.51)		0.38 (0.36, 0.40)		0.39 (0.37, 0.41)	
College graduate or higher	0.46 (0.43, 0.49)		0.47 (0.44, 0.50)		0.35 (0.33, 0.38)		0.36 (0.33, 0.39)	
**Income**								
<$20,000	0.50 (0.47, 0.53)	0.97 (0.89, 1.06)	0.49 (0.46, 0.52)	1.00 (0.92, 1.09)	0.39 (0.36, 0.42)	0.98 (0.89, 1.07)	0.41 (0.38, 0.45)	0.93 (0.85, 1.01)
$20,000 to $49,999	0.50 (0.48, 0.52)		0.49 (0.47, 0.51)		0.38 (0.36, 0.40)		0.40 (0.38, 0.42)	
$50,000 to $74,999	0.49 (0.47, 0.51)		0.49 (0.47, 0.51)		0.38 (0.36, 0.40)		0.38 (0.36, 0.41)	
≥$75,000	0.48 (0.45, 0.52)		0.49 (0.46, 0.53)		0.38 (0.34, 0.41)		0.37 (0.34, 0.40)	
**English proficiency**								
Very well	0.51 (0.49, 0.53)	Ref	0.51 (0.49, 0.53)	Ref	0.39 (0.37, 0.41)	Ref	0.41 (0.39, 0.43)	Ref
Well, not well, not at all	0.31 (0.26, 0.37)	**0.39 (0.28, 0.53)**	0.35 (0.29, 0.42)	**0.48 (0.35, 0.66)**	0.28 (0.23, 0.34)	**0.56 (0.40, 0.77)**	0.26 (0.21, 0.31)	**0.46 (0.33, 0.63)**
**Health insurance**								
Insured	0.50 (0.48, 0.52)	Ref	0.49 (0.47, 0.51)	Ref	0.38 (0.36, 0.40)	Ref	0.40 (0.38, 0.42)	Ref
Uninsured	0.44 (0.37, 0.51)	0.74 (0.53, 1.05)	0.47 (0.40, 0.54)	0.87 (0.62, 1.22)	0.38 (0.32, 0.45)	1.00 (0.71, 1.41)	0.32 (0.26, 0.39)	**0.68 (0.47, 0.96)**
**General health**								
Excellent, very good, good	0.49 (0.46, 0.51)	Ref	0.49 (0.47, 0.51)	Ref	0.38 (0.36, 0.40)	Ref	0.40 (0.38, 0.42)	Ref
Fair, poor	0.51 (0.47, 0.55)	1.11 (0.90, 1.38)	0.49 (0.45, 0.53)	1.00 (0.81, 1.24)	0.38 (0.35, 0.42)	1.00 (0.80, 1.25)	0.38 (0.34, 0.42)	0.90 (0.73, 1.12)
**Mental health**								
Excellent, very good, good	0.51 (0.49, 0.53)	Ref	0.51 (0.49, 0.53)	Ref	0.41 (0.39, 0.43)	Ref	0.41 (0.39, 0.44)	Ref
Fair, poor	0.45 (0.41, 0.49)	**0.75 (0.61, 0.91)**	0.45 (0.42, 0.49)	**0.77 (0.62, 0.94)**	0.32 (0.29, 0.36)	**0.65 (0.53, 0.81)**	0.34 (0.31, 0.38)	**0.71 (0.58, 0.88)**

© 2026 Tesfaye L et al. *JAMA Network Open*.

Characteristics	Make sure you understand		Explain things		Spend enough time		Help deal with uncertainty	
	PM (95%CI)	aOR (95% CI)	PM (95% CI)	aOR (95% CI)	PM (95% CI)	aOR (95% CI)	PM (95% CI)	aOR (95% CI)
**Has a primary care clinician**								
Yes	0.55 (0.52, 0.57)	Ref	0.53 (0.51, 0.56)	Ref	0.42 (0.40, 0.45)	Ref	0.43 (0.40, 0.45)	Ref
No	0.42 (0.40, 0.45)	**0.56 (0.47, 0.67)**	0.44 (0.41, 0.47)	**0.65 (0.54, 0.77)**	0.33 (0.31, 0.36)	**0.65 (0.54, 0.78)**	0.35 (0.32, 0.38)	**0.68 (0.57, 0.82)**
**Presence of underlying clinical conditions**								
Yes	0.49 (0.46, 0.52)	0.99 (0.82, 1.20)	0.51 (0.48, 0.54)	1.15 (0.94, 1.39)	0.39 (0.36, 0.42)	1.08 (0.89, 1.32)	0.40 (0.37, 0.43)	1.05 (0.86, 1.28)
No	0.49 (0.47, 0.52)	Ref	0.48 (0.46, 0.51)	Ref	0.38 (0.35, 0.40)	Ref	0.39 (0.36, 0.41)	Ref
**Past year in-person visit(s) with healthcare clinician**								
0 times	0.47 (0.41, 0.53)	Ref	0.48 (0.42, 0.54)	Ref	0.37 (0.31, 0.43)	Ref	0.37 (0.31, 0.44)	Ref
1 or more times	0.50 (0.48, 0.51)	1.12 (0.83, 1.52)	0.49 (0.47, 0.51)	1.06 (0.78, 1.44)	0.38 (0.37, 0.40)	1.09 (0.79, 1.50)	0.39 (0.38, 0.41)	1.11 (0.81, 1.53)
**History of COVID-19 infection**								
Yes	0.52 (0.48, 0.55)	1.15 (0.96, 1.39)	0.51 (0.48, 0.54)	1.14 (0.95, 1.36)	0.41 (0.38, 0.44)	1.20 (1.00, 1.44)	0.41 (0.38, 0.45)	1.14 (0.95, 1.36)
No	0.48 (0.46, 0.51)	Ref	0.48 (0.46, 0.51)	Ref	0.37 (0.35, 0.39)	Ref	0.38 (0.36, 0.41)	Ref
**Body mass index**								
Healthy (18.5 to <25)	0.48 (0.44, 0.51)	Ref	0.48 (0.45, 0.52)	Ref	0.39 (0.36, 0.43)	Ref	0.38 (0.35, 0.42)	Ref
Unhealthy (<18.5 or ≥25)	0.50 (0.48, 0.52)	1.10 (0.90, 1.34)	0.50 (0.47, 0.52)	1.06 (0.88, 1.29)	0.38 (0.36, 0.40)	0.93 (0.76, 1.13)	0.40 (0.37, 0.42)	1.05 (0.86, 1.30)
**Physical activity**								
Sufficient (≥150 minutes per week)	0.50 (0.48, 0.52)	Ref	0.49 (0.46, 0.51)	Ref	0.38 (0.36, 0.40)	Ref	0.39 (0.37, 0.42)	Ref

© 2026 Tesfaye L et al. *JAMA Network Open*.

Characteristics	Make sure you understand		Explain things		Spend enough time		Help deal with uncertainty	
	PM (95%CI)	aOR (95% CI)	PM (95% CI)	aOR (95% CI)	PM (95% CI)	aOR (95% CI)	PM (95% CI)	aOR (95% CI)
Insufficient (<150 minutes per week)	0.48 (0.45, 0.51)	0.91 (0.75, 1.09)	0.50 (0.47, 0.54)	1.08 (0.90, 1.31)	0.39 (0.36, 0.42)	1.03 (0.85, 1.25)	0.39 (0.36, 0.43)	1.00 (0.82, 1.20)
**Past month cigarette smoking**								
Yes	0.47 (0.43, 0.50)	0.84 (0.68, 1.03)	0.49 (0.45, 0.52)	0.95 (0.77, 1.17)	0.39 (0.36, 0.42)	1.04 (0.83, 1.28)	0.38 (0.34, 0.41)	0.89 (0.72, 1.10)
No	0.51 (0.48, 0.53)	Ref	0.50 (0.47, 0.52)	Ref	0.38 (0.36, 0.40)	Ref	0.40 (0.38, 0.43)	Ref
**Past month e-cigarette use**								
Yes	0.45 (0.41, 0.50)	**0.78 (0.61, 0.99**)	0.45 (0.41, 0.50)	**0.78 (0.61, 0.99)**	0.36 (0.31, 0.40)	0.84 (0.65, 1.07)	0.41 (0.36, 0.45)	1.08 (0.85, 1.38)
No	0.51 (0.48, 0.53)	Ref	0.50 (0.48, 0.53)	Ref	0.39 (0.37, 0.41)	Ref	0.39 (0.37, 0.41)	Ref
**Alcohol misuse**								
Yes	0.47 (0.41, 0.54)	0.90 (0.65, 1.25)	0.48 (0.42, 0.55)	0.94 (0.68, 1.31)	0.35 (0.28, 0.41)	0.82 (0.58, 1.16)	0.37 (0.30, 0.44)	0.87 (0.61, 1.23)
No	0.50 (0.48, 0.52)	Ref	0.50 (0.48, 0.52)	Ref	0.39 (0.37, 0.41)	Ref	0.40 (0.38, 0.41)	Ref
Not applicable (<21 years)	0.47 (0.38, 0.56)	0.87 (0.57, 1.33)	0.43 (0.35, 0.53)	0.75 (0.48, 1.16)	0.39 (0.30, 0.48)	1.00 (0.65, 1.56)	0.39 (0.30, 0.48)	0.96 (0.62, 1.48)
**Past month marijuana or cannabis use**								
Yes	0.47 (0.44, 0.51)	0.88 (0.71, 1.08)	0.45 (0.42, 0.49)	**0.77 (0.63, 0.95)**	0.35 (0.32, 0.39)	0.81 (0.65, 1.01)	0.38 (0.34, 0.41)	0.90 (0.73, 1.11)
No	0.50 (0.48, 0.53)	Ref	0.51 (0.49, 0.53)	Ref	0.40 (0.37, 0.42)	Ref	0.40 (0.38, 0.42)	Ref
**Device access**								
Yes	0.49 (0.47, 0.51)	Ref	0.49 (0.47, 0.51)	Ref	0.38 (0.37, 0.40)	Ref	0.39 (0.38, 0.41)	Ref
No	0.45 (0.27, 0.64)	0.82 (0.33, 2.05)	0.45 (0.27, 0.64)	0.81 (0.32, 2.04)	0.29 (0.14, 0.49)	0.60 (0.22, 1.61)	0.27 (0.12, 0.50)	0.53 (0.17, 1.63)
**Internet access**								
Yes	0.49 (0.47, 0.51)	Ref	0.49 (0.47, 0.51)	Ref	0.38 (0.36, 0.40)	Ref	0.39 (0.37, 0.41)	Ref

© 2026 Tesfaye L et al. *JAMA Network Open*.

Characteristics	Make sure you understand		Explain things		Spend enough time		Help deal with uncertainty	
	PM (95%CI)	aOR (95% CI)	PM (95% CI)	aOR (95% CI)	PM (95% CI)	aOR (95% CI)	PM (95% CI)	aOR (95% CI)
No	0.47 (0.32, 0.62)	0.90 (0.43, 1.86)	0.51 (0.37, 0.65)	1.09 (0.54, 2.18)	0.44 (0.30, 0.59)	1.31 (0.64, 2.69)	0.47 (0.32, 0.62)	1.44 (0.71, 2.92)
**Digital health literacy**								
Using technology to process health information	0.51 (0.49, 0.53)	**0.67 (0.49, 0.90)**	0.51 (0.49, 0.54)	**0.62 (0.45, 0.85)**	0.40 (0.37, 0.42)	0.75 (0.54, 1.04)	0.41 (0.39, 0.44)	**0.66 (0.48, 0.90)**
	0.46 (0.43, 0.49)		0.46 (0.43, 0.49)		0.36 (0.34, 0.39)		0.36 (0.34, 0.39)	
Understanding of health concepts and language	0.47 (0.44, 0.49)	**1.52 (1.13, 2.05)**	0.45 (0.42, 0.48)	**2.02 (1.49, 2.74)**	0.37 (0.34, 0.39)	1.32 (0.97, 1.80)	0.37 (0.34, 0.40)	**1.48 (1.09, 2.01)**
	0.53 (0.50, 0.55)		0.55 (0.52, 0.58)		0.40 (0.37, 0.43)		0.42 (0.39, 0.45)	
Ability to actively engage with digital services	0.49 (0.47, 0.51)	1.04 (0.80, 1.34)	0.48 (0.46, 0.50)	1.22 (0.94, 1.59)	0.39 (0.37, 0.41)	0.86 (0.65, 1.13)	0.40 (0.38, 0.43)	0.81 (0.62, 1.06)
	0.50 (0.47, 0.52)		0.51 (0.48, 0.54)		0.37 (0.35, 0.40)		0.38 (0.35, 0.40)	
Feel safe and in control	0.47 (0.45, 0.50)	**1.34 (1.10, 1.64)**	0.48 (0.46, 0.50)	1.21 (0.99, 1.48)	0.35 (0.32, 0.37)	**1.60 (1.28, 1.99)**	0.36 (0.34, 0.39)	**1.45 (1.17, 1.80)**
	0.51 (0.49, 0.54)		0.50 (0.48, 0.53)		0.41 (0.39, 0.43)		0.41 (0.39, 0.44)	
Motivated to engage with digital services	0.49 (0.46, 0.51)	1.13 (0.83, 1.53)	0.49 (0.46, 0.51)	1.15 (0.83, 1.60)	0.38 (0.35, 0.40)	1.08 (0.77, 1.51)	0.38 (0.35, 0.40)	1.35 (0.97, 1.88)
	0.50 (0.47, 0.54)		0.50 (0.47, 0.54)		0.39 (0.36, 0.42)		0.42 (0.38, 0.45)	
Access to digital services that work	0.46 (0.43, 0.49)	**1.49 (1.09, 2.04)**	0.46 (0.43, 0.49)	**1.45 (1.06, 1.99)**	0.35 (0.31, 0.38)	**1.55 (1.11, 2.16)**	0.35 (0.32, 0.39)	**1.64 (1.17, 2.31)**
	0.52 (0.49, 0.55)		0.52 (0.49, 0.55)		0.41 (0.38, 0.44)		0.42 (0.40, 0.45)	
Digital services that suit individual needs	0.46 (0.43, 0.49)	**1.37 (1.06, 1.77)**	0.48 (0.45, 0.51)	1.16 (0.89, 1.50)	0.34 (0.31, 0.37)	**1.58 (1.21, 2.07)**	0.36 (0.33, 0.39)	**1.36 (1.04, 1.78)**
	0.52 (0.49, 0.54)		0.50 (0.48, 0.53)		0.41 (0.39, 0.44)		0.41(0.39, 0.44)	

© 2026 Tesfaye L et al. *JAMA Network Open*.

Characteristics	Make sure you understand		Explain things		Spend enough time		Help deal with uncertainty	
	PM (95%CI)	aOR (95% CI)	PM (95% CI)	aOR (95% CI)	PM (95% CI)	aOR (95% CI)	PM (95% CI)	aOR (95% CI)
**MHSVI**								
Most vulnerable counties	0.49 (0.46, 0.51)	0.93 (0.77, 1.11)	0.49 (0.46, 0.51)	0.96 (0.81, 1.15)	0.38 (0.35, 0.40)	0.94 (0.78, 1.13)	0.40 (0.37, 0.42)	1.04 (0.87, 1.25)
Least vulnerable counties	0.50 (0.47, 0.53)	Ref	0.50 (0.47, 0.52)	Ref	0.39 (0.36, 0.42)	Ref	0.39 (0.36, 0.42)	Ref

Abbreviations: MHSVI= Minority Health Social Vulnerability Index, PM= predicted marginals, aOR= adjusted odds ratio, CI= confidence interval, Ref= reference group, AIAN= American Indian or Alaska Native, NHPI= Native Hawaiian or Pacific Islander, AA= African American.
Logistic regression analysis modeled the probability of 1= always. Bolded cells represent significant results where the confidence interval does not include the null value, 1.
Predicted marginals are shown for top age of the 4 age brackets as they appear in Table 1, whereas predicted marginals for digital health literacy are shown for 25th and 75th percentile of the domain score.

© 2026 Tesfaye L et al. *JAMA Network Open*.

eTable 8. Factors associated with optimal patient-centered communication among those who had 1 or more telehealth visits in the past year and resided in MHSVI most vulnerable counties, *n* = 1505, imputed data

Characteristics	Give chance to ask questions		Give attention to feelings		Involve you in decisions	
	PM (95% CI)	aOR (95% CI)	PM (95% CI)	aOR (95% CI)	PM (95% CI)	aOR (95% CI)
**Age**						
18	0.43 (0.38, 0.49)	1.00 (0.99, 1.01)	0.42 (0.37, 0.47)	1.00 (0.99, 1.00)	0.43 (0.38, 0.48)	1.00 (0.99, 1.01)
30	0.44 (0.40, 0.47)		0.42 (0.39, 0.45)		0.43 (0.40, 0.47)	
45	0.45 (0.42, 0.47)		0.42 (0.39, 0.45)		0.44 (0.41, 0.46)	
60	0.45 (0.41, 0.50)		0.42 (0.38, 0.46)		0.44 (0.40, 0.49)	
**Sex**						
Female	0.45 (0.42, 0.48)	1.07 (0.84, 1.36)	0.44 (0.41, 0.47)	1.24 (0.97, 1.57)	0.44 (0.41, 0.48)	1.08 (0.85, 1.37)
Male	0.43 (0.40, 0.48)	Ref	0.39 (0.35, 0.43)	Ref	0.43 (0.39, 0.47)	Ref
**Sexual orientation**						
Heterosexual	0.44 (0.41, 0.47)	Ref	0.42 (0.39, 0.45)	Ref	0.44 (0.41, 0.46)	Ref
Gay, lesbian, bisexual	0.48 (0.41, 0.55)	1.20 (0.84, 1.71)	0.42 (0.35, 0.49)	0.97 (0.68, 1.38)	0.43 (0.36, 0.51)	0.98 (0.69, 1.41)
**Race and/or ethnicity**						
AIAN, Asian, NHPI	0.44 (0.33, 0.54)	0.91 (0.54, 1.53)	0.33 (0.24, 0.43)	**0.59 (0.35, 0.99)**	0.33 (0.24, 0.43)	**0.56 (0.33, 0.93)**
Black or AA	0.41 (0.36, 0.46)	0.79 (0.59, 1.06)	0.42 (0.37, 0.46)	0.88 (0.66, 1.18)	0.43 (0.38, 0.48)	0.89 (0.66, 1.19)
Hispanic or Latino	0.47 (0.42, 0.52)	1.05 (0.78, 1.41)	0.40 (0.35, 0.45)	0.83 (0.62, 1.11)	0.45 (0.40, 0.50)	0.98 (0.73, 1.31)
White	0.46 (0.42, 0.49)	Ref	0.44 (0.40, 0.48)	Ref	0.45 (0.41, 0.49)	Ref
**Education**						
<High school	0.50 (0.44, 0.56)	**0.86 (0.75, 0.99)**	0.46 (0.40, 0.52)	0.89 (0.77, 1.02)	0.51 (0.45, 0.57)	**0.83 (0.73, 0.96)**
High school graduate	0.47 (0.44, 0.51)		0.44 (0.40, 0.47)		0.47 (0.43, 0.50)	
Vocational school, some college	0.44 (0.41, 0.47)		0.42 (0.39, 0.44)		0.43 (0.41, 0.46)	
College graduate or higher	0.41 (0.37, 0.45)		0.39 (0.35, 0.44)		0.39 (0.35, 0.44)	
**Income**						
<$20,000	0.46 (0.42, 0.50)	0.94 (0.83, 1.05)	0.42 (0.39, 0.46)	0.98 (0.87, 1.10)	0.43 (0.39, 0.47)	1.03 (0.91, 1.16)
$20,000 to $49,999	0.45 (0.42, 0.47)		0.42 (0.40, 0.45)		0.44 (0.41, 0.46)	
$50,000 to $74,999	0.43 (0.40, 0.47)		0.42 (0.39, 0.45)		0.44 (0.41, 0.47)	
≥$75,000	0.42 (0.37, 0.47)		0.41 (0.36, 0.46)		0.45 (0.40, 0.50)	
**English proficiency**						
Very well	0.46 (0.43, 0.49)	Ref	0.43 (0.40, 0.45)	Ref	0.45 (0.42, 0.48)	Ref

© 2026 Tesfaye L et al. *JAMA Network Open*.

Characteristics	Give chance to ask questions		Give attention to feelings		Involve you in decisions	
	PM (95% CI)	aOR (95% CI)	PM (95% CI)	aOR (95% CI)	PM (95% CI)	aOR (95% CI)
Well, not well, not at all	0.32 (0.25, 0.40)	**0.52 (0.35, 0.76)**	0.37 (0.30, 0.44)	0.75 (0.51, 1.09)	0.33 (0.27, 0.41)	**0.58 (0.39, 0.85)**
**Health insurance**						
Insured	0.45 (0.42, 0.48)	Ref	0.42 (0.40, 0.45)	Ref	0.44 (0.41, 0.47)	Ref
Uninsured	0.41 (0.34, 0.49)	0.84 (0.57, 1.24)	0.38 (0.31, 0.46)	0.81 (0.54, 1.19)	0.40 (0.32, 0.48)	0.81 (0.55, 1.20)
**General health**						
Excellent, very good, good	0.45 (0.42, 0.48)	Ref	0.43 (0.40, 0.46)	Ref	0.44 (0.41, 0.47)	Ref
Fair, poor	0.43 (0.38, 0.48)	0.89 (0.66, 1.19)	0.40 (0.35, 0.46)	0.90 (0.67, 1.21)	0.42 (0.37, 0.47)	0.88 (0.66, 1.18)
**Mental health**						
Excellent, very good, good	0.46 (0.43, 0.49)	Ref	0.43 (0.40, 0.46)	Ref	0.44 (0.41, 0.47)	Ref
Fair, poor	0.41 (0.37, 0.46)	0.81 (0.61, 1.07)	0.39 (0.35, 0.44)	0.82 (0.63, 1.08)	0.42 (0.37, 0.47)	0.89 (0.68, 1.18)
**Has a primary care clinician**						
Yes	0.50 (0.46, 0.54)	Ref	0.46 (0.43, 0.50)	Ref	0.49 (0.45, 0.52)	Ref
No	0.38 (0.35, 0.42)	**0.59 (0.46, 0.75)**	0.37 (0.33, 0.41)	**0.65 (0.51, 0.82)**	0.38 (0.34, 0.42)	**0.60 (0.47, 0.76)**
**Presence of underlying clinical conditions**						
Yes	0.48 (0.44, 0.53)	1.30 (1.00, 1.70)	0.42 (0.37, 0.46)	0.99 (0.75, 1.30)	0.47 (0.42, 0.51)	1.25 (0.95, 1.63)
No	0.42 (0.39, 0.46)	Ref	0.42 (0.39, 0.45)	Ref	0.42 (0.39, 0.45)	Ref
**Past year in-person visit(s) with healthcare clinician**						
0 times	0.46 (0.38, 0.55)	Ref	0.42 (0.33, 0.51)	Ref	0.44 (0.36, 0.53)	Ref
1 or more times	0.44 (0.42, 0.47)	0.90 (0.59, 1.39)	0.42 (0.39, 0.45)	1.01 (0.65, 1.57)	0.44 (0.41, 0.46)	0.96 (0.63, 1.47)
**History of COVID-19 infection**						
Yes	0.47 (0.43, 0.52)	1.21 (0.95, 1.54)	0.44 (0.40, 0.49)	1.17 (0.92, 1.50)	0.46 (0.41, 0.50)	1.14 (0.89, 1.45)
No	0.43 (0.40, 0.46)	Ref	0.41 (0.38, 0.44)	Ref	0.43 (0.40, 0.46)	Ref
**Body mass index**						
Healthy (18.5 to <25)	0.41 (0.37, 0.47)	Ref	0.41 (0.36, 0.46)	Ref	0.43 (0.38, 0.48)	Ref

© 2026 Tesfaye L et al. *JAMA Network Open*.

Characteristics	Give chance to ask questions		Give attention to feelings		Involve you in decisions	
	PM (95% CI)	aOR (95% CI)	PM (95% CI)	aOR (95% CI)	PM (95% CI)	aOR (95% CI)
Unhealthy (<18.5 or ≥25)	0.46 (0.43, 0.49)	1.20 (0.92, 1.57)	0.42 (0.39, 0.45)	1.07 (0.81, 1.40)	0.44 (0.41, 0.47)	1.06 (0.80, 1.39)
**Physical activity**						
Sufficient (≥150 minutes per week)	0.45 (0.42, 0.48)	Ref	0.42 (0.39, 0.45)	Ref	0.43 (0.40, 0.46)	Ref
Insufficient (<150 minutes per week)	0.44 (0.40, 0.49)	0.98 (0.76, 1.27)	0.43 (0.38, 0.48)	1.05 (0.81, 1.36)	0.45 (0.40, 0.49)	1.05 (0.81, 1.36)
**Past month cigarette smoking**						
Yes	0.42 (0.38, 0.47)	0.83 (0.63, 1.09)	0.40 (0.36, 0.44)	0.82 (0.62, 1.08)	0.41 (0.36, 0.45)	0.77 (0.59, 1.02)
No	0.46 (0.43, 0.50)	Ref	0.44 (0.40, 0.47)	Ref	0.46 (0.42, 0.50)	Ref
**Past month e-cigarette use**						
Yes	0.43 (0.38, 0.49)	0.91 (0.67, 1.25)	0.38 (0.33, 0.44)	0.78 (0.57, 1.07)	0.39 (0.34, 0.45)	0.74 (0.54, 1.02)
No	0.45 (0.42, 0.48)	Ref	0.43 (0.40, 0.47)	Ref	0.45 (0.42, 0.49)	Ref
**Alcohol misuse**						
Yes	0.51 (0.41, 0.60)	1.31 (0.84, 2.05)	0.47 (0.38, 0.57)	1.29 (0.81, 2.06)	0.47 (0.38, 0.57)	1.17 (0.74, 1.85)
No	0.44 (0.42, 0.47)	Ref	0.42 (0.39, 0.44)	Ref	0.44 (0.41, 0.47)	Ref
Not applicable (<21 years)	0.37 (0.27, 0.49)	0.71 (0.40, 1.25)	0.42 (0.32, 0.54)	1.03 (0.60, 1.77)	0.35 (0.25, 0.47)	0.66 (0.37, 1.18)
**Past month marijuana or cannabis use**						
Yes	0.45 (0.40, 0.49)	1.00 (0.76, 1.32)	0.42 (0.37, 0.46)	0.97 (0.73, 1.28)	0.42 (0.37, 0.47)	0.88 (0.67, 1.17)
No	0.44 (0.41, 0.48)	Ref	0.42 (0.39, 0.46)	Ref	0.45 (0.41, 0.48)	Ref
**Device access**						
Yes	0.45 (0.42, 0.47)	Ref	0.42 (0.40, 0.45)	Ref	0.44 (0.41, 0.47)	Ref
No	0.28 (0.11, 0.55)	0.45 (0.13, 1.57)	0.18 (0.05, 0.45)	0.25 (0.05, 1.09)	0.27 (0.10, 0.52)	0.42 (0.12, 1.45)
**Internet access**						
Yes	0.44 (0.42, 0.47)	Ref	0.42 (0.40, 0.45)	Ref	0.44 (0.41, 0.46)	Ref
No	0.45 (0.29, 0.62)	1.01 (0.46, 2.23)	0.34 (0.19, 0.54)	0.68 (0.27, 1.71)	0.48 (0.30, 0.67)	1.24 (0.49, 3.10)
**Digital health literacy**						

© 2026 Tesfaye L et al. *JAMA Network Open*.

Characteristics	Give chance to ask questions		Give attention to feelings		Involve you in decisions	
	PM (95% CI)	aOR (95% CI)	PM (95% CI)	aOR (95% CI)	PM (95% CI)	aOR (95% CI)
Using technology to process health information	0.45 (0.42, 0.48)<br>0.44 (0.40, 0.48)	0.88 (0.59, 1.33)	0.44 (0.40, 0.47)<br>0.40 (0.36, 0.44)	0.72 (0.47, 1.11)	0.45 (0.42, 0.49)<br>0.42 (0.38, 0.45)	0.75 (0.50, 1.12)
Understanding of health concepts and language	0.43 (0.39, 0.46)<br>0.47 (0.43, 0.51)	1.37 (0.91, 2.05)	0.40 (0.37, 0.44)<br>0.44 (0.40, 0.47)	1.26 (0.84, 1.91)	0.43 (0.39, 0.47)<br>0.44 (0.41, 0.48)	1.11 (0.73, 1.68)
Ability to actively engage with digital services	0.46 (0.43, 0.49)<br>0.43 (0.39, 0.46)	0.80 (0.57, 1.14)	0.43 (0.40, 0.47)<br>0.40 (0.37, 0.44)	0.78 (0.55, 1.11)	0.42 (0.38, 0.45)<br>0.46 (0.43, 0.50)	1.42 (0.99, 2.02)
Feel safe and in control	0.45 (0.42, 0.49)<br>0.44 (0.41, 0.47)	0.91 (0.68, 1.22)	0.38 (0.35, 0.42)<br>0.44 (0.41, 0.47)	**1.58 (1.17, 2.13)**	0.41 (0.38, 0.44)<br>0.46 (0.43, 0.49)	**1.40 (1.06, 1.86)**
Motivated to engage with digital services	0.44 (0.41, 0.47)<br>0.45 (0.41, 0.50)	1.10 (0.71, 1.69)	0.40 (0.37, 0.44)<br>0.44 (0.40, 0.48)	1.31 (0.84, 2.03)	0.43 (0.40, 0.47)<br>0.44 (0.40, 0.48)	1.06 (0.67, 1.65)
Access to digital services that work	0.37 (0.33, 0.42)<br>0.50 (0.46, 0.54)	**2.29 (1.50, 3.49)**	0.39 (0.34, 0.44)<br>0.44 (0.40, 0.48)	1.42 (0.91, 2.21)	0.41 (0.37, 0.46)<br>0.46 (0.42, 0.50)	1.36 (0.87, 2.12)
Digital services that suit individual needs	0.42 (0.38, 0.46)<br>0.46 (0.43, 0.49)	1.26 (0.90, 1.76)	0.36 (0.32, 0.41)<br>0.45 (0.42, 0.48)	**1.70 (1.20, 2.41)**	0.41 (0.37, 0.45)<br>0.45 (0.42, 0.49)	1.29 (0.92, 1.81)

© 2026 Tesfaye L et al. *JAMA Network Open*.

eTable 8. Factors associated with optimal patient-centered communication among those who had 1 or more telehealth visits in the past year and resided in MHSVI most vulnerable counties, *n*= 1505, imputed data (continued)

Characteristics	Make sure you understand		Explain things		Spend enough time		Help deal with uncertainty	
	PM (95% CI)	aOR (95% CI)	PM (95% CI)	aOR (95% CI)	PM (95% CI)	aOR (95% CI)	PM (95% CI)	aOR (95% CI)
**Age**								
18	0.48 (0.43, 0.54)	0.99 (0.99, 1.00)	0.46 (0.40, 0.51)	1.00 (0.99, 1.01)	0.33 (0.29, 0.39)	1.00 (0.99, 1.01)	0.39 (0.33, 0.44)	1.00 (0.99, 1.01)
30	0.48 (0.45, 0.52)		0.47 (0.43, 0.50)		0.35 (0.32, 0.39)		0.39 (0.36, 0.43)	
45	0.48 (0.45, 0.51)		0.48 (0.46, 0.51)		0.37 (0.35, 0.40)		0.40 (0.38, 0.43)	
60	0.48 (0.44, 0.52)		0.49 (0.45, 0.54)		0.40 (0.35, 0.44)		0.41 (0.37, 0.45)	
**Sex**								
Female	0.47 (0.44, 0.51)	0.93 (0.73, 1.18)	0.49 (0.46, 0.53)	1.17 (0.92, 1.49)	0.39 (0.36, 0.43)	**1.31 (1.02, 1.67)**	0.41 (0.38, 0.44)	1.14 (0.89, 1.44)
Male	0.49 (0.45, 0.53)	Ref	0.46 (0.42, 0.50)	Ref	0.34 (0.30, 0.38)	Ref	0.38 (0.34, 0.42)	Ref
**Sexual orientation**								
Heterosexual	0.48 (0.45, 0.50)	Ref	0.47 (0.44, 0.50)	Ref	0.37 (0.34, 0.39)	Ref	0.40 (0.37, 0.43)	Ref
Gay, lesbian, bisexual	0.52 (0.45, 0.59)	1.21 (0.85, 1.72)	0.55 (0.47, 0.62)	1.43 (0.99, 2.08)	0.40 (0.33, 0.47)	1.15 (0.80, 1.65)	0.39 (0.32, 0.46)	0.94 (0.66, 1.34)
**Race and/or ethnicity**								
AIAN, Asian, NHPI	0.49 (0.38, 0.59)	1.00 (0.59, 1.68)	0.44 (0.34, 0.55)	0.80 (0.47, 1.34)	0.38 (0.29, 0.48)	0.93 (0.56, 1.53)	0.32 (0.23, 0.42)	0.63 (0.38, 1.05)
Black or AA	0.47 (0.42, 0.52)	0.92 (0.69, 1.22)	0.48 (0.43, 0.53)	0.95 (0.71, 1.26)	0.33 (0.28, 0.38)	**0.73 (0.54, 0.98)**	0.38 (0.33, 0.42)	0.85 (0.63, 1.13)
Hispanic or Latino	0.48 (0.43, 0.53)	0.99 (0.73, 1.33)	0.46 (0.41, 0.51)	0.84 (0.62, 1.14)	0.37 (0.32, 0.42)	0.88 (0.65, 1.19)	0.42 (0.37, 0.47)	1.05 (0.78, 1.41)
White	0.49 (0.45, 0.52)	Ref	0.49 (0.46, 0.53)	Ref	0.39 (0.36, 0.43)	Ref	0.41 (0.37, 0.45)	Ref
**Education**								
<High school	0.54 (0.48, 0.60)	**0.85 (0.74, 0.98)**	0.52 (0.46, 0.58)	0.90 (0.78, 1.04)	0.43 (0.37, 0.49)	**0.85 (0.74, 0.99)**	0.47 (0.41, 0.53)	**0.84 (0.73, 0.96)**

© 2026 Tesfaye L et al. *JAMA Network Open*.

Characteristics	Make sure you understand		Explain things		Spend enough time		Help deal with uncertainty	
	PM (95% CI)	aOR (95% CI)	PM (95% CI)	aOR (95% CI)	PM (95% CI)	aOR (95% CI)	PM (95% CI)	aOR (95% CI)
High school graduate	0.51 (0.47, 0.54)		0.50 (0.46, 0.53)		0.40 (0.36, 0.43)		0.43 (0.40, 0.47)	
Vocational school, some college	0.48 (0.45, 0.50)		0.48 (0.45, 0.50)		0.37 (0.34, 0.39)		0.39 (0.37, 0.42)	
College graduate or higher	0.44 (0.40, 0.49)		0.45 (0.41, 0.50)		0.33 (0.29, 0.38)		0.36 (0.32, 0.40)	
**Income**								
<$20,000	0.49 (0.46, 0.53)	0.94 (0.84, 1.06)	0.48 (0.44, 0.52)	0.99 (0.88, 1.12)	0.37 (0.34, 0.41)	0.98 (0.87, 1.11)	0.42 (0.38, 0.46)	0.93 (0.82, 1.04)
$20,000 to $49,999	0.48 (0.46, 0.51)		0.48 (0.45, 0.50)		0.37 (0.35, 0.40)		0.40 (0.38, 0.43)	
$50,000 to $74,999	0.47 (0.44, 0.50)		0.48 (0.45, 0.51)		0.37 (0.34, 0.40)		0.39 (0.36, 0.42)	
≥$75,000	0.46 (0.41, 0.51)		0.48 (0.43, 0.53)		0.37 (0.32, 0.42)		0.37 (0.32, 0.42)	
**English proficiency**								
Very well	0.50 (0.48, 0.53)	Ref	0.49 (0.46, 0.52)	Ref	0.38 (0.36, 0.41)	Ref	0.42 (0.39, 0.44)	Ref
Well, not well, not at all	0.31 (0.25, 0.39)	**0.40 (0.27, 0.59)**	0.38 (0.31, 0.47)	**0.61 (0.40, 0.91)**	0.29 (0.22, 0.36)	**0.61 (0.41, 0.92)**	0.27 (0.21, 0.35)	**0.49 (0.33, 0.73)**
**Health insurance**								
Insured	0.49 (0.46, 0.51)	Ref	0.48 (0.46, 0.51)	Ref	0.37 (0.34, 0.39)	Ref	0.41 (0.38, 0.43)	Ref
Uninsured	0.42 (0.35, 0.50)	0.74 (0.50, 1.10)	0.45 (0.38, 0.53)	0.87 (0.59, 1.29)	0.39 (0.32, 0.47)	1.11 (0.76, 1.64)	0.33 (0.26, 0.41)	0.68 (0.46, 1.01)
**General health**								
Excellent, very good, good	0.48 (0.45, 0.52)	Ref	0.48 (0.45, 0.51)	Ref	0.38 (0.35, 0.41)	Ref	0.41 (0.38, 0.44)	Ref
Fair, poor	0.47 (0.42, 0.52)	0.93 (0.70, 1.25)	0.46 (0.41, 0.52)	0.91 (0.68, 1.22)	0.35 (0.30, 0.40)	0.84 (0.63, 1.14)	0.36 (0.32, 0.42)	0.79 (0.59, 1.06)
**Mental health**								
Excellent, very good, good	0.50 (0.47, 0.53)	Ref	0.50 (0.47, 0.53)	Ref	0.38 (0.35, 0.41)	Ref	0.41 (0.38, 0.44)	Ref
Fair, poor	0.44 (0.39, 0.49)	0.77 (0.58, 1.02)	0.43 (0.38, 0.48)	**0.72 (0.55, 0.95)**	0.34 (0.29, 0.39)	0.80 (0.60, 1.06)	0.38 (0.33, 0.42)	0.84 (0.64, 1.11)

© 2026 Tesfaye L et al. *JAMA Network Open*.

Characteristics	Make sure you understand		Explain things		Spend enough time		Help deal with uncertainty	
	PM (95% CI)	aOR (95% CI)	PM (95% CI)	aOR (95% CI)	PM (95% CI)	aOR (95% CI)	PM (95% CI)	aOR (95% CI)
**Has a primary care clinician**								
Yes	0.53 (0.50, 0.57)	Ref	0.52 (0.49, 0.56)	Ref	0.41 (0.38, 0.45)	Ref	0.43 (0.40, 0.47)	Ref
No	0.42 (0.39, 0.46)	**0.60 (0.47, 0.76)**	0.43 (0.39, 0.47)	**0.65 (0.51, 0.83)**	0.32 (0.29, 0.36)	**0.63 (0.49, 0.81)**	0.36 (0.33, 0.40)	**0.71 (0.56, 0.91)**
**Presence of underlying clinical conditions**								
Yes	0.49 (0.44, 0.53)	1.06 (0.80, 1.39)	0.52 (0.47, 0.57)	**1.34 (1.02, 1.76)**	0.39 (0.35, 0.44)	1.16 (0.87, 1.54)	0.40 (0.35, 0.44)	0.98 (0.74, 1.28)
No	0.48 (0.44. 0.51)	Ref	0.46 (0.42. 0.49)	Ref	0.36 (0.33. 0.39)	Ref	0.40 (0.37, 0.43)	Ref
**Past year in-person visit(s) with healthcare clinician**								
0 times	0.50 (0.41, 0.58)	Ref	0.49 (0.41, 0.58)	Ref	0.34 (0.26, 0.43)	Ref	0.38 (0.29, 0.47)	Ref
1 or more times	0.48 (0.45, 0.51)	0.92 (0.60, 1.40)	0.48 (0.45, 0.50)	0.93 (0.61, 1.41)	0.37 (0.35, 0.40)	1.18 (0.74, 1.85)	0.40 (0.38, 0.43)	1.13 (0.72, 1.75)
**History of COVID-19 infection**								
Yes	0.50 (0.45, 0.54)	1.13 (0.88, 1.45)	0.50 (0.45, 0.54)	1.13 (0.88, 1.44)	0.38 (0.34, 0.43)	1.10 (0.85, 1.40)	0.41 (0.37, 0.46)	1.09 (0.86, 1.39)
No	0.47 (0.44, 0.50)	Ref	0.47 (0.44, 0.50)	Ref	0.36 (0.34, 0.39)	Ref	0.39 (0.36, 0.42)	Ref
**Body mass index**								
Healthy (18.5 to <25)	0.45 (0.40, 0.51)	Ref	0.45 (0.40, 0.50)	Ref	0.37 (0.32, 0.42)	Ref	0.37 (0.32, 0.42)	Ref
Unhealthy (<18.5 or ≥25)	0.49 (0.46, 0.52)	1.18 (0.89, 1.56)	0.49 (0.46, 0.52)	1.17 (0.89, 1.54)	0.37 (0.34, 0.40)	1.01 (0.76, 1.34)	0.41 (0.38, 0.44)	1.18 (0.90, 1.56)
**Physical activity**								
Sufficient (≥150 minutes per week)	0.49 (0.46, 0.52)	Ref	0.48 (0.45, 0.51)	Ref	0.37 (0.34, 0.40)	Ref	0.39 (0.37, 0.42)	Ref

© 2026 Tesfaye L et al. *JAMA Network Open*.

Characteristics	Make sure you understand		Explain things		Spend enough time		Help deal with uncertainty	
	PM (95% CI)	aOR (95% CI)	PM (95% CI)	aOR (95% CI)	PM (95% CI)	aOR (95% CI)	PM (95% CI)	aOR (95% CI)
Insufficient (<150 minutes per week)	0.47 (0.42, 0.51)	0.91 (0.70, 1.17)	0.48 (0.44, 0.53)	1.04 (0.80, 1.34)	0.38 (0.33, 0.43)	1.06 (0.81, 1.39)	0.41 (0.37, 0.46)	1.09 (0.84, 1.41)
**Past month cigarette smoking**								
Yes	0.44 (0.40, 0.48)	**0.70 (0.53, 0.92)**	0.47 (0.42, 0.51)	0.90 (0.68, 1.19)	0.39 (0.34, 0.43)	1.13 (0.85, 1.50)	0.37 (0.33, 0.41)	0.77 (0.58, 1.02)
No	0.51 (0.48, 0.55)	Ref	0.49 (0.45, 0.52)	Ref	0.36 (0.33, 0.39)	Ref	0.42 (0.39, 0.46)	Ref
**Past month e-cigarette use**								
Yes	0.45 (0.40, 0.51)	0.83 (0.61, 1.14)	0.42 (0.37, 0.48)	**0.69 (0.50, 0.95)**	0.34 (0.29, 0.39)	0.79 (0.57, 1.10)	0.41 (0.35, 0.46)	1.05 (0.76, 1.44)
No	0.49 (0.46, 0.52)	Ref	0.50 (0.47, 0.53)	Ref	0.38 (0.35, 0.41)	Ref	0.40 (0.37, 0.43)	Ref
**Alcohol misuse**								
Yes	0.47 (0.38, 0.56)	0.91 (0.58, 1.44)	0.49 (0.40, 0.59)	1.04 (0.65, 1.67)	0.30 (0.22, 0.39)	0.66 (0.40, 1.09)	0.37 (0.28, 0.47)	0.84 (0.52, 1.36)
No	0.49 (0.46, 0.51)	Ref	0.48 (0.46, 0.51)	Ref	0.38 (0.35, 0.40)	Ref	0.41 (0.38, 0.43)	Ref
Not applicable (<21 years of age)	0.39 (0.28, 0.50)	0.62 (0.35, 1.08)	0.36 (0.26, 0.48)	**0.55 (0.31, 0.98)**	0.34 (0.25, 0.46)	0.84 (0.49, 1.46)	0.34 (0.24, 0.45)	0.72 (0.42, 1.25)
**Past month marijuana or cannabis use**								
Yes	0.46 (0.41, 0.50)	0.84 (0.64, 1.11)	0.46 (0.41, 0.50)	0.84 (0.63, 1.11)	0.34 (0.30, 0.39)	0.80 (0.60, 1.07)	0.40 (0.36, 0.45)	1.03 (0.77, 1.36)
No	0.49 (0.46, 0.53)	Ref	0.49 (0.46, 0.52)	Ref	0.39 (0.35, 0.42)	Ref	0.40 (0.37, 0.43)	Ref
**Device access**								
Yes	0.48 (0.46, 0.51)	Ref	0.48 (0.45, 0.50)	Ref	0.37 (0.35, 0.40)	Ref	0.40 (0.38, 0.43)	Ref
No	0.45 (0.25, 0.67)	0.87 (0.30, 2.51)	0.46 (0.25, 0.67)	0.89 (0.31, 2.58)	0.29 (0.13, 0.52)	0.64 (0.20, 2.01)	0.33 (0.14, 0.58)	0.70 (0.21, 2.30)
**Internet access**								
Yes	0.48 (0.46, 0.51)	Ref	0.48 (0.45, 0.50)	Ref	0.37 (0.35, 0.40)	Ref	0.40 (0.37, 0.42)	Ref

© 2026 Tesfaye L et al. *JAMA Network Open*.

Characteristics	Make sure you understand		Explain things		Spend enough time		Help deal with uncertainty	
	PM (95% CI)	aOR (95% CI)	PM (95% CI)	aOR (95% CI)	PM (95% CI)	aOR (95% CI)	PM (95% CI)	aOR (95% CI)
No	0.42 (0.26, 0.59)	0.74 (0.33, 1.67)	0.48 (0.30, 0.65)	0.98 (0.41, 2.31)	0.32 (0.17, 0.51)	0.76 (0.29, 1.95)	0.47 (0.30, 0.64)	1.36 (0.60, 3.10)
**Digital health literacy**								
Using technology to process health information	0.49 (0.46, 0.52)	0.78 (0.52, 1.17)	0.49 (0.45, 0.52)	0.85 (0.56, 1.28)	0.39 (0.35, 0.42)	0.74 (0.48, 1.14)	0.41 (0.38, 0.45)	0.76 (0.50, 1.17)
	0.46 (0.42, 0.50)		0.47 (0.43, 0.51)		0.35 (0.32, 0.39)		0.38 (0.35, 0.42)	
Understanding of health concepts and language	0.46 (0.42, 0.50)	1.43 (0.95, 2.16)	0.44 (0.40, 0.47)	**2.01 (1.32, 3.05)**	0.35 (0.31, 0.38)	1.46 (0.96, 2.22)	0.38 (0.34, 0.42)	1.35 (0.89, 2.06)
	0.51 (0.47, 0.55)		0.53 (0.49, 0.57)		0.39 (0.36, 0.43)		0.42 (0.38, 0.46)	
Ability to actively engage with digital services	0.49 (0.46, 0.52)	0.82 (0.58, 1.16)	0.48 (0.45, 0.51)	1.03 (0.72, 1.46)	0.39 (0.36, 0.42)	0.71 (0.49, 1.01)	0.41 (0.38, 0.45)	0.77 (0.53, 1.10)
	0.47 (0.43, 0.50)		0.48 (0.44, 0.52)		0.35 (0.32, 0.38)		0.38 (0.35, 0.42)	
Feel safe and in control	0.46 (0.43, 0.49)	1.32 (0.99, 1.75)	0.46 (0.42, 0.49)	1.33 (1.00, 1.76)	0.34 (0.30, 0.37)	**1.51 (1.12, 2.04)**	0.37 (0.33, 0.40)	**1.51 (1.12, 2.03)**
	0.50 (0.47, 0.53)		0.49 (0.46, 0.52)		0.39 (0.36, 0.42)		0.42 (0.39, 0.45)	
Motivated to engage with digital services	0.48 (0.44, 0.51)	1.07 (0.69, 1.66)	0.48 (0.44, 0.51)	1.03 (0.65, 1.63)	0.37 (0.34, 0.41)	1.00 (0.64, 1.57)	0.39 (0.35, 0.42)	1.22 (0.77, 1.92)
	0.49 (0.44, 0.53)		0.48 (0.44, 0.53)		0.37 (0.33, 0.41)		0.41 (0.37, 0.46)	
Access to digital services that work	0.44 (0.39, 0.48)	**1.72 (1.12, 2.65)**	0.46 (0.42, 0.50)	1.27 (0.82, 1.95)	0.33 (0.29, 0.38)	**1.62 (1.03, 2.53)**	0.37 (0.33, 0.42)	1.36 (0.85, 2.17)
	0.52 (0.48, 0.56)		0.49 (0.46, 0.53)		0.40 (0.36, 0.43)		0.42 (0.38, 0.46)	
Digital services that suit individual needs	0.45 (0.41, 0.49)	1.38 (0.99, 1.92)	0.46 (0.42, 0.51)	1.14 (0.81, 1.61)	0.31 (0.27, 0.35)	**1.86 (1.32, 2.62)**	0.35 (0.31, 0.40)	**1.59 (1.12, 2.25)**
	0.50 (0.47, 0.54)		0.49 (0.45, 0.52)		0.40 (0.37, 0.44)		0.43 (0.39, 0.46)	

Abbreviations: PM= predicted marginals, aOR= adjusted odds ratio, CI= confidence interval, Ref= reference group, AIAN= American Indian or Alaska Native, NHPI= Native Hawaiian or Pacific Islander, AA= African American.
Logistic regression analysis modeled the probability of 1= always. Bolded cells represent significant results where the confidence interval does not include the null value, 1.
Predicted marginals are shown for top age of the 4 age brackets as they appear in Table 1, whereas predicted marginals for digital health literacy are shown for 25*th* and 75*th* percentile of the domain score.

© 2026 Tesfaye L et al. *JAMA Network Open.*

eTable 9. Factors associated with optimal patient-centered communication among those who had 1 or more telehealth visits in the past year and resided in MHSVI least vulnerable counties, *n* = 1249, imputed data

Characteristics	Give chance to ask questions		Give attention to feelings		Involve you in decisions	
	PM (95% CI)	aOR (95% CI)	PM (95% CI)	aOR (95% CI)	PM (95% CI)	aOR (95% CI)
**Age**						
18	0.40 (0.34, 0.47)	1.00 (0.99, 1.01)	0.36 (0.30, 0.42)	1.00 (0.99, 1.01)	0.38 (0.32, 0.44)	1.01 (1.00, 1.02)
30	0.43 (0.38, 0.47)		0.38 (0.33, 0.42)		0.41 (0.37, 0.45)	
45	0.45 (0.43, 0.48)		0.40 (0.37, 0.43)		0.45 (0.42, 0.47)	
60	0.48 (0.45, 0.52)		0.43 (0.39, 0.46)		0.48 (0.45, 0.52)	
**Sex**						
Female	0.49 (0.45, 0.52)	**1.33 (1.01, 1.74)**	0.44 (0.41, 0.48)	**1.50 (1.15, 1.96)**	0.49 (0.45, 0.53)	**1.51 (1.16, 1.97)**
Male	0.43 (0.39, 0.47)	Ref	0.36 (0.32, 0.40)	Ref	0.41 (0.37, 0.45)	Ref
**Sexual orientation**						
Heterosexual	0.46 (0.43, 0.49)	Ref	0.40 (0.37, 0.43)	Ref	0.45 (0.42, 0.48)	Ref
Gay, lesbian, bisexual	0.48 (0.41, 0.56)	1.14 (0.77, 1.68)	0.43 (0.36, 0.51)	1.15 (0.78, 1.69)	0.47 (0.40, 0.55)	1.12 (0.76, 1.66)
**Race and/or ethnicity**						
AIAN, Asian, NHPI	0.33 (0.23, 0.44)	**0.51 (0.28, 0.92)**	0.26 (0.17, 0.37)	**0.42 (0.22, 0.80)**	0.31 (0.21, 0.44)	**0.47 (0.24, 0.92)**
Black or AA	0.49 (0.39, 0.58)	1.13 (0.69, 1.85)	0.40 (0.31, 0.51)	0.94 (0.56, 1.57)	0.49 (0.40, 0.58)	1.18 (0.73, 1.89)
Hispanic or Latino	0.49 (0.40, 0.58)	1.13 (0.71, 1.79)	0.40 (0.31, 0.49)	0.91 (0.57, 1.43)	0.46 (0.38, 0.55)	1.02 (0.65, 1.60)
White	0.46 (0.43, 0.49)	Ref	0.42 (0.39, 0.45)	Ref	0.46 (0.43, 0.49)	Ref
**Education**						
<High school	0.46 (0.38, 0.54)	1.00 (0.84, 1.19)	0.41 (0.33, 0.49)	0.99 (0.83, 1.19)	0.51 (0.43, 0.59)	0.87 (0.73, 1.04)
High school graduate	0.46 (0.41, 0.51)		0.40 (0.36, 0.45)		0.48 (0.43, 0.53)	
Vocational school, some college	0.46 (0.43, 0.49)		0.40 (0.38, 0.43)		0.45 (0.43, 0.48)	
College graduate or higher	0.46 (0.42, 0.50)		0.40 (0.36, 0.45)		0.43 (0.39, 0.47)	
**Income**						
<$20,000	0.46 (0.41, 0.52)	0.99 (0.87, 1.13)	0.41 (0.36, 0.47)	0.97 (0.85, 1.12)	0.46 (0.41, 0.52)	0.97 (0.84, 1.11)
$20,000 to $49,999	0.46 (0.43, 0.49)		0.41 (0.37, 0.44)		0.46 (0.42, 0.49)	
$50,000 to $74,999	0.46 (0.43, 0.49)		0.40 (0.38, 0.43)		0.45 (0.42, 0.48)	
≥$75,000	0.46 (0.41, 0.50)		0.40 (0.36, 0.44)		0.44 (0.40, 0.49)	
**English proficiency**						

© 2026 Tesfaye L et al. *JAMA Network Open*.

Characteristics	Give chance to ask questions		Give attention to feelings		Involve you in decisions	
	PM (95% CI)	aOR (95% CI)	PM (95% CI)	aOR (95% CI)	PM (95% CI)	aOR (95% CI)
Very well	0.48 (0.45, 0.51)	Ref	0.41 (0.39, 0.44)	Ref	0.47 (0.44, 0.50)	Ref
Well, not well, not at all	0.23 (0.15, 0.33)	**0.28 (0.15, 0.50)**	0.28 (0.19, 0.38)	**0.48 (0.28, 0.84)**	0.24 (0.16, 0.34)	**0.30 (0.17, 0.53)**
**Health insurance**						
Insured	0.46 (0.43, 0.49)	Ref	0.41 (0.38, 0.44)	Ref	0.45 (0.42, 0.48)	Ref
Uninsured	0.41 (0.29, 0.55)	0.79 (0.41, 1.54)	0.34 (0.22, 0.49)	0.73 (0.36, 1.47)	0.44 (0.30, 0.58)	0.93 (0.45, 1.92)
**General health**						
Excellent, very good, good	0.45 (0.42, 0.48)	Ref	0.41 (0.37, 0.44)	Ref	0.44 (0.41, 0.47)	Ref
Fair, poor	0.49 (0.43, 0.55)	1.22 (0.88, 1.69)	0.40 (0.35, 0.46)	0.99 (0.71, 1.38)	0.49 (0.44, 0.55)	1.30 (0.94, 1.81)
**Mental health**						
Excellent, very good, good	0.48 (0.44, 0.51)	Ref	0.42 (0.39, 0.46)	Ref	0.47 (0.44, 0.51)	Ref
Fair, poor	0.42 (0.37, 0.47)	0.76 (0.55, 1.04)	0.36 (0.31, 0.41)	**0.72 (0.53, 0.99)**	0.40 (0.35, 0.46)	**0.71 (0.52, 0.96)**
**Has a primary care clinician**						
Yes	0.50 (0.46, 0.54)	Ref	0.45 (0.41, 0.48)	Ref	0.50 (0.46, 0.54)	Ref
No	0.40 (0.36, 0.44)	**0.61 (0.47, 0.81)**	0.34 (0.30, 0.39)	**0.61 (0.46, 0.80)**	0.38 (0.34, 0.43)	**0.57 (0.43, 0.75)**
**Presence of underlying clinical conditions**						
Yes	0.48 (0.44, 0.53)	1.22 (0.92, 1.62)	0.42 (0.38, 0.46)	1.12 (0.84, 1.49)	0.45 (0.41, 0.50)	1.01 (0.76, 1.34)
No	0.44 (0.40, 0.48)	Ref	0.39 (0.36, 0.43)	Ref	0.45 (0.41, 0.49)	Ref
**Past year in-person visit(s) with healthcare clinician**						
0 times	0.37 (0.29, 0.47)	Ref	0.35 (0.27, 0.44)	Ref	0.39 (0.30, 0.48)	Ref
1 or more times	0.47 (0.44, 0.50)	1.59 (0.99, 2.54)	0.41 (0.38, 0.44)	1.37 (0.86, 2.18)	0.46 (0.43, 0.49)	1.42 (0.89, 2.26)
**History of COVID-19 infection**						
Yes	0.48 (0.43, 0.53)	1.13 (0.85, 1.50)	0.44 (0.39, 0.49)	1.27 (0.95, 1.69)	0.52 (0.47, 0.57)	**1.61 (1.22, 2.14)**
No	0.45 (0.42, 0.48)	Ref	0.39 (0.36, 0.42)	Ref	0.42 (0.39, 0.45)	Ref
**Body mass index**						
Healthy (18.5 to <25)	0.45 (0.40, 0.50)	Ref	0.40 (0.35, 0.45)	Ref	0.45 (0.40, 0.50)	Ref

© 2026 Tesfaye L et al. *JAMA Network Open*.

Characteristics	Give chance to ask questions		Give attention to feelings		Involve you in decisions	
	PM (95% CI)	aOR (95% CI)	PM (95% CI)	aOR (95% CI)	PM (95% CI)	aOR (95% CI)
Unhealthy (<18.5 or ≥25)	0.46 (0.43, 0.50)	1.08 (0.80, 1.46)	0.41 (0.37, 0.44)	1.02 (0.75, 1.38)	0.45 (0.42, 0.49)	1.01 (0.75, 1.37)
**Physical activity**						
Sufficient (≥150 minutes per week)	0.47 (0.44, 0.51)	Ref	0.40 (0.37, 0.43)	Ref	0.46 (0.43, 0.49)	Ref
Insufficient (<150 minutes per week)	0.43 (0.39, 0.48)	0.81 (0.61, 1.06)	0.41 (0.37, 0.46)	1.07 (0.81, 1.41)	0.44 (0.39, 0.48)	0.89 (0.67, 1.18)
**Past month cigarette smoking**						
Yes	0.45 (0.39, 0.51)	0.94 (0.67, 1.33)	0.44 (0.38, 0.50)	1.27 (0.90, 1.80)	0.48 (0.42, 0.54)	1.21 (0.85, 1.72)
No	0.46 (0.43, 0.50)	Ref	0.39 (0.36, 0.42)	Ref	0.44 (0.41, 0.47)	Ref
**Past month e-cigarette use**						
Yes	0.41 (0.34, 0.48)	0.72 (0.49, 1.07)	0.37 (0.30, 0.44)	0.78 (0.53, 1.15)	0.43 (0.36, 0.50)	0.88 (0.59, 1.31)
No	0.47 (0.44, 0.50)	Ref	0.41 (0.38, 0.45)	Ref	0.46 (0.43, 0.49)	Ref
**Alcohol misuse**						
Yes	0.43 (0.33, 0.53)	0.85 (0.51, 1.42)	0.35 (0.26, 0.45)	0.75 (0.45, 1.25)	0.50 (0.40, 0.60)	1.25 (0.75, 2.08)
No	0.46 (0.43, 0.49)	Ref	0.40 (0.38, 0.43)	Ref	0.45 (0.42, 0.48)	Ref
Not applicable (<21 years of age)	0.51 (0.38, 0.65)	1.29 (0.66, 2.54)	0.51 (0.37, 0.65)	1.67 (0.83, 3.37)	0.41 (0.28, 0.56)	0.83 (0.41, 1.68)
**Past month marijuana or cannabis use**						
Yes	0.43 (0.38, 0.49)	0.84 (0.61, 1.17)	0.37 (0.31, 0.43)	0.78 (0.55, 1.09)	0.38 (0.33, 0.44)	**0.64 (0.46, 0.89)**
No	0.47 (0.44, 0.50)	Ref	0.42 (0.39, 0.45)	Ref	0.47 (0.44, 0.51)	Ref
**Device access**						
Yes	0.46 (0.43, 0.49)	Ref	0.40 (0.38, 0.43)	Ref	0.45 (0.43, 0.48)	Ref
No	0.32 (0.07, 0.75)	0.50 (0.05, 4.44)	0.43 (0.14, 0.76)	1.11 (0.19, 6.41)	0.34 (0.07, 0.77)	0.55 (0.05, 5.50)
**Internet access**						
Yes	0.46 (0.43, 0.49)	Ref	0.41 (0.38, 0.43)	Ref	0.45 (0.43, 0.48)	Ref
No	0.45 (0.19, 0.73)	0.95 (0.21, 4.15)	0.38 (0.16, 0.66)	0.90 (0.22, 3.54)	0.39 (0.15, 0.70)	0.73 (0.15, 3.46)
**Digital health literacy**						

© 2026 Tesfaye L et al. *JAMA Network Open*.

Characteristics	Give chance to ask questions		Give attention to feelings		Involve you in decisions	
	PM (95% CI)	aOR (95% CI)	PM (95% CI)	aOR (95% CI)	PM (95% CI)	aOR (95% CI)
Using technology to process health information	0.46 (0.43, 0.50)	0.95 (0.58, 1.53)	0.42 (0.38, 0.45)	0.79 (0.48, 1.32)	0.47 (0.44, 0.50)	0.63 (0.39, 1.00)
	0.46 (0.41, 0.50)		0.39 (0.34, 0.43)		0.42 (0.37, 0.46)	
Understanding of health concepts and language	0.45 (0.41, 0.49)	1.21 (0.78, 1.88)	0.38 (0.34, 0.42)	1.52 (0.96, 2.41)	0.44 (0.40, 0.47)	1.33 (0.86, 2.05)
	0.47 (0.43, 0.52)		0.43 (0.39, 0.48)		0.47 (0.43, 0.51)	
Ability to actively engage with digital services	0.46 (0.42, 0.49)	1.09 (0.73, 1.62)	0.41 (0.37, 0.44)	0.98 (0.66, 1.47)	0.43 (0.40, 0.47)	1.47 (0.98, 2.19)
	0.47 (0.42, 0.51)		0.40 (0.36, 0.45)		0.48 (0.44, 0.53)	
Feel safe and in control	0.44 (0.40, 0.47)	**1.42 (1.05, 1.92)**	0.38 (0.35, 0.41)	**1.46 (1.07, 1.98)**	0.43 (0.40, 0.47)	1.34 (1.00, 1.80)
	0.48 (0.45, 0.52)		0.43 (0.39, 0.46)		0.47 (0.44, 0.51)	
Motivated to engage with digital services	0.46 (0.43, 0.49)	0.97 (0.58, 1.61)	0.39 (0.36, 0.43)	1.30 (0.79, 2.15)	0.44 (0.41, 0.48)	1.31 (0.80, 2.12)
	0.46 (0.41, 0.51)		0.43 (0.38, 0.48)		0.48 (0.43, 0.53)	
Access to digital services that work	0.40 (0.36, 0.45)	**2.09 (1.31, 3.36)**	0.35 (0.30, 0.40)	**2.03 (1.25, 3.29)**	0.41 (0.36, 0.45)	**1.80 (1.12, 2.89)**
	0.51 (0.47, 0.55)		0.45 (0.41, 0.49)		0.49 (0.45, 0.54)	
Digital services that suit individual needs	0.45 (0.41, 0.50)	1.07 (0.71, 1.62)	0.40 (0.36, 0.44)	1.06 (0.70, 1.58)	0.44 (0.40, 0.49)	1.13 (0.74, 1.71)
	0.47 (0.42, 0.51)		0.41 (0.37, 0.45)		0.46 (0.42, 0.50)	

© 2026 Tesfaye L et al. *JAMA Network Open*.

eTable 9. Factors associated with optimal patient-centered communication among those who had 1 or more telehealth visits in the past year and resided in MHSVI least vulnerable counties, *n* = 1249, imputed data (continued)

Characteristics	Make sure you understand		Explain things		Spend enough time		Help deal with uncertainty	
	PM (95% CI)	aOR (95% CI)	PM (95% CI)	aOR (95% CI)	PM (95% CI)	aOR (95% CI)	PM (95% CI)	aOR (95% CI)
**Age**								
18	0.39 (0.33, 0.45)	**1.01 (1.01, 1.02)**	0.40 (0.34, 0.47)	1.01 (1.00, 1.02)	0.31 (0.26, 0.37)	1.01 (1.00, 1.02)	0.35 (0.29, 0.41)	1.00 (0.99, 1.01)
30	0.44 (0.40, 0.48)		0.45 (0.40, 0.49)		0.34 (0.31, 0.39)		0.37 (0.32, 0.41)	
45	0.50 (0.47, 0.53)		0.50 (0.47, 0.53)		0.39 (0.36, 0.42)		0.38 (0.36, 0.41)	
60	0.56 (0.52, 0.60)		0.56 (0.52, 0.59)		0.44 (0.40, 0.48)		0.40 (0.36, 0.44)	
**Sex**								
Female	0.55 (0.52, 0.59)	**1.62 (1.24, 2.12)**	0.54 (0.50, 0.58)	**1.42 (1.09, 1.84)**	0.42 (0.38, 0.46)	1.25 (0.95, 1.64)	0.40 (0.36, 0.44)	1.19 (0.90, 1.56)
Male	0.45 (0.41, 0.49)	Ref	0.47 (0.43, 0.51)	Ref	0.37 (0.34, 0.41)	Ref	0.37 (0.33, 0.41)	Ref
**Sexual orientation**								
Heterosexual	0.51 (0.48, 0.54)	Ref	0.50 (0.47, 0.53)	Ref	0.39 (0.36, 0.42)	Ref	0.38 (0.35, 0.41)	Ref
Gay, lesbian, bisexual	0.49 (0.41, 0.56)	0.88 (0.60, 1.28)	0.58 (0.50, 0.65)	1.46 (0.98, 2.18)	0.44 (0.37, 0.51)	1.26 (0.86, 1.85)	0.42 (0.35, 0.50)	1.23 (0.83, 1.82)
**Race and/or ethnicity**								
AIAN, Asian, NHPI	0.41 (0.30, 0.53)	0.61 (0.34, 1.12)	0.35 (0.25, 0.47)	**0.47 (0.26, 0.84)**	0.21 (0.12, 0.32)	**0.31 (0.15, 0.64)**	0.20 (0.12, 0.30)	**0.32 (0.17, 0.63)**
Black or AA	0.59 (0.49, 0.68)	1.50 (0.93, 2.43)	0.62 (0.52, 0.72)	**1.74 (1.03, 2.94)**	0.38 (0.29, 0.47)	0.82 (0.50, 1.35)	0.46 (0.36, 0.56)	1.38 (0.84, 2.28)
Hispanic or Latino	0.52 (0.43, 0.61)	1.07 (0.68, 1.69)	0.49 (0.41, 0.58)	0.93 (0.60, 1.45)	0.39 (0.31, 0.48)	0.88 (0.56, 1.38)	0.37 (0.29, 0.46)	0.88 (0.55, 1.39)
White	0.51 (0.47, 0.54)	Ref	0.51 (0.48, 0.54)	Ref	0.41 (0.38, 0.45)	Ref	0.39 (0.36, 0.42)	Ref
**Education**								
<High school	0.57 (0.49, 0.64)	0.86 (0.72, 1.03)	0.54 (0.46, 0.62)	0.92 (0.77, 1.09)	0.45 (0.37, 0.54)	0.88 (0.73, 1.05)	0.44 (0.36, 0.52)	0.88 (0.74, 1.06)

© 2026 Tesfaye L et al. *JAMA Network Open.*

Characteristics	Make sure you understand		Explain things		Spend enough time		Help deal with uncertainty	
	PM (95% CI)	aOR (95% CI)	PM (95% CI)	aOR (95% CI)	PM (95% CI)	aOR (95% CI)	PM (95% CI)	aOR (95% CI)
High school graduate	0.54 (0.49, 0.59)		0.53 (0.48, 0.57)		0.43 (0.38, 0.48)		0.41 (0.36, 0.46)	
Vocational school, some college	0.51 (0.48, 0.54)		0.51 (0.48, 0.54)		0.40 (0.37, 0.43)		0.39 (0.36, 0.41)	
College graduate or higher	0.48 (0.44, 0.52)		0.49 (0.45, 0.54)		0.38 (0.34, 0.42)		0.36 (0.32, 0.41)	
**Income**								
<$20,000	0.50 (0.44, 0.55)	1.02 (0.89, 1.17)	0.50 (0.44, 0.55)	1.03 (0.90, 1.17)	0.40 (0.35, 0.46)	0.99 (0.86, 1.13)	0.40 (0.35, 0.46)	0.95 (0.83, 1.09)
$20,000 to $49,999	0.50 (0.47, 0.54)		0.50 (0.47, 0.54)		0.40 (0.37, 0.43)		0.39 (0.36, 0.43)	
$50,000 to $74,999	0.51 (0.48, 0.54)		0.51 (0.48, 0.54)		0.40 (0.37, 0.43)		0.38 (0.35, 0.41)	
≥$75,000	0.51 (0.47, 0.56)		0.52 (0.47, 0.56)		0.40 (0.35, 0.44)		0.37 (0.33, 0.42)	
**English proficiency**								
Very well	0.52 (0.50, 0.55)	Ref	0.53 (0.50, 0.55)	Ref	0.41 (0.38, 0.44)	Ref	0.40 (0.37, 0.43)	Ref
Well, not well, not at all	0.31 (0.22, 0.41)	**0.35 (0.20, 0.59)**	0.29 (0.20, 0.40)	**0.31 (0.18, 0.55)**	0.27 (0.18, 0.37)	**0.47 (0.27, 0.83)**	0.22 (0.15, 0.32)	**0.39 (0.21, 0.69)**
**Health insurance**								
Insured	0.51 (0.48, 0.54)	Ref	0.51 (0.48, 0.54)	Ref	0.40 (0.37, 0.43)	Ref	0.39 (0.36, 0.42)	Ref
Uninsured	0.43 (0.28, 0.59)	0.66 (0.30, 1.44)	0.47 (0.33, 0.61)	0.81 (0.40, 1.66)	0.32 (0.19, 0.48)	0.67 (0.30, 1.49)	0.29 (0.17, 0.45)	0.59 (0.26, 1.34)
**General health**								
Excellent, very good, good	0.49 (0.46, 0.52)	Ref	0.50 (0.47, 0.53)	Ref	0.39 (0.36, 0.42)	Ref	0.38 (0.35, 0.41)	Ref
Fair, poor	0.56 (0.50, 0.62)	**1.41 (1.02, 1.96)**	0.53 (0.47, 0.59)	1.15 (0.83, 1.60)	0.43 (0.37, 0.49)	1.24 (0.88, 1.75)	0.40 (0.34, 0.46)	1.09 (0.78, 1.52)
**Mental health**								
Excellent, very good, good	0.53 (0.49, 0.56)	Ref	0.52 (0.49, 0.56)	Ref	0.44 (0.40, 0.47)	Ref	0.42 (0.39, 0.45)	Ref
Fair, poor	0.46 (0.41, 0.51)	**0.71 (0.52, 0.97)**	0.48 (0.42, 0.53)	0.80 (0.59, 1.09)	0.30 (0.25, 0.35)	**0.50 (0.36, 0.70)**	0.30 (0.26, 0.35)	**0.55 (0.40, 0.76)**

© 2026 Tesfaye L et al. *JAMA Network Open*.

Characteristics	Make sure you understand		Explain things		Spend enough time		Help deal with uncertainty	
	PM (95% CI)	aOR (95% CI)	PM (95% CI)	aOR (95% CI)	PM (95% CI)	aOR (95% CI)	PM (95% CI)	aOR (95% CI)
**Has a primary care clinician**								
Yes	0.57 (0.53, 0.60)	Ref	0.55 (0.51, 0.58)	Ref	0.43 (0.40, 0.47)	Ref	0.42 (0.39, 0.46)	Ref
No	0.42 (0.38, 0.47)	**0.50 (0.38, 0.65)**	0.45 (0.41, 0.50)	**0.63 (0.48, 0.83)**	0.35 (0.30, 0.39)	**0.64 (0.49, 0.85)**	0.33 (0.29, 0.37)	**0.62 (0.47, 0.82)**
**Presence of underlying clinical conditions**								
Yes	0.50 (0.46, 0.54)	0.91 (0.69, 1.20)	0.50 (0.46, 0.55)	0.94 (0.71, 1.26)	0.40 (0.36, 0.44)	1.01 (0.76, 1.34)	0.40 (0.36, 0.44)	1.13 (0.84, 1.50)
No	0.52 (0.48, 0.55)	Ref	0.51 (0.48, 0.55)	Ref	0.40 (0.36, 0.44)	Ref	0.37 (0.34, 0.41)	Ref
**Past year in-person visit(s) with healthcare clinician**								
0 times	0.45 (0.37, 0.54)	Ref	0.48 (0.39, 0.57)	Ref	0.40 (0.31, 0.49)	Ref	0.37 (0.29, 0.46)	Ref
1 or more times	0.51 (0.48, 0.54)	1.33 (0.84, 2.08)	0.51 (0.48, 0.54)	1.16 (0.74, 1.82)	0.40 (0.37, 0.43)	1.01 (0.64, 1.60)	0.39 (0.36, 0.41)	1.07 (0.67, 1.72)
**History of COVID-19 infection**								
Yes	0.54 (0.49, 0.59)	1.21 (0.91, 1.61)	0.53 (0.48, 0.58)	1.14 (0.87, 1.50)	0.43 (0.39, 0.48)	1.28 (0.97, 1.70)	0.41 (0.36, 0.46)	1.17 (0.88, 1.55)
No	0.50 (0.46, 0.53)	Ref	0.50 (0.47, 0.53)	Ref	0.38 (0.35, 0.42)	Ref	0.38 (0.34, 0.41)	Ref
**Body mass index**								
Healthy (18.5 to <25)	0.51 (0.45, 0.56)	Ref	0.52 (0.47, 0.57)	Ref	0.43 (0.38, 0.48)	Ref	0.40 (0.35, 0.45)	Ref
Unhealthy (<18.5 or ≥25)	0.51 (0.48, 0.54)	1.01 (0.75, 1.36)	0.50 (0.47, 0.54)	0.92 (0.69, 1.23)	0.39 (0.35, 0.42)	0.81 (0.59, 1.10)	0.38 (0.35, 0.41)	0.90 (0.66, 1.22)
**Physical activity**								
Sufficient (≥150 minutes per week)	0.52 (0.48, 0.55)	Ref	0.50 (0.46, 0.53)	Ref	0.40 (0.37, 0.43)	Ref	0.39 (0.36, 0.43)	Ref

© 2026 Tesfaye L et al. *JAMA Network Open*.

Characteristics	Make sure you understand		Explain things		Spend enough time		Help deal with uncertainty	
	PM (95% CI)	aOR (95% CI)	PM (95% CI)	aOR (95% CI)	PM (95% CI)	aOR (95% CI)	PM (95% CI)	aOR (95% CI)
Insufficient (<150 minutes per week)	0.49 (0.45, 0.54)	0.89 (0.68, 1.18)	0.53 (0.48, 0.57)	1.13 (0.86, 1.50)	0.40 (0.35, 0.44)	0.98 (0.74, 1.29)	0.37 (0.33, 0.42)	0.90 (0.67, 1.20)
**Past month cigarette smoking**								
Yes	0.53 (0.47, 0.59)	1.15 (0.81, 1.62)	0.52 (0.46, 0.58)	1.09 (0.78, 1.53)	0.40 (0.34, 0.46)	1.00 (0.71, 1.41)	0.41 (0.36, 0.47)	1.22 (0.86, 1.71)
No	0.50 (0.47, 0.53)	Ref	0.50 (0.47, 0.54)	Ref	0.40 (0.37, 0.43)	Ref	0.37 (0.34, 0.41)	Ref
**Past month e-cigarette use**								
Yes	0.46 (0.39, 0.53)	0.73 (0.49, 1.07)	0.50 (0.42, 0.57)	0.92 (0.62, 1.36)	0.38 (0.31, 0.45)	0.88 (0.59, 1.31)	0.41 (0.34, 0.48)	1.13 (0.77, 1.66)
No	0.52 (0.49, 0.55)	Ref	0.51 (0.48, 0.54)	Ref	0.40 (0.37, 0.43)	Ref	0.38 (0.35, 0.41)	Ref
**Alcohol misuse**								
Yes	0.50 (0.40, 0.59)	0.95 (0.57, 1.56)	0.47 (0.38, 0.57)	0.84 (0.52, 1.35)	0.40 (0.31, 0.50)	1.00 (0.61, 1.65)	0.36 (0.27, 0.46)	0.87 (0.52, 1.46)
No	0.51 (0.48, 0.54)	Ref	0.51 (0.48, 0.54)	Ref	0.40 (0.37, 0.43)	Ref	0.38 (0.36, 0.41)	Ref
Not applicable (<21 years of age)	0.56 (0.43, 0.69)	1.29 (0.66, 2.54)	0.52 (0.37, 0.66)	1.02 (0.49, 2.13)	0.43 (0.30, 0.58)	1.19 (0.57, 2.46)	0.45 (0.30, 0.60)	1.35 (0.63, 2.86)
**Past month marijuana or cannabis use**								
Yes	0.49 (0.43, 0.55)	0.89 (0.64, 1.23)	0.45 (0.39, 0.51)	**0.69 (0.50, 0.96)**	0.37 (0.32, 0.43)	0.84 (0.60, 1.18)	0.34 (0.29, 0.40)	0.76 (0.55, 1.05)
No	0.51 (0.48, 0.55)	Ref	0.53 (0.49, 0.56)	Ref	0.41 (0.38, 0.44)	Ref	0.40 (0.37, 0.43)	Ref
**Device access**								
Yes	0.51 (0.48, 0.54)	Ref	0.51 (0.48, 0.54)	Ref	0.40 (0.37, 0.43)	Ref	0.39 (0.36. 0.41)	Ref
No	0.42 (0.13, 0.77)	0.64 (0.09, 4.25)	0.40 (0.11, 0.78)	0.60 (0.08, 4.24)	0.25 (0.04, 0.69)	0.45 (0.05, 4.07)	0.09 (0.003, 0.76)	0.12 (0.002, 5.17)
**Internet access**								
Yes	0.51 (0.48, 0.54)	Ref	0.51 (0.48, 0.54)	Ref	0.40 (0.37, 0.42)	Ref	0.38 (0.36 0.41)	Ref

© 2026 Tesfaye L et al. *JAMA Network Open.*

Characteristics	Make sure you understand		Explain things		Spend enough time		Help deal with uncertainty	
	PM (95% CI)	aOR (95% CI)	PM (95% CI)	aOR (95% CI)	PM (95% CI)	aOR (95% CI)	PM (95% CI)	aOR (95% CI)
No	0.55 (0.27, 0.81)	1.25 (0.28, 5.59)	0.55 (0.30, 0.77)	1.22 (0.34, 4.29)	0.65 (0.40, 0.84)	**3.50 (1.02, 12.04)**	0.52 (0.24, 0.79)	1.97 (0.43, 8.90)
**Digital health literacy**								
Using technology to process health information	0.53 (0.50, 0.56)	**0.54 (0.34, 0.86)**	0.54 (0.51, 0.57)	**0.41 (0.25, 0.68)**	0.41 (0.37, 0.44)	0.78 (0.47, 1.28)	0.41 (0.38, 0.45)	**0.53 (0.32, 0.87)**
	0.46 (0.41, 0.50)		0.44 (0.39, 0.48)		0.38 (0.34, 0.43)		0.34 (0.30, 0.38)	
Understanding of health concepts and language	0.48 (0.44, 0.52)	**1.73 (1.10, 2.70)**	0.47 (0.43, 0.50)	**2.16 (1.37, 3.40)**	0.39 (0.35, 0.43)	1.20 (0.75, 1.93)	0.36 (0.32, 0.39)	**1.67 (1.05, 2.65)**
	0.55 (0.51, 0.59)		0.57 (0.52, 0.61)		0.41 (0.37, 0.45)		0.42 (0.38, 0.46)	
Ability to actively engage with digital services	0.49 (0.46, 0.52)	1.45 (0.98, 2.15)	0.49 (0.46, 0.52)	**1.52 (1.02, 2.26)**	0.39 (0.36, 0.43)	1.12 (0.74, 1.70)	0.39 (0.36, 0.42)	0.90 (0.60, 1.37)
	0.54 (0.50, 0.58)		0.54 (0.50, 0.59)		0.41 (0.37, 0.45)		0.38 (0.34, 0.42)	
Feel safe and in control	0.49 (0.46, 0.52)	**1.42 (1.07, 1.90)**	0.50 (0.47, 0.53)	1.11 (0.82, 1.50)	0.36 (0.33, 0.40)	**1.76 (1.27, 2.44)**	0.36 (0.33, 0.40)	**1.43 (1.04, 1.97)**
	0.53 (0.50, 0.57)		0.52 (0.48, 0.55)		0.43 (0.40, 0.47)		0.41 (0.37, 0.44)	
Motivated to engage with digital services	0.50 (0.47, 0.53)	1.23 (0.78, 1.92)	0.50 (0.46, 0.53)	1.39 (0.85, 2.28)	0.39 (0.36, 0.43)	1.12 (0.67, 1.89)	0.36 (0.33, 0.40)	**1.69 (1.02, 2.79)**
	0.53 (0.48, 0.58)		0.54 (0.49, 0.59)		0.41 (0.36, 0.46)		0.43 (0.38, 0.48)	
Access to digital services that work	0.49 (0.45, 0.54)	1.26 (0.79, 2.02)	0.47 (0.43, 0.52)	**1.63 (1.02, 2.62)**	0.36 (0.32, 0.41)	1.56 (0.94, 2.59)	0.33 (0.28, 0.38)	**2.11 (1.27, 3.51)**
	0.52 (0.48, 0.57)		0.54 (0.50, 0.59)		0.43 (0.38, 0.47)		0.43 (0.39, 0.48)	
Digital services that suit individual needs	0.49 (0.45, 0.53)	1.24 (0.83, 1.85)	0.50 (0.46, 0.54)	1.16 (0.77, 1.75)	0.38 (0.34, 0.43)	1.23 (0.80, 1.90)	0.38 (0.34, 0.42)	1.05 (0.69, 1.58)
	0.53 (0.48, 0.57)		0.52 (0.48, 0.56)		0.41 (0.37, 0.46)		0.39 (0.35, 0.43)	

Abbreviations: PM= predicted marginals, aOR= adjusted odds ratio, CI= confidence interval, Ref= reference group, AIAN= American Indian or Alaska Native, NHPI= Native Hawaiian or Pacific Islander, AA= African American.
Logistic regression analysis modeled the probability of 1= always. Bolded cells represent significant results where the confidence interval does not include the null value, 1.
Predicted marginals are shown for top age of the 4 age brackets as they appear in Table 1, whereas predicted marginals for digital health literacy are shown for 25*th* and 75*th* percentile of the domain score.

© 2026 Tesfaye L et al. *JAMA Network Open*.

eTable 10. Factors associated with optimal patient-centered communication among those who had 1 or more telehealth visits in the past year, *N* = 2165, complete case

Characteristics	Give chance to ask questions		Give attention to feelings		Involve you in decisions	
	PM (95% CI)	aOR (95% CI)	PM (95% CI)	aOR (95% CI)	PM (95% CI)	aOR (95% CI)
**Age**						
18	0.46 (0.41, 0.51)	1.00 (0.99, 1.01)	0.43 (0.39, 0.48)	1.00 (0.99, 1.00)	0.43 (0.38, 0.48)	1.00 (0.99, 1.01)
30	0.47 (0.43, 0.50)		0.44 (0.40, 0.47)		0.45 (0.42, 0.48)	
45	0.48 (0.46, 0.50)		0.44 (0.42, 0.46)		0.47 (0.45, 0.49)	
60	0.49 (0.46, 0.53)		0.44 (0.41, 0.47)		0.49 (0.46, 0.53)	
**Sex**						
Female	0.51 (0.48, 0.53)	**1.27 (1.04, 1.55)**	0.47 (0.44, 0.50)	**1.37 (1.12, 1.67)**	0.50 (0.47, 0.53)	**1.28 (1.05, 1.57**)
Male	0.45 (0.42, 0.48)	Ref	0.40 (0.37, 0.43)	Ref	0.44 (0.41, 0.47)	Ref
**Sexual orientation**						
Heterosexual	0.48 (0.46, 0.50)	Ref	0.44 (0.42, 0.46)	Ref	0.47 (0.45, 0.50)	Ref
Gay, lesbian, bisexual	0.49 (0.43, 0.55)	1.04 (0.77, 1.40)	0.45 (0.39, 0.51)	1.05 (0.78, 1.41)	0.47 (0.41, 0.53)	0.99 (0.73, 1.34)
**Race and/or ethnicity**						
AIAN, Asian, NHPI	0.40 (0.32, 0.48)	**0.65 (0.41, 0.96)**	0.32 (0.25, 0.40)	**0.50 (0.32, 0.76)**	0.34 (0.26, 0.43)	**0.49 (0.31, 0.76)**
Black or AA	0.43 (0.38, 0.48)	**0.73 (0.55, 0.97)**	0.41 (0.36, 0.46)	0.77 (0.57, 1.03)	0.45 (0.40, 0.51)	0.85 (0.64, 1.13)
Hispanic or Latino	0.51 (0.45, 0.56)	1.04 (0.79, 1.38)	0.43 (0.37, 0.48)	0.83 (0.63, 1.10)	0.47 (0.42, 0.53)	0.93 (0.70, 1.23)
White	0.50 (0.47, 0.52)	Ref	0.46 (0.44, 0.49)	Ref	0.49 (0.46, 0.51)	Ref
**Education**						
<High school	0.52 (0.46, 0.58)	0.91 (0.80, 1.03)	0.47 (0.42, 0.53)	0.92 (0.82, 1.05)	0.57 (0.51, 0.62)	**0.80 (0.70, 0.90)**
High school graduate	0.50 (0.47, 0.54)		0.46 (0.42, 0.49)		0.52 (0.49, 0.55)	
Vocational school, some college	0.48 (0.46, 0.50)		0.44 (0.42, 0.46)		0.47 (0.45, 0.49)	
College graduate or higher	0.46 (0.43, 0.50)		0.43 (0.39, 0.46)		0.43 (0.39, 0.46)	
**Income**						
<$20,000	0.51 (0.47, 0.54)	0.92 (0.84, 1.02)	0.46 (0.42, 0.50)	0.94 (0.85, 1.04)	0.49 (0.45, 0.52)	0.96 (0.87, 1.06)
$20,000 to $49,999	0.49 (0.47, 0.51)		0.45 (0.42, 0.47)		0.48 (0.45, 0.50)	
$50,000 to $74,999	0.47 (0.45, 0.50)		0.44 (0.41, 0.46)		0.47 (0.45, 0.49)	
≥$75,000	0.46 (0.42, 0.50)		0.42 (0.39, 0.46)		0.46 (0.42, 0.50)	
**English proficiency**						

© 2026 Tesfaye L et al. *JAMA Network Open*.

Characteristics	Give chance to ask questions		Give attention to feelings		Involve you in decisions	
	PM (95% CI)	aOR (95% CI)	PM (95% CI)	aOR (95% CI)	PM (95% CI)	aOR (95% CI)
Very well	0.49 (0.47, 0.51)	Ref	0.45 (0.42, 0.47)	Ref	0.48 (0.46, 0.50)	Ref
Well, not well, not at all	0.35 (0.28, 0.43)	**0.52 (0.34, 0.77)**	0.37 (0.29, 0.45)	0.68 (0.45, 1.02)	0.37 (0.29, 0.45)	**0.58 (0.38, 0.87)**
**Health insurance**						
Insured	0.48 (0.46, 0.51)	Ref	0.44 (0.42, 0.46)	Ref	0.47 (0.45, 0.50)	Ref
Uninsured	0.44 (0.36, 0.52)	0.80 (0.53, 1.20)	0.41 (0.33, 0.50)	0.85 (0.56, 1.29)	0.45 (0.37, 0.53)	0.88 (0.59, 1.34)
**General health**						
Excellent, very good, good	0.48 (0.45, 0.50)	Ref	0.44 (0.42, 0.47)	Ref	0.47 (0.44, 0.49)	Ref
Fair, poor	0.49 (0.45, 0.54)	1.07 (0.84, 1.36)	0.43 (0.39, 0.48)	0.95 (0.74, 1.21)	0.49 (0.44, 0.53)	1.09 (0.86, 1.39)
**Mental health**						
Excellent, very good, good	0.50 (0.47, 0.52)	Ref	0.46 (0.43, 0.48)	Ref	0.49 (0.46, 0.51)	Ref
Fair, poor	0.44 (0.40, 0.48)	**0.76 (0.60, 0.96)**	0.40 (0.36, 0.44)	**0.76 (0.61, 0.96)**	0.44 (0.40, 0.48)	**0.79 (0.62, 0.99)**
**Has a primary care clinician**						
Yes	0.53 (0.50, 0.56)	Ref	0.48 (0.45, 0.51)	Ref	0.52 (0.49, 0.55)	Ref
No	0.42 (0.38, 0.45)	**0.59 (0.48, 0.72)**	0.39 (0.36, 0.42)	**0.65 (0.53, 0.79)**	0.41 (0.37, 0.44)	**0.59 (0.48, 0.72)**
**Presence of underlying clinical conditions**						
Yes	0.51 (0.48, 0.55)	**1.28 (1.03, 1.58)**	0.45 (0.41, 0.48)	1.04 (0.83, 1.29)	0.49 (0.45, 0.52)	1.13 (0.91, 1.40)
No	0.46 (0.43, 0.49)	Ref	0.44 (0.41, 0.47)	Ref	0.46 (0.43, 0.49)	Ref
**Past year in-person visit(s) with healthcare clinician**						
0 times	0.44 (0.37, 0.52)	Ref	0.41 (0.34, 0.49)	Ref	0.44 (0.37, 0.52)	Ref
1 or more times	0.49 (0.46, 0.51)	1.23 (0.85, 1.76)	0.44 (0.42, 0.46)	1.15 (0.80, 1.65)	0.48 (0.45, 0.50)	1.16 (0.81, 1.65)
**History of COVID-19 infection**						
Yes	0.50 (0.46, 0.53)	1.09 (0.89, 1.33)	0.46 (0.43, 0.50)	1.17 (0.96, 1.43)	0.51 (0.48, 0.55)	**1.31 (1.07, 1.60)**
No	0.48 (0.45, 0.50)	Ref	0.43 (0.41, 0.45)	Ref	0.45 (0.43, 0.48)	Ref
**Body mass index**						

© 2026 Tesfaye L et al. *JAMA Network Open*.

Characteristics	Give chance to ask questions		Give attention to feelings		Involve you in decisions	
	PM (95% CI)	aOR (95% CI)	PM (95% CI)	aOR (95% CI)	PM (95% CI)	aOR (95% CI)
Healthy (18.5 to <25)	0.47 (0.43, 0.51)	Ref	0.44 (0.40, 0.47)	Ref	0.47 (0.43, 0.51)	Ref
Unhealthy (<18.5 or ≥25)	0.49 (0.46, 0.51)	1.10 (0.89, 1.36)	0.44 (0.42, 0.47)	1.03 (0.83, 1.28)	0.47 (0.45, 0.50)	1.01 (0.82, 1.26)
**Physical activity**						
Sufficient (≥150 minutes per week)	0.48 (0.46, 0.51)	Ref	0.43 (0.41, 0.46)	Ref	0.47 (0.45, 0.50)	Ref
Insufficient (<150 minutes per week)	0.48 (0.44, 0.52)	0.99 (0.80, 1.22)	0.46 (0.42, 0.50)	1.14 (0.92, 1.41)	0.48 (0.44, 0.51)	1.02 (0.82, 1.26)
**Past month cigarette smoking**						
Yes	0.44 (0.40, 0.48)	**0.73 (0.58, 0.93)**	0.42 (0.38, 0.46)	0.84 (0.66, 1.06)	0.44 (0.40, 0.48)	0.80 (0.63, 1.02)
No	0.50 (0.48, 0.53)	Ref	0.45 (0.43, 0.48)	Ref	0.49 (0.46, 0.52)	Ref
**Past month e-cigarette use**						
Yes	0.46 (0.41, 0.51)	0.88 (0.67, 1.16)	0.42 (0.37, 0.47)	0.86 (0.65, 1.12)	0.45 (0.40, 0.50)	0.86 (0.65, 1.14)
No	0.49 (0.46, 0.51)	Ref	0.45 (0.42, 0.47)	Ref	0.48 (0.45, 0.50)	Ref
**Alcohol misuse**						
Yes	0.48 (0.41, 0.56)	1.01 (0.70, 1.45)	0.44 (0.37, 0.52)	1.00 (0.70, 1.45)	0.50 (0.42, 0.57)	1.13 (0.77, 1.64)
No	0.48 (0.46, 0.50)	Ref	0.44 (0.42, 0.46)	Ref	0.47 (0.45, 0.49)	Ref
Not applicable (<21 years)	0.48 (0.36, 0.59)	0.98 (0.56, 1.69)	0.47 (0.36, 0.57)	1.13 (0.67, 1.89)	0.48 (0.36, 0.59)	1.03 (0.59, 1.78)
**Past month marijuana or cannabis use**						
Yes	0.47 (0.43, 0.51)	0.93 (0.74, 1.18)	0.42 (0.38, 0.46)	0.88 (0.69, 1.12)	0.43 (0.39, 0.47)	**0.77 (0.60, 0.97)**
No	0.49 (0.46, 0.51)	Ref	0.45 (0.42, 0.47)	Ref	0.49 (0.46, 0.51)	Ref
**Device access**						
Yes	0.48 (0.46, 0.50)	Ref	0.44 (0.42, 0.46)	Ref	0.47 (0.45, 0.49)	Ref
No	0.44 (0.13, 0.79)	0.81 (0.12, 5.16)	0.32 (0.08, 0.72)	0.56 (0.07, 4.02)	0.44 (0.15, 0.77)	0.85 (0.15, 4.73)
**Internet access**						
Yes	0.48 (0.46, 0.50)	Ref	0.44 (0.42, 0.46)	Ref	0.47 (0.45, 0.49)	Ref
No	0.50 (0.32, 0.67)	1.06 (0.45, 2.47)	0.41 (0.23, 0.61)	0.84 (0.31, 2.25)	0.45 (0.26, 0.65)	0.89 (0.34, 2.31)
**Digital health literacy**						

© 2026 Tesfaye L et al. *JAMA Network Open*.

Characteristics	Give chance to ask questions		Give attention to feelings		Involve you in decisions	
	PM (95% CI)	aOR (95% CI)	PM (95% CI)	aOR (95% CI)	PM (95% CI)	aOR (95% CI)
Using technology to process health information	0.49 (0.46, 0.52)<br>0.47 (0.43, 0.50)	0.83 (0.58, 1.19)	0.45 (0.42, 0.48)<br>0.43 (0.39, 0.46)	0.82 (0.56, 1.19)	0.49 (0.46, 0.52)<br>0.45 (0.41, 0.48)	0.70 (0.49, 1.00)
Understanding of health concepts and language	0.46 (0.43, 0.49)<br>0.51 (0.47, 0.54)	**1.42 (1.01, 1.99)**	0.42 (0.39, 0.45)<br>0.47 (0.43, 0.50)	**1.45 (1.03, 2.04)**	0.45 (0.42, 0.48)<br>0.50 (0.47, 0.53)	**1.42 (1.01, 1.97)**
Ability to actively engage with digital services	0.49 (0.46, 0.52)<br>0.47 (0.44, 0.50)	0.87 (0.65, 1.17)	0.45 (0.42, 0.48)<br>0.43 (0.40, 0.46)	0.85 (0.63, 1.14)	0.46 (0.43, 0.48)<br>0.49 (0.46, 0.52)	1.31 (0.98, 1.74)
Feel safe and in control	0.46 (0.44, 0.49)<br>0.50 (0.47, 0.52)	1.25 (1.00, 1.57)	0.40 (0.37, 0.43)<br>0.47 (0.44, 0.50)	**1.69 (1.34, 2.14)**	0.44 (0.41, 0.47)<br>0.50 (0.47, 0.52)	**1.51 (1.21, 1.89)**
Motivated to engage with digital services	0.47 (0.44, 0.50)<br>0.50 (0.46, 0.54)	1.23 (0.86, 1.77)	0.43 (0.40, 0.46)<br>0.45 (0.42, 0.49)	1.19 (0.82, 1.73)	0.47 (0.44, 0.49)<br>0.48 (0.45, 0.52)	1.14 (0.79, 1.65)
Access to digital services that work	0.42 (0.38, 0.46)<br>0.53 (0.50, 0.56)	**2.02 (1.42, 2.88)**	0.39 (0.35, 0.43)<br>0.48 (0.44, 0.51)	**1.77 (1.22, 2.56)**	0.43 (0.40, 0.47)<br>0.50 (0.47, 0.53)	**1.56 (1.09, 2.25)**
Digital services that suit individual needs	0.46 (0.43, 0.50)<br>0.50 (0.47, 0.52)	1.21 (0.90, 1.61)	0.41 (0.37, 0.44)<br>0.46 (0.43, 0.49)	**1.42 (1.05, 1.92)**	0.45 (0.42, 0.49)<br>0.49 (0.46, 0.52)	1.24 (0.93, 1.67)
**MHSVI**						
Most vulnerable counties	0.49 (0.46, 0.52)	1.05 (0.86, 1.28)	0.45 (0.43, 0.48)	1.15 (0.94, 1.41)	0.48 (0.45, 0.51)	1.03 (0.84, 1.26)
Least vulnerable counties	0.48 (0.44, 0.51)	Ref	0.42 (0.39, 0.46)	Ref	0.47 (0.44, 0.50)	Ref

© 2026 Tesfaye L et al. *JAMA Network Open*.

eTable 10. Factors associated with optimal patient-centered communication among those who had 1 or more telehealth visits in the past year, *N* = 2165, complete case (continued)

**Characteristics**	**Make sure you understand**		**Explain things**		**Spend enough time**		**Help deal with uncertainty**	
	PM (95% CI)	aOR (95% CI)	PM (95% CI)	aOR (95% CI)	PM (95% CI)	aOR (95% CI)	PM (95% CI)	aOR (95% CI)
**Age**								
18	0.48 (0.43, 0.52)	1.00 (1.00, 1.01)	0.48 (0.43, 0.52)	1.00 (1.00, 1.01)	0.35 (0.31, 0.40)	1.01 (1.00, 1.01)	0.39 (0.34, 0.43)	1.00 (0.99, 1.01)
30	0.50 (0.46, 0.53)		0.50 (0.46, 0.53)		0.37 (0.34, 0.41)		0.40 (0.37, 0.43)	
45	0.52 (0.50, 0.54)		0.52 (0.50, 0.54)		0.41 (0.39, 0.43)		0.41 (0.39, 0.43)	
60	0.54 (0.51, 0.57)		0.55 (0.52, 0.58)		0.44 (0.41, 0.47)		0.43 (0.40, 0.46)	
**Sex**								
Female	0.54 (0.51, 0.56)	1.15 (0.94, 1.41)	0.55 (0.52, 0.58)	**1.31 (1.07, 1.61)**	0.43 (0.40, 0.46)	**1.27 (1.04, 1.56)**	0.43 (0.40, 0.46)	1.16 (0.95, 1.42)
Male	0.50 (0.47, 0.54)	Ref	0.49 (0.46, 0.52)	Ref	0.38 (0.35, 0.41)	Ref	0.40 (0.37, 0.43)	Ref
**Sexual orientation**								
Heterosexual	0.52 (0.50, 0.54)	Ref	0.52 (0.50, 0.54)	Ref	0.40 (0.38, 0.43)	Ref	0.41 (0.39, 0.43)	Ref
Gay, lesbian, bisexual	0.51 (0.45, 0.57)	0.95 (0.71, 1.27)	0.55 (0.49, 0.61)	1.17 (0.87, 1.57)	0.45 (0.39, 0.51)	1.24 (0.92, 1.67)	0.43 (0.37, 0.49)	1.06 (0.78, 1.44)
**Race and/or ethnicity**								
AIAN, Asian, NHPI	0.46 (0.37, 0.54)	0.71 (0.46, 1.07)	0.42 (0.34, 0.50)	**0.57 (0.37, 0.86)**	0.33 (0.25, 0.41)	**0.59 (0.38, 0.93)**	0.29 (0.22, 0.38)	**0.51 (0.33, 0.78)**
Black or AA	0.51 (0.45, 0.56)	0.90 (0.68, 1.19)	0.53 (0.48, 0.59)	0.98 (0.74, 1.30)	0.34 (0.30, 0.40)	**0.64 (0.48, 0.87)**	0.38 (0.33, 0.44)	0.81 (0.60, 1.08)
Hispanic or Latino	0.52 (0.47, 0.57)	0.96 (0.73, 1.28)	0.50 (0.45, 0.56)	0.85 (0.64, 1.14)	0.40 (0.35, 0.45)	0.85 (0.64, 1.13)	0.42 (0.37, 0.47)	0.94 (0.71, 1.25)
White	0.53 (0.50, 0.56)	Ref	0.54 (0.51, 0.56)	Ref	0.43 (0.41, 0.46)	Ref	0.43 (0.40, 0.46)	Ref
**Education**								
<High school	0.62 (0.56, 0.67)	**0.79 (0.70, 0.90)**	0.59 (0.54, 0.65)	**0.84 (0.74, 0.95)**	0.50 (0.44, 0.56)	**0.80 (0.71, 0.91)**	0.49 (0.43, 0.55)	**0.83 (0.73, 0.94)**
High school graduate	0.57 (0.54, 0.60)		0.56 (0.53, 0.59)		0.45 (0.42, 0.49)		0.45 (0.42, 0.49)	

© 2026 Tesfaye L et al. *JAMA Network Open*.

Characteristics	Make sure you understand		Explain things		Spend enough time		Help deal with uncertainty	
	PM (95% CI)	aOR (95% CI)	PM (95% CI)	aOR (95% CI)	PM (95% CI)	aOR (95% CI)	PM (95% CI)	aOR (95% CI)
Vocational school, some college	0.52 (0.50, 0.54)		0.52 (0.50, 0.54)		0.41 (0.39, 0.43)		0.41 (0.39, 0.43)	
College graduate or higher	0.47 (0.44, 0.51)		0.49 (0.45, 0.52)		0.37 (0.33, 0.40)		0.38 (0.34, 0.41)	
**Income**								
<$20,000	0.55 (0.51, 0.58)	0.92 (0.84, 1.02)	0.53 (0.49, 0.57)	0.97 (0.88, 1.07)	0.42 (0.38, 0.46)	0.96 (0.87, 1.06)	0.45 (0.42, 0.49)	**0.88 (0.80, 0.97)**
$20,000 to $49,999	0.53 (0.51, 0.55)		0.53 (0.50, 0.55)		0.41 (0.39, 0.44)		0.43 (0.40, 0.45)	
$50,000 to $74,999	0.51 (0.49, 0.54)		0.52 (0.50, 0.54)		0.41 (0.38, 0.43)		0.40 (0.38, 0.42)	
≥$75,000	0.50 (0.46, 0.53)		0.52 (0.48, 0.55)		0.40 (0.36, 0.44)		0.37 (0.34, 0.41)	
**English proficiency**								
Very well	0.53 (0.51, 0.55)	Ref	0.53 (0.51, 0.55)	Ref	0.41 (0.39, 0.44)	Ref	0.42 (0.40, 0.44)	Ref
Well, not well, not at all	0.39 (0.31, 0.47)	**0.50 (0.34, 0.76)**	0.39 (0.31, 0.48)	**0.52 (0.33, 0.80)**	0.32 (0.25, 0.40)	**0.63 (0.41, 0.95)**	0.31 (0.24, 0.40)	**0.59 (0.39, 0.89)**
**Health insurance**								
Insured	0.52 (0.50, 0.55)	Ref	0.53 (0.50, 0.55)	Ref	0.41 (0.39, 0.43)	Ref	0.42 (0.40, 0.44)	Ref
Uninsured	0.48 (0.39, 0.56)	0.80 (0.53, 1.20)	0.49 (0.41, 0.58)	0.86 (0.57, 1.30)	0.42 (0.34, 0.50)	1.04 (0.69, 1.57)	0.33 (0.26, 0.42)	**0.65 (0.43, 0.99)**
**General health**								
Excellent, very good, good	0.51 (0.49, 0.54)	Ref	0.52 (0.50, 0.55)	Ref	0.41 (0.38, 0.43)	Ref	0.42 (0.39, 0.44)	Ref
Fair, poor	0.54 (0.50, 0.59)	1.15 (0.90, 1.47)	0.53 (0.48, 0.57)	1.02 (0.80, 1.30)	0.41 (0.37, 0.45)	1.00 (0.78, 1.28)	0.40 (0.35, 0.44)	0.89 (0.69, 1.13)
**Mental health**								
Excellent, very good, good	0.55 (0.52, 0.57)	Ref	0.54 (0.52, 0.57)	Ref	0.44 (0.41, 0.46)	Ref	0.44 (0.41, 0.46)	Ref
Fair, poor	0.46 (0.42, 0.51)	**0.69 (0.54, 0.87)**	0.48 (0.44, 0.52)	**0.74 (0.58, 0.93)**	0.35 (0.31, 0.39)	**0.64 (0.51, 0.82)**	0.35 (0.32, 0.39)	**0.66 (0.53, 0.84)**

© 2026 Tesfaye L et al. *JAMA Network Open*.

Characteristics	Make sure you understand		Explain things		Spend enough time		Help deal with uncertainty	
	PM (95% CI)	aOR (95% CI)	PM (95% CI)	aOR (95% CI)	PM (95% CI)	aOR (95% CI)	PM (95% CI)	aOR (95% CI)
**Has a primary care clinician**								
Yes	0.57 (0.54, 0.60)	Ref	0.56 (0.53, 0.59)	Ref	0.44 (0.42, 0.47)	Ref	0.44 (0.42, 0.47)	Ref
No	0.45 (0.42, 0.49)	**0.58 (0.48, 0.71)**	0.47 (0.44, 0.51)	**0.66 (0.53, 0.81)**	0.36 (0.33, 0.39)	**0.67 (0.54, 0.82)**	0.37 (0.34, 0.40)	**0.70 (0.57, 0.86)**
**Presence of underlying clinical conditions**								
Yes	0.53 (0.49, 0.56)	1.04 (0.84, 1.29)	0.55 (0.52, 0.59)	1.24 (1.00, 1.55)	0.43 (0.39, 0.46)	1.15 (0.93, 1.44)	0.42 (0.39, 0.46)	1.07 (0.86, 1.33)
No	0.52 (0.49, 0.55)	Ref	0.50 (0.48, 0.53)	Ref	0.40 (0.37, 0.42)	Ref	0.41 (0.38, 0.44)	Ref
**Past year in-person visit(s) with healthcare clinician**								
0 times	0.50 (0.43, 0.58)	Ref	0.50 (0.43, 0.57)	Ref	0.39 (0.32, 0.46)	Ref	0.39 (0.32, 0.47)	Ref
1 or more times	0.52 (0.50, 0.54)	1.08 (0.76, 1.54)	0.53 (0.50, 0.55)	1.13 (0.80, 1.61)	0.41 (0.39, 0.43)	1.11 (0.77, 1.59)	0.41 (0.39, 0.44)	1.12 (0.77, 1.61)
**History of COVID-19 infection**								
Yes	0.54 (0.50, 0.58)	1.13 (0.92, 1.38)	0.54 (0.51, 0.58)	1.13 (0.92, 1.39)	0.44 (0.40, 0.47)	1.22 (1.00, 1.50)	0.44 (0.41, 0.48)	1.20 (0.99, 1.47)
No	0.51 (0.49, 0.54)	Ref	0.52 (0.49, 0.54)	Ref	0.40 (0.37, 0.42)	Ref	0.40 (0.38, 0.43)	Ref
**Body mass index**								
Healthy (18.5 to <25)	0.50 (0.47, 0.54)	Ref	0.51 (0.47, 0.55)	Ref	0.42 (0.39, 0.46)	Ref	0.40 (0.37, 0.44)	Ref
Unhealthy (<18.5 or ≥25)	0.53 (0.50, 0.55)	1.11 (0.90, 1.37)	0.53 (0.50, 0.55)	1.09 (0.88, 1.35)	0.40 (0.38, 0.43)	0.90 (0.72, 1.11)	0.42 (0.39, 0.44)	1.06 (0.85, 1.31)
**Physical activity**								
Sufficient (≥150 minutes per week)	0.53 (0.50, 0.55)	Ref	0.51 (0.49, 0.54)	Ref	0.40 (0.38, 0.43)	Ref	0.41 (0.38, 0.43)	Ref

© 2026 Tesfaye L et al. *JAMA Network Open*.

Characteristics	Make sure you understand		Explain things		Spend enough time		Help deal with uncertainty	
	PM (95% CI)	aOR (95% CI)	PM (95% CI)	aOR (95% CI)	PM (95% CI)	aOR (95% CI)	PM (95% CI)	aOR (95% CI)
Insufficient (<150 minutes per week)	0.51 (0.47, 0.55)	0.92 (0.75, 1.14)	0.54 (0.51, 0.58)	1.15 (0.93, 1.42)	0.43 (0.39, 0.46)	1.12 (0.90, 1.39)	0.43 (0.39, 0.46)	1.09 (0.88, 1.35)
**Past month cigarette smoking**								
Yes	0.48 (0.44, 0.52)	**0.73 (0.57, 0.92)**	0.49 (0.45, 0.53)	0.80 (0.63, 1.02)	0.40 (0.36, 0.44)	0.95 (0.75, 1.22)	0.38 (0.34, 0.42)	0.80 (0.62, 1.01)
No	0.54 (0.52, 0.57)	Ref	0.54 (0.51, 0.57)	Ref	0.41 (0.39, 0.44)	Ref	0.43 (0.40, 0.46)	Ref
**Past month e-cigarette use**								
Yes	0.50 (0.45, 0.55)	0.87 (0.66, 1.14)	0.50 (0.45, 0.55)	0.85 (0.65, 1.12)	0.40 (0.35, 0.45)	0.94 (0.71, 1.25)	0.43 (0.38, 0.49)	1.13 (0.85, 1.49)
No	0.53 (0.50, 0.55)	Ref	0.53 (0.51, 0.55)	Ref	0.41 (0.39, 0.44)	Ref	0.41 (0.38, 0.43)	Ref
**Alcohol misuse**								
Yes	0.51 (0.43, 0.58)	0.92 (0.64, 1.32)	0.52 (0.44, 0.60)	0.97 (0.67, 1.41)	0.39 (0.32, 0.46)	0.89 (0.61, 1.30)	0.38 (0.30, 0.45)	0.83 (0.56, 1.21)
No	0.53 (0.50, 0.55)	Ref	0.53 (0.50, 0.55)	Ref	0.41 (0.39, 0.43)	Ref	0.41 (0.39, 0.44)	Ref
Not applicable (<21 years of age)	0.43 (0.32, 0.55)	0.64 (0.37, 1.11)	0.44 (0.33, 0.56)	0.67 (0.38, 1.18)	0.42 (0.32, 0.54)	1.06 (0.61, 1.83)	0.45 (0.34, 0.57)	1.19 (0.68, 2.08)
**Past month marijuana or cannabis use**								
Yes	0.49 (0.45, 0.54)	0.84 (0.66, 1.06)	0.48 (0.43, 0.52)	**0.73 (0.57, 0.92)**	0.36 (0.32, 0.40)	**0.73 (0.57, 0.93)**	0.38 (0.34, 0.42)	0.81 (0.64, 1.03)
No	0.53 (0.51, 0.56)	Ref	0.54 (0.52, 0.57)	Ref	0.43 (0.40, 0.45)	Ref	0.43 (0.40, 0.45)	Ref
**Device access**								
Yes	0.52 (0.50, 0.54)	Ref	0.52 (0.50, 0.54)	Ref	0.41 (0.39, 0.43)	Ref	0.41 (0.39, 0.43)	Ref
No	0.59 (0.25, 0.86)	1.39 (0.26, 7.43)	0.66 (0.32, 0.88)	1.91 (0.37, 9.68)	0.44 (0.14, 0.78)	1.13 (0.18, 6.86)	0.47 (0.13, 0.83)	1.29 (0.16, 9.87)
**Internet access**								
Yes	0.52 (0.50, 0.54)	Ref	0.52 (0.50, 0.54)	Ref	0.41 (0.39, 0.43)	Ref	0.41 (0.39, 0.43)	Ref

© 2026 Tesfaye L et al. *JAMA Network Open*.

Characteristics	Make sure you understand		Explain things		Spend enough time		Help deal with uncertainty	
	PM (95% CI)	aOR (95% CI)	PM (95% CI)	aOR (95% CI)	PM (95% CI)	aOR (95% CI)	PM (95% CI)	aOR (95% CI)
No	0.54 (0.34, 0.73)	1.09 (0.42, 2.85)	0.59 (0.39, 0.76)	1.36 (0.53, 3.46)	0.40 (0.23, 0.59)	0.94 (0.37, 2.41)	0.50 (0.32, 0.68)	1.47 (0.61, 3.55)
**Digital health literacy**								
Using technology to process health information	0.53 (0.50 0.56)	0.81 (0.57, 1.15)	0.55 (0.52, 0.57)	**0.55 (0.38, 0.79)**	0.42 (0.40, 0.45)	0.75 (0.52, 1.09)	0.44 (0.41, 0.47)	**0.62 (0.43, 0.91)**
	0.50 (0.47, 0.54)		0.48 (0.45, 0.51)		0.39 (0.36, 0.42)		0.38 (0.35, 0.41)	
Understanding of health concepts and language	0.50 (0.46, 0.53)	**1.52 (1.09, 2.13)**	0.47 (0.44, 0.50)	**2.49 (1.76, 3.52)**	0.38 (0.35, 0.41)	**1.59 (1.12, 2.25)**	0.38 (0.35, 0.41)	**1.67 (1.19, 2.36)**
	0.55 (0.52, 0.58)		0.59 (0.56, 0.62)		0.44 (0.41, 0.47)		0.45 (0.42, 0.48)	
Ability to actively engage with digital services	0.52 (0.50 0.55)	1.00 (0.75, 1.33)	0.51 (0.49, 0.54)	1.22 (0.90, 1.64)	0.42 (0.39, 0.45)	0.85 (0.63, 1.15)	0.43 (0.40, 0.45)	0.77 (0.57, 1.04)
	0.52 (0.49, 0.55)		0.54 (0.51, 0.57)		0.40 (0.37, 0.43)		0.40 (0.37, 0.42)	
Feel safe and in control	0.50 (0.47, 0.52)	**1.40 (1.12, 1.74)**	0.51 (0.48, 0.53)	1.29 (1.03, 1.62)	0.36 (0.34, 0.39)	**1.77 (1.38, 2.26)**	0.38 (0.35, 0.41)	**1.54 (1.22, 1.95)**
	0.54 (0.52, 0.57)		0.54 (0.51, 0.56)		0.44 (0.41, 0.46)		0.44 (0.41, 0.46)	
Motivated to engage with digital services	0.52 (0.49, 0.55)	1.05 (0.74, 1.51)	0.51 (0.48, 0.54)	1.26 (0.88, 1.82)	0.40 (0.38, 0.43)	1.07 (0.74, 1.57)	0.40 (0.37, 0.43)	1.37 (0.95, 2.00)
	0.53 (0.49, 0.56)		0.54 (0.51, 0.58)		0.41 (0.38, 0.45)		0.44 (0.40, 0.47)	
Access to digital services that work	0.49 (0.45, 0.52)	**1.50 (1.06, 2.14)**	0.50 (0.46, 0.53)	1.38 (0.96, 1.96)	0.38 (0.34, 0.42)	1.45 (1.00, 2.10)	0.37 (0.33, 0.41)	**1.69 (1.15, 2.47)**
	0.55 (0.52 0.58)		0.54 (0.51, 0.58)		0.43 (0.40, 0.46)		0.44 (0.41, 0.47)	
Digital services that suit individual needs	0.49 (0.45, 0.52)	**1.43 (1.08, 1.90)**	0.50 (0.47, 0.54)	1.27 (0.95, 1.71)	0.36 (0.33, 0.40)	**1.59 (1.18, 2.15)**	0.38 (0.34, 0.41)	**1.44 (1.07, 1.95)**
	0.55 (0.52, 0.58)		0.54 (0.51 0.57)		0.44 (0.41, 0.47)		0.44 (0.41, 0.47)	

Characteristics	Make sure you understand		Explain things		Spend enough time		Help deal with uncertainty	
	PM (95% CI)	aOR (95% CI)	PM (95% CI)	aOR (95% CI)	PM (95% CI)	aOR (95% CI)	PM (95% CI)	aOR (95% CI)
**MHSVI**								
Most vulnerable counties	0.51 (0.48, 0.54)	0.92 (0.75, 1.12)	0.52 (0.49, 0.55)	0.97 (0.79, 1.19)	0.40 (0.38, 0.43)	0.95 (0.77, 1.17)	0.42 (0.40, 0.45)	1.12 (0.91, 1.37)
Least vulnerable counties	0.53 (0.50, 0.56)	Ref	0.53 (0.49, 0.56)	Ref	0.41 (0.38, 0.45)	Ref	0.40 (0.37, 0.43)	Ref

Abbreviations: MHSVI= Minority Health Social Vulnerability Index, PM= predicted marginals, aOR= adjusted odds ratio, CI= confidence interval, Ref= reference group, AIAN= American Indian or Alaska Native, NHPI= Native Hawaiian or Pacific Islander, AA= African American.
Logistic regression analysis modeled the probability of 1= always. Bolded cells represent significant results where the confidence interval does not include the null value, 1.
Predicted marginals are shown for top age of the 4 age brackets as they appear in Table 1, whereas predicted marginals for digital health literacy are shown for 25*th* and 75*th* percentile of the domain score.

© 2026 Tesfaye L et al. *JAMA Network Open*.

eTable 11. Factors associated with optimal patient-centered communication among those who had 1 or more telehealth visits in the past year and resided in MHSVI most vulnerable counties, *n* = 1165, complete case

Characteristics	Give chance to ask questions		Give attention to feelings		Involve you in decisions	
	PM (95% CI)	aOR (95% CI)	PM (95% CI)	aOR (95% CI)	PM (95% CI)	aOR (95% CI)
**Age**						
18	0.48 (0.42, 0.55)	0.99 (0.98, 1.01)	0.47 (0.41, 0.54)	0.99 (0.98, 1.00)	0.46 (0.40, 0.53)	1.00 (0.99, 1.01)
30	0.48 (0.44, 0.52)		0.46 (0.42, 0.50)		0.47 (0.42, 0.51)	
45	0.48 (0.45, 0.51)		0.45 (0.42, 0.48)		0.47 (0.44, 0.50)	
60	0.48 (0.43, 0.52)		0.44 (0.39, 0.48)		0.48 (0.43, 0.53)	
**Sex**						
Female	0.50 (0.46, 0.54)	1.21 (0.92, 1.59)	0.48 (0.44, 0.52)	**1.39 (1.06, 1.83)**	0.48 (0.45, 0.52)	1.15 (0.87, 1.52)
Male	0.45 (0.41, 0.50)	Ref	0.41 (0.37, 0.45)	Ref	0.45 (0.41, 0.50)	Ref
**Sexual orientation**						
Heterosexual	0.48 (0.45, 0.51)	Ref	0.45 (0.42, 0.48)	Ref	0.47 (0.44, 0.50)	Ref
Gay, lesbian, bisexual	0.48 (0.40, 0.57)	1.01 (0.67, 1.54)	0.46 (0.38, 0.54)	1.03 (0.68, 1.55)	0.46 (0.37, 0.55)	0.94 (0.61, 1.43)
**Race and/or ethnicity**						
AIAN, Asian, NHPI	0.45 (0.34, 0.57)	0.78 (0.44, 1.39)	0.35 (0.25, 0.47)	**0.54 (0.31, 0.97)**	0.35 (0.25, 0.46)	**0.51 (0.29, 0.91)**
Black or AA	0.42 (0.36, 0.48)	**0.67 (0.48, 0.95)**	0.42 (0.37, 0.48)	0.77 (0.54, 1.08)	0.44 (0.38, 0.50)	0.79 (0.57, 1.11)
Hispanic or Latino	0.49 (0.44, 0.55)	0.96 (0.68, 1.35)	0.44 (0.38, 0.50)	0.83 (0.59, 1.17)	0.49 (0.43, 0.55)	0.97 (0.69, 1.37)
White	0.50 (0.46, 0.54)	Ref	0.48 (0.44, 0.52)	Ref	0.49 (0.45, 0.53)	Ref
**Education**						
<High school	0.55 (0.47, 0.62)	0.85 (0.72, 1.00)	0.50 (0.43, 0.57)	0.89 (0.75, 1.04)	0.57 (0.50, 0.64)	**0.78 (0.66, 0.91)**
High school graduate	0.51 (0.47, 0.55)		0.47 (0.43, 0.51)		0.52 (0.48, 0.56)	
Vocational school, some college	0.48 (0.45, 0.50)		0.45 (0.42, 0.48)		0.47 (0.44, 0.49)	
College graduate or higher	0.44 (0.39, 0.49)		0.42 (0.38, 0.47)		0.41 (0.37, 0.46)	
**Income**						
<$20,000	0.51 (0.46, 0.55)	0.90 (0.79, 1.02)	0.46 (0.42, 0.51)	0.95 (0.84, 1.09)	0.47 (0.42, 0.52)	1.00 (0.88, 1.14)
$20,000 to $49,999	0.49 (0.46, 0.52)		0.45 (0.42, 0.48)		0.47 (0.44, 0.50)	
$50,000 to $74,999	0.46 (0.43, 0.50)		0.44 (0.41, 0.48)		0.47 (0.44, 0.51)	
≥$75,000	0.44 (0.39, 0.50)		0.44 (0.38, 0.49)		0.47 (0.42, 0.53)	
**English proficiency**						
Very well	0.48 (0.45, 0.51)	Ref	0.45 (0.42, 0.48)	Ref	0.48 (0.45, 0.51)	Ref

© 2026 Tesfaye L et al. *JAMA Network Open*.

Characteristics	Give chance to ask questions		Give attention to feelings		Involve you in decisions	
	PM (95% CI)	aOR (95% CI)	PM (95% CI)	aOR (95% CI)	PM (95% CI)	aOR (95% CI)
Well, not well, not at all	0.42 (0.32, 0.53)	0.75 (0.44, 1.25)	0.42 (0.32, 0.52)	0.83 (0.50, 1.39)	0.41 (0.31, 0.52)	0.73 (0.43, 1.22)
**Health insurance**						
Insured	0.49 (0.46, 0.52)	Ref	0.46 (0.43, 0.49)	Ref	0.48 (0.45, 0.51)	Ref
Uninsured	0.40 (0.32, 0.50)	0.68 (0.43, 1.07)	0.40 (0.32, 0.50)	0.78 (0.49, 1.23)	0.43 (0.34, 0.52)	0.79 (0.50, 1.25)
**General health**						
Excellent, very good, good	0.48 (0.45, 0.52)	Ref	0.46 (0.42, 0.49)	Ref	0.48 (0.44, 0.51)	Ref
Fair, poor	0.47 (0.41, 0.53)	0.92 (0.66, 1.28)	0.44 (0.38, 0.50)	0.92 (0.66, 1.28)	0.46 (0.40, 0.52)	0.92 (0.66, 1.28)
**Mental health**						
Excellent, very good, good	0.50 (0.46, 0.53)	Ref	0.47 (0.43, 0.50)	Ref	0.48 (0.45, 0.52)	Ref
Fair, poor	0.44 (0.39, 0.50)	0.78 (0.57, 1.07)	0.41 (0.36, 0.46)	0.76 (0.56, 1.03)	0.45 (0.39, 0.50)	0.84 (0.62, 1.15)
**Has a primary care clinician**						
Yes	0.53 (0.49, 0.57)	Ref	0.49 (0.45, 0.53)	Ref	0.52 (0.48, 0.56)	Ref
No	0.42 (0.37, 0.46)	**0.59 (0.45, 0.78)**	0.41 (0.36, 0.45)	**0.68 (0.51, 0.90)**	0.41 (0.37, 0.45)	**0.59 (0.45, 0.77)**
**Presence of underlying clinical conditions**						
Yes	0.53 (0.48, 0.58)	**1.39 (1.03, 1.88)**	0.45 (0.40, 0.50)	0.96 (0.71, 1.31)	0.50 (0.45, 0.55)	1.25 (0.92, 1.69)
No	0.45 (0.41, 0.49)	Ref	0.45 (0.42, 0.49)	Ref	0.45 (0.42, 0.49)	Ref
**Past year in-person visit(s) with healthcare clinician**						
0 times	0.49 (0.39, 0.59)	Ref	0.46 (0.36, 0.57)	Ref	0.48 (0.37, 0.58)	Ref
1 or more times	0.48 (0.45, 0.51)	0.95 (0.57, 1.56)	0.45 (0.42, 0.48)	0.95 (0.57, 1.58)	0.47 (0.44, 0.50)	0.97 (0.58, 1.59)
**History of COVID-19 infection**						
Yes	0.50 (0.45, 0.54)	1.11 (0.85, 1.45)	0.46 (0.41, 0.51)	1.07 (0.82, 1.40)	0.49 (0.44, 0.54)	1.12 (0.86, 1.47)
No	0.47 (0.44, 0.51)	Ref	0.45 (0.41, 0.48)	Ref	0.46 (0.43, 0.50)	Ref
**Body mass index**						
Healthy (18.5 to <25)	0.46 (0.40, 0.51)	Ref	0.44 (0.39, 0.50)	Ref	0.47 (0.42, 0.53)	Ref

© 2026 Tesfaye L et al. *JAMA Network Open*.

Characteristics	Give chance to ask questions		Give attention to feelings		Involve you in decisions	
	PM (95% CI)	aOR (95% CI)	PM (95% CI)	aOR (95% CI)	PM (95% CI)	aOR (95% CI)
Unhealthy (<18.5 or ≥25)	0.49 (0.45, 0.52)	1.13 (0.84, 1.52)	0.45 (0.42, 0.49)	1.06 (0.79, 1.42)	0.47 (0.44, 0.50)	0.98 (0.73, 1.32)
**Physical activity**						
Sufficient (≥150 minutes per week)	0.47 (0.43, 0.50)	Ref	0.44 (0.40, 0.47)	Ref	0.46 (0.43, 0.49)	Ref
Insufficient (<150 minutes per week)	0.51 (0.45, 0.56)	1.18 (0.88, 1.59)	0.48 (0.43, 0.54)	1.24 (0.92, 1.67)	0.50 (0.44, 0.55)	1.18 (0.88, 1.59)
**Past month cigarette smoking**						
Yes	0.44 (0.39, 0.49)	**0.72 (0.53, 0.98)**	0.41 (0.37, 0.46)	**0.73 (0.54, 0.99)**	0.42 (0.37, 0.47)	**0.67 (0.49, 0.91)**
No	0.51 (0.47, 0.55)	Ref	0.48 (0.44, 0.52)	Ref	0.51 (0.47, 0.55)	Ref
**Past month e-cigarette use**						
Yes	0.48 (0.41, 0.54)	0.98 (0.69, 1.40)	0.42 (0.36, 0.48)	0.82 (0.58, 1.17)	0.44 (0.37, 0.50)	0.80 (0.56, 1.15)
No	0.48 (0.45, 0.51)	Ref	0.46 (0.43, 0.50)	Ref	0.48 (0.45, 0.52)	Ref
**Alcohol misuse**						
Yes	0.53 (0.42, 0.62)	1.24 (0.76, 2.01)	0.50 (0.40, 0.60)	1.29 (0.79, 2.11)	0.50 (0.39, 0.60)	1.12 (0.68, 1.86)
No	0.48 (0.45, 0.51)	Ref	0.45 (0.42, 0.48)	Ref	0.47 (0.44, 0.50)	Ref
Not applicable (<21 years)	0.43 (0.29, 0.58)	0.79 (0.38, 1.64)	0.43 (0.30, 0.57)	0.91 (0.46, 1.81)	0.42 (0.28, 0.58)	0.79 (0.38, 1.65)
**Past month marijuana or cannabis use**						
Yes	0.47 (0.42, 0.53)	0.95 (0.70, 1.30)	0.43 (0.38, 0.49)	0.87 (0.63, 1.20)	0.45 (0.39, 0.50)	0.84 (0.61, 1.15)
No	0.48 (0.45, 0.52)	Ref	0.46 (0.42, 0.50)	Ref	0.48 (0.45, 0.52)	Ref
**Device access**						
Yes	0.48 (0.45, 0.51)	Ref	0.45 (0.42, 0.48)	Ref	0.47 (0.44, 0.50)	Ref
No	0.42 (0.13, 0.78)	0.77 (0.12, 4.79)	0.29 (0.06, 0.69)	0.44 (0.06, 3.17)	0.44 (0.15, 0.76)	0.84 (0.16, 4.43)
**Internet access**						
Yes	0.48 (0.45, 0.51)	Ref	0.45 (0.42, 0.48)	Ref	0.47 (0.44, 0.50)	Ref
No	0.45 (0.26, 0.66)	0.89 (0.33, 2.39)	0.42 (0.21, 0.66)	0.84 (0.26, 2.67)	0.44 (0.23, 0.67)	0.84 (0.27, 2.54)
**Digital health literacy**						

© 2026 Tesfaye L et al. *JAMA Network Open*.

Characteristics	Give chance to ask questions		Give attention to feelings		Involve you in decisions	
	PM (95% CI)	aOR (95% CI)	PM (95% CI)	aOR (95% CI)	PM (95% CI)	aOR (95% CI)
Using technology to process health information	0.49 (0.45, 0.53)	0.82 (0.51, 1.32)	0.46 (0.42, 0.50)	0.86 (0.53, 1.41)	0.49 (0.45, 0.53)	0.72 (0.45, 1.16)
	0.47 (0.42, 0.51)		0.44 (0.40, 0.49)		0.45 (0.41, 0.49)	
Understanding of health concepts and language	0.45 (0.41, 0.50)	1.49 (0.93, 2.38)	0.43 (0.38, 0.47)	1.45 (0.90, 2.31)	0.45 (0.40, 0.49)	1.48 (0.93, 2.35)
	0.51 (0.46, 0.55)		0.48 (0.43, 0.52)		0.50 (0.46, 0.54)	
Ability to actively engage with digital services	0.50 (0.46, 0.53)	0.75 (0.50, 1.12)	0.47 (0.43, 0.50)	0.76 (0.51, 1.14)	0.45 (0.42, 0.49)	1.32 (0.89, 1.95)
	0.46 (0.42, 0.50)		0.43 (0.40, 0.47)		0.49 (0.45, 0.53)	
Feel safe and in control	0.48 (0.44, 0.52)	1.00 (0.72, 1.38)	0.40 (0.36, 0.44)	**1.78 (1.27, 2.48)**	0.44 (0.40, 0.48)	**1.47 (1.08, 2.01)**
	0.48 (0.45, 0.51)		0.48 (0.45, 0.51)		0.49 (0.46, 0.53)	
Motivated to engage with digital services	0.47 (0.43, 0.51)	1.23 (0.74, 2.03)	0.44 (0.40, 0.49)	1.12 (0.67, 1.87)	0.47 (0.42, 0.51)	1.09 (0.65, 1.83)
	0.50 (0.45, 0.54)		0.46 (0.41, 0.51)		0.48 (0.43, 0.53)	
Access to digital services that work	0.40 (0.35, 0.45)	**2.49 (1.53, 4.04)**	0.42 (0.36, 0.47)	1.44 (0.87, 2.39)	0.45 (0.40, 0.51)	1.25 (0.75, 2.10)
	0.54 (0.49, 0.58)		0.47 (0.43, 0.51)		0.49 (0.44, 0.53)	
Digital services that suit individual needs	0.46 (0.41, 0.51)	1.24 (0.85, 1.82)	0.40 (0.34, 0.45)	**1.63 (1.09, 2.44)**	0.44 (0.39, 0.49)	1.34 (0.91, 1.96)
	0.49 (0.46, 0.53)		0.48 (0.44, 0.52)		0.49 (0.45, 0.53)	

© 2026 Tesfaye L et al. *JAMA Network Open*.

eTable 11. Factors associated with optimal patient-centered communication among those who had 1 or more telehealth visits in the past year and resided in MHSVI most vulnerable counties, *n* = 1165, complete case (continued)

Characteristics	Make sure you understand		Explain things		Spend enough time		Help deal with uncertainty	
	PM (95% CI)	aOR (95% CI)	PM (95% CI)	aOR (95% CI)	PM (95% CI)	aOR (95% CI)	PM (95% CI)	aOR (95% CI)
**Age**								
18	0.53 (0.47, 0.60)	0.99 (0.98, 1.00)	0.52 (0.46, 0.58)	0.99 (0.98, 1.00)	0.37 (0.31, 0.44)	1.00 (0.99, 1.01)	0.42 (0.36, 0.49)	1.00 (0.99, 1.01)
30	0.52 (0.48, 0.56)		0.52 (0.47, 0.56)		0.38 (0.34, 0.43)		0.42 (0.38, 0.47)	
45	0.51 (0.48, 0.54)		0.51 (0.48, 0.54)		0.40 (0.37, 0.43)		0.43 (0.40, 0.45)	
60	0.49 (0.45, 0.54)		0.51 (0.46, 0.55)		0.41 (0.37, 0.46)		0.43 (0.38, 0.47)	
**Sex**								
Female	0.51 (0.47, 0.54)	0.95 (0.72, 1.26)	0.54 (0.50, 0.57)	1.29 (0.97, 1.71)	0.42 (0.39, 0.46)	**1.35 (1.02, 1.79)**	0.44 (0.40, 0.48)	1.20 (0.91, 1.58)
Male	0.52 (0.47, 0.56)	Ref	0.48 (0.44, 0.53)	Ref	0.36 (0.32, 0.41)	Ref	0.40 (0.36, 0.45)	Ref
**Sexual orientation**								
Heterosexual	0.51 (0.48, 0.54)	Ref	0.51 (0.48, 0.54)	Ref	0.40 (0.37, 0.43)	Ref	0.42 (0.39, 0.46)	Ref
Gay, lesbian, bisexual	0.52 (0.44, 0.60)	1.04 (0.69, 1.58)	0.56 (0.48, 0.64)	1.30 (0.85, 1.99)	0.41 (0.33, 0.49)	1.06 (0.70, 1.60)	0.43 (0.35, 0.51)	1.01 (0.66, 1.54)
**Race and/or ethnicity**								
AIAN, Asian, NHPI	0.49 (0.37, 0.60)	0.81 (0.45, 1.46)	0.45 (0.34, 0.57)	0.66 (0.36, 1.19)	0.42 (0.31, 0.53)	0.95 (0.54, 1.66)	0.35 (0.25, 0.46)	0.61 (0.35, 1.07)
Black or AA	0.47 (0.41, 0.53)	0.75 (0.54, 1.06)	0.50 (0.44, 0.55)	0.81 (0.57, 1.13)	0.34 (0.29, 0.40)	**0.64 (0.45, 0.92)**	0.38 (0.33, 0.44)	0.72 (0.51, 1.01)
Hispanic or Latino	0.51 (0.45, 0.57)	0.90 (0.63, 1.27)	0.48 (0.42, 0.54)	0.75 (0.53, 1.08)	0.39 (0.34, 0.45)	0.85 (0.60, 1.20)	0.43 (0.37, 0.49)	0.91 (0.65, 1.28)
White	0.53 (0.49, 0.57)	Ref	0.54 (0.50, 0.58)	Ref	0.43 (0.38, 0.47)	Ref	0.45 (0.41, 0.49)	Ref
**Education**								
<High school	0.61 (0.54, 0.67)	**0.78 (0.66, 0.92)**	0.58 (0.51, 0.64)	**0.84 (0.71, 0.99)**	0.50 (0.42, 0.57)	**0.78 (0.66, 0.92)**	0.51 (0.44, 0.58)	**0.81 (0.69, 0.95)**

© 2026 Tesfaye L et al. *JAMA Network Open*.

Characteristics	Make sure you understand		Explain things		Spend enough time		Help deal with uncertainty	
	PM (95% CI)	aOR (95% CI)	PM (95% CI)	aOR (95% CI)	PM (95% CI)	aOR (95% CI)	PM (95% CI)	aOR (95% CI)
High school graduate	0.56 (0.51, 0.60)		0.54 (0.50, 0.58)		0.44 (0.40, 0.49)		0.46 (0.42, 0.51)	
Vocational school, some college	0.50 (0.48, 0.53)		0.51 (0.48, 0.54)		0.39 (0.36, 0.42)		0.42 (0.39, 0.45)	
College graduate or higher	0.45 (0.40, 0.50)		0.47 (0.43, 0.52)		0.34 (0.30, 0.39)		0.38 (0.33, 0.42)	
**Income**								
<$20,000	0.53 (0.49, 0.58)	0.91 (0.80, 1.04)	0.52 (0.47, 0.56)	0.97 (0.85, 1.11)	0.40 (0.36, 0.45)	0.97 (0.84, 1.11)	0.47 (0.42, 0.52)	**0.85 (0.74, 0.97)**
$20,000 to $49,999	0.52 (0.49, 0.55)		0.51 (0.48, 0.54)		0.40 (0.37, 0.43)		0.43 (0.41, 0.46)	
$50,000 to $74,999	0.50 (0.46, 0.53)		0.51 (0.47, 0.54)		0.39 (0.36, 0.43)		0.40 (0.37, 0.44)	
≥$75,000	0.48 (0.42, 0.53)		0.50 (0.45, 0.56)		0.39 (0.33, 0.44)		0.37 (0.31, 0.42)	
**English proficiency**								
Very well	0.52 (0.49, 0.55)	Ref	0.52 (0.49, 0.55)	Ref	0.40 (0.37, 0.43)	Ref	0.43 (0.40, 0.46)	Ref
Well, not well, not at all	0.40 (0.30, 0.51)	**0.57 (0.34, 0.95)**	0.46 (0.35, 0.58)	0.77 (0.44, 1.36)	0.34 (0.25, 0.44)	0.72 (0.42, 1.22)	0.37 (0.27, 0.47)	0.73 (0.44, 1.23)
**Health insurance**								
Insured	0.52 (0.49, 0.55)	Ref	0.52 (0.49, 0.55)	Ref	0.40 (0.37, 0.43)	Ref	0.44 (0.41, 0.47)	Ref
Uninsured	0.43 (0.34, 0.53)	0.67 (0.43, 1.06)	0.46 (0.37, 0.56)	0.77 (0.48, 1.23)	0.42 (0.33, 0.51)	1.09 (0.69, 1.74)	0.33 (0.25, 0.42)	**0.59 (0.37, 0.95)**
**General health**								
Excellent, very good, good	0.51 (0.47, 0.54)	Ref	0.52 (0.48, 0.55)	Ref	0.41 (0.37, 0.44)	Ref	0.44 (0.41, 0.48)	Ref
Fair, poor	0.51 (0.45, 0.57)	1.00 (0.72, 1.40)	0.50 (0.44, 0.55)	0.89 (0.64, 1.25)	0.37 (0.32, 0.43)	0.84 (0.60, 1.18)	0.38 (0.33, 0.44)	0.75 (0.54, 1.04)
**Mental health**								
Excellent, very good, good	0.53 (0.50, 0.57)	Ref	0.54 (0.50, 0.57)	Ref	0.42 (0.38, 0.45)	Ref	0.45 (0.41, 0.48)	Ref

© 2026 Tesfaye L et al. *JAMA Network Open*.

Characteristics	Make sure you understand		Explain things		Spend enough time		Help deal with uncertainty	
	PM (95% CI)	aOR (95% CI)	PM (95% CI)	aOR (95% CI)	PM (95% CI)	aOR (95% CI)	PM (95% CI)	aOR (95% CI)
Fair, poor	0.45 (0.40, 0.51)	**0.68 (0.49, 0.93)**	0.45 (0.40, 0.51)	**0.66 (0.48, 0.91)**	0.35 (0.30, 0.40)	**0.71 (0.52, 0.98)**	0.37 (0.32, 0.43)	**0.71 (0.52, 0.97)**
**Has a primary care clinician**								
Yes	0.55 (0.51, 0.59)	Ref	0.55 (0.51, 0.59)	Ref	0.44 (0.40, 0.48)	Ref	0.45 (0.41, 0.49)	Ref
No	0.46 (0.42, 0.50)	**0.65 (0.49, 0.85)**	0.47 (0.42, 0.51)	**0.66 (0.50, 0.88)**	0.35 (0.31, 0.39)	**0.64 (0.48, 0.85)**	0.39 (0.35, 0.43)	**0.74 (0.56, 0.98)**
**Presence of underlying clinical conditions**								
Yes	0.52 (0.47, 0.57)	1.11 (0.81, 1.51)	0.57 (0.52, 0.62)	**1.50 (1.10, 2.06)**	0.42 (0.37, 0.47)	1.18 (0.86, 1.62)	0.42 (0.38, 0.48)	0.99 (0.73, 1.35)
No	0.50 (0.46, 0.54)	Ref	0.48 (0.44, 0.52)	Ref	0.38 (0.35, 0.42)	Ref	0.43 (0.39, 0.46)	Ref
**Past year in-person visit(s) with healthcare clinician**								
0 times	0.53 (0.43, 0.63)	Ref	0.51 (0.42, 0.61)	Ref	0.36 (0.26, 0.46)	Ref	0.41 (0.31, 0.52)	Ref
1 or more times	0.51 (0.48, 0.54)	0.90 (0.54, 1.47)	0.51 (0.48, 0.54)	0.99 (0.61, 1.61)	0.40 (0.37, 0.43)	1.25 (0.74, 2.10)	0.43 (0.40, 0.46)	1.07 (0.65, 1.78)
**History of COVID-19 infection**								
Yes	0.53 (0.48, 0.57)	1.11 (0.84, 1.46)	0.52 (0.47, 0.57)	1.04 (0.79, 1.38)	0.41 (0.36, 0.46)	1.07 (0.81, 1.41)	0.44 (0.39, 0.49)	1.11 (0.84, 1.45)
No	0.50 (0.47, 0.54)	Ref	0.51 (0.48, 0.54)	Ref	0.39 (0.36, 0.43)	Ref	0.42 (0.38, 0.45)	Ref
**Body mass index**								
Healthy (18.5 to <25)	0.48 (0.42, 0.53)	Ref	0.48 (0.43, 0.54)	Ref	0.40 (0.35, 0.46)	Ref	0.40 (0.34, 0.45)	Ref
Unhealthy (<18.5 or ≥25)	0.52 (0.49, 0.56)	1.24 (0.92, 1.66)	0.52 (0.49, 0.56)	1.22 (0.90, 1.65)	0.40 (0.36, 0.43)	0.97 (0.72, 1.31)	0.44 (0.40, 0.47)	1.20 (0.89, 1.61)
**Physical activity**								
Sufficient (≥150 minutes per week)	0.51 (0.48, 0.54)	Ref	0.50 (0.46, 0.53)	Ref	0.38 (0.35, 0.41)	Ref	0.40 (0.37, 0.44)	Ref

© 2026 Tesfaye L et al. *JAMA Network Open*.

Characteristics	Make sure you understand		Explain things		Spend enough time		Help deal with uncertainty	
	PM (95% CI)	aOR (95% CI)	PM (95% CI)	aOR (95% CI)	PM (95% CI)	aOR (95% CI)	PM (95% CI)	aOR (95% CI)
Insufficient (<150 minutes per week)	0.51 (0.45, 0.56)	0.98 (0.73, 1.32)	0.55 (0.50, 0.60)	1.28 (0.95, 1.74)	0.44 (0.39, 0.50)	1.34 (0.99, 1.82)	0.48 (0.42, 0.53)	**1.40 (1.04, 1.89)**
**Past month cigarette smoking**								
Yes	0.46 (0.41, 0.51)	**0.65 (0.47, 0.89)**	0.47 (0.42, 0.52)	**0.71 (0.52, 0.98)**	0.40 (0.36, 0.45)	1.04 (0.75, 1.43)	0.38 (0.33, 0.43)	**0.68 (0.50, 0.94)**
No	0.55 (0.51, 0.59)	Ref	0.54 (0.50, 0.58)	Ref	0.39 (0.36, 0.43)	Ref	0.46 (0.42, 0.50)	Ref
**Past month e-cigarette use**								
Yes	0.49 (0.43, 0.55)	0.88 (0.61, 1.26)	0.46 (0.40, 0.53)	0.73 (0.51, 1.05)	0.38 (0.32, 0.45)	0.90 (0.62, 1.30)	0.44 (0.38, 0.51)	1.09 (0.76, 1.57)
No	0.52 (0.48, 0.55)	Ref	0.53 (0.49, 0.56)	Ref	0.40 (0.37, 0.44)	Ref	0.42 (0.39, 0.45)	Ref
**Alcohol misuse**								
Yes	0.49 (0.39, 0.59)	0.89 (0.54, 1.44)	0.54 (0.43, 0.64)	1.09 (0.65, 1.84)	0.33 (0.24, 0.44)	0.70 (0.40, 1.20)	0.41 (0.31, 0.52)	0.94 (0.56, 1.59)
No	0.52 (0.49, 0.55)	Ref	0.52 (0.49, 0.55)	Ref	0.40 (0.37, 0.43)	Ref	0.43 (0.40, 0.46)	Ref
Not applicable (<21 years of age)	0.33 (0.20, 0.48)	**0.40 (0.19, 0.84)**	0.37 (0.23, 0.52)	0.48 (0.22, 1.03)	0.40 (0.28, 0.54)	1.00 (0.51, 1.98)	0.41 (0.27, 0.57)	0.93 (0.45, 1.94)
**Past month marijuana or cannabis use**								
Yes	0.47 (0.41, 0.52)	0.74 (0.54, 1.02)	0.47 (0.42, 0.52)	0.73 (0.53, 1.02)	0.34 (0.29, 0.40)	**0.66 (0.47, 0.92)**	0.40 (0.35, 0.46)	0.85 (0.62, 1.17)
No	0.53 (0.49, 0.57)	Ref	0.53 (0.50, 0.57)	Ref	0.43 (0.39, 0.46)	Ref	0.44 (0.40, 0.47)	Ref
**Device access**								
Yes	0.51 (0.48, 0.54)	Ref	0.51 (0.48, 0.54)	Ref	0.40 (0.37, 0.43)	Ref	0.42 (0.40, 0.45)	Ref
No	0.62 (0.29, 0.87)	1.70 (0.33, 8.62)	0.69 (0.36, 0.89)	2.45 (0.49, 12.27)	0.45 (0.15, 0.79)	1.27 (0.21, 7.65)	0.47 (0.14, 0.82)	1.20 (0.17, 8.20)
**Internet access**								

© 2026 Tesfaye L et al. *JAMA Network Open*.

Characteristics	Make sure you understand		Explain things		Spend enough time		Help deal with uncertainty	
	PM (95% CI)	aOR (95% CI)	PM (95% CI)	aOR (95% CI)	PM (95% CI)	aOR (95% CI)	PM (95% CI)	aOR (95% CI)
Yes	0.51 (0.48, 0.54)	Ref	0.51 (0.48, 0.54)	Ref	0.40 (0.37, 0.43)	Ref	0.42 (0.40, 0.45)	Ref
No	0.48 (0.28, 0.69)	0.88 (0.32, 2.43)	0.53 (0.30, 0.74)	1.09 (0.34, 3.43)	0.30 (0.13, 0.54)	0.58 (0.17, 1.94)	0.50 (0.28, 0.71)	1.40 (0.49, 4.00)
**Digital health literacy**								
Using technology to process health information	0.51 (0.48, 0.55)	0.93 (0.58, 1.50)	0.53 (0.49, 0.56)	0.76 (0.47, 1.23)	0.41 (0.37, 0.45)	0.80 (0.48, 1.33)	0.45 (0.41, 0.49)	0.69 (0.41, 1.14)
	0.50 (0.46, 0.55)		0.49 (0.45, 0.54)		0.38 (0.34, 0.43)		0.40 (0.36, 0.44)	
Understanding of health concepts and language	0.49 (0.45, 0.53)	1.41 (0.89, 2.24)	0.45 (0.41, 0.50)	**2.51 (1.56, 4.04)**	0.36 (0.32, 0.40)	**1.83 (1.13, 2.97)**	0.39 (0.35, 0.44)	1.56 (0.98, 2.48)
	0.53 (0.49, 0.58)		0.58 (0.53, 0.62)		0.43 (0.39, 0.48)		0.45 (0.41, 0.50)	
Ability to actively engage with digital services	0.52 (0.48, 0.56)	0.83 (0.55, 1.24)	0.52 (0.48, 0.55)	0.95 (0.63, 1.43)	0.42 (0.38, 0.46)	0.69 (0.46, 1.04)	0.45 (0.41, 0.49)	0.67 (0.45, 1.01)
	0.50 (0.46, 0.54)		0.51 (0.47, 0.55)		0.38 (0.34, 0.41)		0.40 (0.36, 0.44)	
Feel safe and in control	0.48 (0.45, 0.52)	**1.39 (1.01, 1.90)**	0.48 (0.44, 0.52)	**1.52 (1.11, 2.09)**	0.36 (0.32, 0.40)	**1.58 (1.13, 2.21)**	0.39 (0.35, 0.43)	**1.55 (1.11, 2.15)**
	0.53 (0.49, 0.56)		0.54 (0.50, 0.57)		0.42 (0.39, 0.45)		0.45 (0.41, 0.48)	
Motivated to engage with digital services	0.51 (0.47, 0.55)	0.93 (0.55, 1.55)	0.51 (0.47, 0.55)	0.98 (0.58, 1.65)	0.41 (0.36, 0.45)	0.88 (0.51, 1.49)	0.41 (0.37, 0.45)	1.25 (0.74, 2.12)
	0.50 (0.46, 0.55)		0.51 (0.46, 0.56)		0.39 (0.35, 0.43)		0.44 (0.39, 0.49)	
Access to digital services that work	0.45 (0.40, 0.51)	**1.89 (1.16, 3.09)**	0.49 (0.44, 0.54)	1.34 (0.81, 2.22)	0.36 (0.31, 0.41)	1.52 (0.92, 2.53)	0.39 (0.34, 0.45)	1.42 (0.84, 2.41)
	0.55 (0.51, 0.59)		0.53 (0.49, 0.57)		0.42 (0.38, 0.46)		0.45 (0.40, 0.49)	
Digital services that suit individual needs	0.47 (0.42, 0.52)	1.41 (0.97, 2.05)	0.49 (0.44, 0.54)	1.23 (0.83, 1.81)	0.32 (0.27, 0.37)	**2.00 (1.34, 2.98)**	0.36 (0.31, 0.41)	**1.81 (1.21, 2.69)**
	0.53 (0.49, 0.57)		0.52 (0.49, 0.56)		0.43 (0.40, 0.47)		0.46 (0.42, 0.50)	

Abbreviations: PM= predicted marginals, aOR= adjusted odds ratio, CI= confidence interval, Ref= reference group, AIAN= American Indian or Alaska Native, NHPI= Native Hawaiian or Pacific Islander, AA= African American.
Logistic regression analysis modeled the probability of 1= always. Bolded cells represent significant results where the confidence interval does not include the null value, 1.

© 2026 Tesfaye L et al. *JAMA Network Open*.

Predicted marginals are shown for top age of the 4 age brackets as they appear in Table 1, whereas predicted marginals for digital health literacy are shown for 25*th* and 75*th* percentile of the domain score.

© 2026 Tesfaye L et al. *JAMA Network Open*.

eTable 12. Factors associated with optimal patient-centered communication among those who had 1 or more telehealth visits in the past year and resided in MHSVI least vulnerable counties, *n* = 1000, complete case

Characteristics	Give chance to ask questions		Give attention to feelings		Involve you in decisions	
	PM (95% CI)	aOR (95% CI)	PM (95% CI)	aOR (95% CI)	PM (95% CI)	aOR (95% CI)
**Age**						
18	0.43 (0.36, 0.51)	1.00 (0.99, 1.01)	0.39 (0.32, 0.47)	1.00 (0.99, 1.01)	0.39 (0.32, 0.46)	1.01 (1.00, 1.02)
30	0.45 (0.40, 0.50)		0.41 (0.36, 0.46)		0.42 (0.37, 0.47)	
45	0.48 (0.45, 0.51)		0.42 (0.39, 0.46)		0.46 (0.43, 0.50)	
60	0.51 (0.46, 0.55)		0.44 (0.40, 0.48)		0.51 (0.47, 0.55)	
**Sex**						
Female	0.52 (0.47, 0.56)	**1.38 (1.02, 1.86)**	0.46 (0.42, 0.50)	1.33 (0.99, 1.80)	0.52 (0.47, 0.56)	**1.51 (1.12, 2.04)**
Male	0.45 (0.41, 0.50)	Ref	0.40 (0.36, 0.44)	Ref	0.43 (0.39, 0.47)	Ref
**Sexual orientation**						
Heterosexual	0.48 (0.45, 0.52)	Ref	0.43 (0.40, 0.46)	Ref	0.47 (0.44, 0.51)	Ref
Gay, lesbian, bisexual	0.50 (0.41, 0.59)	1.08 (0.70, 1.67)	0.44 (0.36, 0.53)	1.07 (0.69, 1.67)	0.48 (0.40, 0.57)	1.05 (0.67, 1.65)
**Race and/or ethnicity**						
AIAN, Asian, NHPI	0.35(0.24, 0.48)	**0.51 (0.26, 0.99)**	0.30 (0.20, 0.43)	**0.48 (0.24, 0.95)**	0.34 (0.22, 0.48)	**0.48 (0.23, 0.99)**
Black or AA	0.49 (0.37, 0.60)	0.98 (0.55, 1.72)	0.39 (0.28, 0.52)	0.77 (0.42, 1.42)	0.51 (0.41, 0.62)	1.15 (0.67, 1.97)
Hispanic or Latino	0.53 (0.42, 0.63)	1.20 (0.71, 2.04)	0.38 (0.28, 0.48)	0.70 (0.40, 1.23)	0.43 (0.33, 0.53)	0.77 (0.45, 1.29)
White	0.49 (0.45, 0.52)	Ref	0.45 (0.41, 0.48)	Ref	0.48 (0.45, 0.52)	Ref
**Education**						
<High school	0.49 (0.40, 0.59)	0.97 (0.80, 1.19)	0.44 (0.35, 0.54)	0.97 (0.79, 1.19)	0.56 (0.46, 0.64)	0.82 (0.68, 1.01)
High school graduate	0.49 (0.43, 0.55)		0.44 (0.38, 0.49)		0.52 (0.46, 0.57)	
Vocational school, some college	0.49 (0.45, 0.52)		0.43 (0.40, 0.46)		0.48 (0.45, 0.51)	
College graduate or higher	0.48 (0.43, 0.53)		0.42 (0.38, 0.47)		0.44 (0.40, 0.49)	
**Income**						
<$20,000	0.50 (0.44, 0.57)	0.95 (0.82, 1.10)	0.45 (0.39, 0.52)	0.93 (0.80, 1.08)	0.50 (0.44, 0.57)	0.92 (0.79, 1.06)
$20,000 to $49,999	0.49 (0.45, 0.53)		0.44 (0.40, 0.48)		0.49 (0.45, 0.53)	
$50,000 to $74,999	0.48 (0.45, 0.51)		0.43 (0.39, 0.46)		0.47 (0.44, 0.50)	
≥$75,000	0.47 (0.43, 0.52)		0.41 (0.37, 0.46)		0.45 (0.41, 0.50)	
**English proficiency**						
Very well	0.50 (0.47, 0.53)	Ref	0.44 (0.41, 0.47)	Ref	0.48 (0.45, 0.52)	Ref

© 2026 Tesfaye L et al. *JAMA Network Open*.

Characteristics	Give chance to ask questions		Give attention to feelings		Involve you in decisions	
	PM (95% CI)	aOR (95% CI)	PM (95% CI)	aOR (95% CI)	PM (95% CI)	aOR (95% CI)
Well, not well, not at all	0.25 (0.15, 0.38)	**0.27 (0.13, 0.56)**	0.29 (0.19, 0.42)	**0.46 (0.23, 0.92)**	0.29 (0.19, 0.43)	**0.38 (0.18, 0.77)**
**Health insurance**						
Insured	0.48 (0.45, 0.52)	Ref	0.43 (0.40, 0.46)	Ref	0.47 (0.44, 0.50)	Ref
Uninsured	0.52 (0.36, 0.68)	1.20 (0.53, 2.69)	0.42 (0.25, 0.60)	0.94 (0.38, 2.29)	0.53 (0.35, 0.71)	1.35 (0.53, 3.43)
**General health**						
Excellent, very good, good	0.47 (0.44, 0.51)	Ref	0.43 (0.39, 0.47)	Ref	0.46 (0.42, 0.49)	Ref
Fair, poor	0.52 (0.46, 0.58)	1.25 (0.87, 1.80)	0.43 (0.36, 0.49)	0.99 (0.68, 1.43)	0.52 (0.46, 0.59)	1.37 (0.95, 1.99)
**Mental health**						
Excellent, very good, good	0.50 (0.47, 0.54)	Ref	0.44 (0.41, 0.48)	Ref	0.50 (0.46, 0.53)	Ref
Fair, poor	0.44 (0.38, 0.50)	0.72 (0.51, 1.03)	0.39 (0.33, 0.45)	0.76 (0.53, 1.08)	0.42 (0.36, 0.48)	**0.69 (0.48, 0.97)**
**Has a primary care clinician**						
Yes	0.52 (0.48, 0.56)	Ref	0.47 (0.43, 0.51)	Ref	0.52 (0.48, 0.56)	Ref
No	0.42 (0.37, 0.47)	**0.61 (0.45, 0.83)**	0.36 (0.32, 0.41)	**0.60 (0.43, 0.82)**	0.40 (0.35, 0.45)	**0.57 (0.41, 0.78)**
**Presence of underlying clinical conditions**						
Yes	0.50 (0.46, 0.55)	1.17 (0.86, 1.60)	0.44 (0.40, 0.49)	1.13 (0.82, 1.55)	0.47 (0.43, 0.52)	0.99 (0.72, 1.36)
No	0.47 (0.43, 0.51)	Ref	0.42 (0.37, 0.46)	Ref	0.47 (0.43, 0.52)	Ref
**Past year in-person visit(s) with healthcare clinician**						
0 times	0.40 (0.30, 0.50)	Ref	0.37 (0.27, 0.47)	Ref	0.41 (0.31, 0.51)	Ref
1 or more times	0.49 (0.46, 0.53)	1.58 (0.93, 2.66)	0.43 (0.40, 0.47)	1.39 (0.81, 2.35)	0.48 (0.45, 0.51)	1.41 (0.84, 2.37)
**History of COVID-19 infection**						
Yes	0.50 (0.44, 0.55)	1.07 (0.78, 1.46)	0.47 (0.42, 0.53)	1.35 (0.98, 1.85)	0.54 (0.49, 0.59)	**1.58 (1.15, 2.15)**
No	0.48 (0.44, 0.52)	Ref	0.41 (0.38, 0.45)	Ref	0.45 (0.41, 0.48)	Ref
**Body mass index**						
Healthy (18.5 to <25)	0.47 (0.42, 0.53)	Ref	0.43 (0.38, 0.49)	Ref	0.47 (0.41, 0.52)	Ref

© 2026 Tesfaye L et al. *JAMA Network Open*.

Characteristics	Give chance to ask questions		Give attention to feelings		Involve you in decisions	
	PM (95% CI)	aOR (95% CI)	PM (95% CI)	aOR (95% CI)	PM (95% CI)	aOR (95% CI)
Unhealthy (<18.5 or ≥25)	0.49 (0.45, 0.53)	1.08 (0.78, 1.48)	0.43 (0.39, 0.46)	0.97 (0.70, 1.34)	0.48 (0.44, 0.51)	1.06 (0.76, 1.46)
**Physical activity**						
Sufficient (≥150 minutes per week)	0.50 (0.46, 0.54)	Ref	0.42 (0.39, 0.46)	Ref	0.48 (0.44, 0.52)	Ref
Insufficient (<150 minutes per week)	0.46 (0.41, 0.51)	0.83 (0.61, 1.13)	0.44 (0.39, 0.49)	1.07 (0.78, 1.45)	0.46 (0.41, 0.51)	0.88 (0.64, 1.20)
**Past month cigarette smoking**						
Yes	0.45 (0.38, 0.52)	0.78 (0.53, 1.15)	0.44 (0.38, 0.51)	1.08 (0.73, 1.60)	0.49 (0.42, 0.55)	1.07 (0.72, 1.59)
No	0.50 (0.46, 0.54)	Ref	0.42 (0.39, 0.46)	Ref	0.47 (0.43, 0.51)	Ref
**Past month e-cigarette use**						
Yes	0.42 (0.34, 0.50)	0.67 (0.43, 1.06)	0.40 (0.33, 0.49)	0.85 (0.55, 1.33)	0.47 (0.39, 0.55)	0.96 (0.61, 1.50)
No	0.50 (0.46, 0.53)	Ref	0.43 (0.40, 0.47)	Ref	0.48 (0.44, 0.51)	Ref
**Alcohol misuse**						
Yes	0.44 (0.33, 0.56)	0.81 (0.45, 1.44)	0.37 (0.27, 0.49)	0.74 (0.41, 1.33)	0.51 (0.40, 0.62)	1.22 (0.68, 2.18)
No	0.49 (0.45, 0.52)	Ref	0.43 (0.40, 0.46)	Ref	0.47 (0.44, 0.50)	Ref
Not applicable (<21 years)	0.55 (0.37, 0.71)	1.35 (0.57, 3.22)	0.52 (0.34, 0.68)	1.51 (0.63, 3.58)	0.55 (0.37, 0.72)	1.48 (0.60, 3.65)
**Past month marijuana or cannabis use**						
Yes	0.47 (0.40, 0.53)	0.88 (0.61, 1.27)	0.41 (0.34, 0.47)	0.86 (0.59, 1.25)	0.41 (0.35, 0.48)	**0.66 (0.46, 0.96)**
No	0.49 (0.45, 0.53)	Ref	0.44 (0.40, 0.47)	Ref	0.49 (0.46, 0.53)	Ref
**Device access**						
Yes	0.49 (0.45, 0.52)	Ref	0.43 (0.40, 0.46)	Ref	0.47 (0.44, 0.51)	Ref
No	0.39 (0.00079, 0.99)	0.62 (0.00022, 1740.69)	0.47 (0.0039, 0.99)	1.23 (0.0017, 846.83)	0.40 (0.00020, 0.99)	0.69 (0.000035, 13599.71)
**Internet access**						
Yes	0.48 (0.45, 0.52)	Ref	0.43 (0.40, 0.46)	Ref	0.47 (0.44, 0.51)	Ref
No	0.53 (0.21, 0.83)	1.26 (0.22, 7.27)	0.37 (0.10, 0.73)	0.72 (0.10, 4.78)	0.45 (0.15, 0.79)	0.88 (0.13, 5.64)

© 2026 Tesfaye L et al. *JAMA Network Open*.

Characteristics	Give chance to ask questions		Give attention to feelings		Involve you in decisions	
	PM (95% CI)	aOR (95% CI)	PM (95% CI)	aOR (95% CI)	PM (95% CI)	aOR (95% CI)
**Digital health literacy**						
Using technology to process health information	0.49 (0.45, 0.53)<br>0.47 (0.42, 0.53)	0.87 (0.50, 1.49)	0.44 (0.40, 0.48)<br>0.41 (0.36, 0.46)	0.73 (0.40, 1.32)	0.49 (0.45, 0.53)<br>0.44 (0.39, 0.49)	0.65 (0.38, 1.11)
Understanding of health concepts and language	0.46 (0.42, 0.51)<br>0.51 (0.46, 0.56)	1.46 (0.88, 2.42)	0.40 (0.36, 0.45)<br>0.46 (0.41, 0.50)	1.52 (0.91, 2.56)	0.45 (0.41, 0.50)<br>0.50 (0.45, 0.54)	1.42 (0.87, 2.32)
Ability to actively engage with digital services	0.48 (0.45, 0.52)<br>0.49 (0.44, 0.53)	1.00 (0.64, 1.56)	0.43 (0.39, 0.47)<br>0.43 (0.38, 0.47)	0.98 (0.62, 1.53)	0.46 (0.42, 0.50)<br>0.50 (0.45, 0.54)	1.30 (0.84, 2.03)
Feel safe and in control	0.45 (0.41, 0.49)<br>0.52 (0.48, 0.55)	**1.61 (1.16, 2.25)**	0.39 (0.35, 0.43)<br>0.46 (0.42, 0.50)	**1.65 (1.18, 2.32)**	0.44 (0.40, 0.48)<br>0.50 (0.47, 0.54)	**1.62 (1.16, 2.24)**
Motivated to engage with digital services	0.47 (0.44, 0.51)<br>0.51 (0.45, 0.56)	1.29 (0.75, 2.21)	0.42 (0.38, 0.46)<br>0.45 (0.40, 0.51)	1.34 (0.75, 2.39)	0.46 (0.43, 0.50)<br>0.49 (0.44, 0.55)	1.25 (0.72, 2.15)
Access to digital services that work	0.44 (0.39, 0.49)<br>0.52 (0.48, 0.57)	**1.75 (1.05, 2.92)**	0.36 (0.30, 0.42)<br>0.48 (0.43, 0.53)	**2.35 (1.36, 4.06)**	0.41 (0.36, 0.47)<br>0.52 (0.48, 0.57)	**2.11 (1.24, 3.59)**
Digital services that suit individual needs	0.48 (0.43, 0.53)<br>0.49 (0.44, 0.54)	1.03 (0.65, 1.64)	0.42 (0.37, 0.47)<br>0.44 (0.39, 0.48)	1.12 (0.70, 1.79)	0.47 (0.42, 0.52)<br>0.48 (0.43, 0.53)	1.10 (0.68, 1.78)

© 2026 Tesfaye L et al. *JAMA Network Open*.

eTable 12. Factors associated with optimal patient-centered communication among those who had 1 or more telehealth visits in the past year and resided in MHSVI least vulnerable counties, *n* = 1000, complete case (continued)

Characteristics	Make sure you understand		Explain things		Spend enough time		Help deal with uncertainty	
	PM (95% CI)	aOR (95% CI)	PM (95% CI)	aOR (95% CI)	PM (95% CI)	aOR (95% CI)	PM (95% CI)	aOR (95% CI)
**Age**								
18	0.40 (0.33, 0.47)	**1.02 (1.01, 1.03)**	0.41 (0.34, 0.49)	1.02 (1.00, 1.03)	0.33 (0.27, 0.40)	1.01 (1.00, 1.02)	0.35 (0.28, 0.42)	1.00 (0.99, 1.01)
30	0.45 (0.40, 0.50)		0.46 (0.41, 0.51)		0.37 (0.32, 0.42)		0.37 (0.32, 0.42)	
45	0.52 (0.49, 0.55)		0.52 (0.49, 0.55)		0.41 (0.38, 0.44)		0.39 (0.36, 0.42)	
60	0.59 (0.55, 0.63)		0.58 (0.54, 0.62)		0.46 (0.42, 0.50)		0.42 (0.38, 0.46)	
**Sex**								
Female	0.58 (0.53, 0.62)	**1.50 (1.11, 2.03)**	0.57 (0.53, 0.61)	**1.41 (1.05, 1.91)**	0.44 (0.40, 0.49)	1.22 (0.90, 1.64)	0.41 (0.37, 0.45)	1.12 (0.82, 1.52)
Male	0.49 (0.45, 0.53)	Ref	0.50 (0.46, 0.54)	Ref	0.40 (0.36, 0.45)	Ref	0.39 (0.34, 0.43)	Ref
**Sexual orientation**								
Heterosexual	0.54 (0.50, 0.57)	Ref	0.53 (0.50, 0.57)	Ref	0.41 (0.38, 0.45)	Ref	0.40 (0.37, 0.43)	Ref
Gay, lesbian, bisexual	0.50 (0.42, 0.59)	0.84 (0.54, 1.33)	0.55 (0.46, 0.63)	1.06 (0.68, 1.66)	0.50 (0.41, 0.58)	1.48 (0.95, 2.30)	0.42 (0.33, 0.51)	1.11 (0.70, 1.75)
**Race and/or ethnicity**								
AIAN, Asian, NHPI	0.42 (0.30, 0.55)	0.57 (0.30, 1.10)	0.39 (0.28, 0.52)	**0.49 (0.25, 0.94)**	0.24 (0.14, 0.37)	**0.33 (0.16, 0.70)**	0.23 (0.14, 0.36)	**0.38 (0.19, 0.78)**
Black or AA	0.64 (0.53, 0.74)	1.69 (0.96, 2.99)	0.64 (0.52, 0.75)	1.66 (0.89, 3.10)	0.37 (0.27, 0.49)	0.71 (0.39, 1.27)	0.44 (0.33, 0.56)	1.18 (0.65, 2.12)
Hispanic or Latino	0.52 (0.42, 0.62)	0.93 (0.56, 1.57)	0.51 (0.41, 0.61)	0.86 (0.51, 1.45)	0.41 (0.31, 0.52)	0.86 (0.51, 1.46)	0.36 (0.27, 0.47)	0.80 (0.46, 1.37)
White	0.53 (0.50, 0.57)	Ref	0.54 (0.50, 0.57)	Ref	0.44 (0.41, 0.48)	Ref	0.41 (0.38, 0.44)	Ref
**Education**								
<High school	0.64 (0.55, 0.72)	**0.78 (0.64, 0.95)**	0.62 (0.53, 0.70)	**0.81 (0.67, 0.99)**	0.49 (0.40, 0.59)	0.85 (0.69, 1.04)	0.47 (0.38, 0.57)	0.84 (0.69, 1.04)

© 2026 Tesfaye L et al. *JAMA Network Open.*

Characteristics	Make sure you understand		Explain things		Spend enough time		Help deal with uncertainty	
	PM (95% CI)	aOR (95% CI)	PM (95% CI)	aOR (95% CI)	PM (95% CI)	aOR (95% CI)	PM (95% CI)	aOR (95% CI)
High school graduate	0.59 (0.54, 0.64)		0.58 (0.53, 0.63)		0.46 (0.40, 0.52)		0.44 (0.38, 0.49)	
Vocational school, some college	0.54 (0.51, 0.57)		0.54 (0.51, 0.57)		0.43 (0.40, 0.46)		0.40 (0.37, 0.43)	
College graduate or higher	0.49 (0.44, 0.54)		0.50 (0.45, 0.55)		0.39 (0.35, 0.44)		0.37 (0.33, 0.42)	
**Income**								
<$20,000	0.55 (0.49, 0.61)	0.95 (0.82, 1.10)	0.54 (0.48, 0.60)	0.98 (0.84, 1.13)	0.43 (0.37, 0.50)	0.97 (0.83, 1.12)	0.42 (0.36, 0.49)	0.94 (0.80, 1.09)
$20,000 to $49,999	0.54 (0.50, 0.58)		0.54 (0.50, 0.58)		0.43 (0.39, 0.47)		0.41 (0.37, 0.45)	
$50,000 to $74,999	0.53 (0.50, 0.56)		0.54 (0.50, 0.57)		0.42 (0.39, 0.45)		0.40 (0.37, 0.43)	
≥$75,000	0.52 (0.47, 0.57)		0.53 (0.48, 0.58)		0.41 (0.37, 0.46)		0.38 (0.34, 0.43)	
**English proficiency**								
Very well	0.54 (0.51, 0.57)	Ref	0.55 (0.52, 0.58)	Ref	0.43 (0.40, 0.46)	Ref	0.41 (0.38, 0.44)	Ref
Well, not well, not at all	0.37 (0.25, 0.51)	**0.44 (0.22, 0.86)**	0.28 (0.17, 0.43)	**0.26 (0.12, 0.55)**	0.31 (0.20, 0.45)	0.55 (0.27, 1.11)	0.24 (0.14, 0.37)	**0.39 (0.18, 0.84)**
**Health insurance**								
Insured	0.53 (0.50, 0.56)	Ref	0.54 (0.50, 0.57)	Ref	0.42 (0.39, 0.45)	Ref	0.40 (0.37, 0.43)	Ref
Uninsured	0.59 (0.40, 0.76)	1.34 (0.52, 3.47)	0.55 (0.37, 0.71)	1.05 (0.43, 2.53)	0.38 (0.21, 0.58)	0.80 (0.30, 2.13)	0.32 (0.17, 0.52)	0.67 (0.25, 1.79)
**General health**								
Excellent, very good, good	0.52 (0.48, 0.55)	Ref	0.53 (0.49, 0.56)	Ref	0.41 (0.37, 0.45)	Ref	0.39 (0.36, 0.43)	Ref
Fair, poor	0.58 (0.52, 0.65)	1.39 (0.96, 2.02)	0.57 (0.50, 0.63)	1.21 (0.84, 1.76)	0.46 (0.39, 0.53)	1.27 (0.87, 1.86)	0.42 (0.35, 0.48)	1.11 (0.77, 1.61)
**Mental health**								
Excellent, very good, good	0.56 (0.52, 0.60)	Ref	0.55 (0.51, 0.59)	Ref	0.46 (0.42, 0.49)	Ref	0.43 (0.39, 0.47)	Ref

© 2026 Tesfaye L et al. *JAMA Network Open*.

Characteristics	Make sure you understand		Explain things		Spend enough time		Help deal with uncertainty	
	PM (95% CI)	aOR (95% CI)	PM (95% CI)	aOR (95% CI)	PM (95% CI)	aOR (95% CI)	PM (95% CI)	aOR (95% CI)
Fair, poor	0.48 (0.42, 0.54)	**0.67 (0.47, 0.95)**	0.50 (0.44, 0.56)	0.79 (0.55, 1.13)	0.34 (0.28, 0.40)	**0.56 (0.39, 0.80)**	0.32 (0.27, 0.38)	**0.58 (0.41, 0.83)**
**Has a primary care clinician**								
Yes	0.59 (0.55, 0.63)	Ref	0.57 (0.53, 0.61)	Ref	0.45 (0.41, 0.49)	Ref	0.43 (0.39, 0.47)	Ref
No	0.44 (0.39, 0.49)	**0.50 (0.37, 0.67)**	0.48 (0.43, 0.53)	**0.65 (0.48, 0.89)**	0.37 (0.32, 0.42)	**0.68 (0.49, 0.92)**	0.34 (0.30, 0.39)	**0.64 (0.46, 0.87)**
**Presence of underlying clinical conditions**								
Yes	0.53 (0.48, 0.58)	0.95 (0.70, 1.29)	0.53 (0.49, 0.58)	0.99 (0.72, 1.35)	0.43 (0.39, 0.48)	1.11 (0.81, 1.52)	0.42 (0.37, 0.46)	1.16 (0.84, 1.60)
No	0.54 (0.50, 0.58)	Ref	0.54 (0.49, 0.58)	Ref	0.41 (0.37, 0.46)	Ref	0.38 (0.34, 0.43)	Ref
**Past year in-person visit(s) with healthcare clinician**								
0 times	0.49 (0.39, 0.59)	Ref	0.49 (0.40, 0.59)	Ref	0.42 (0.32, 0.53)	Ref	0.37 (0.28, 0.47)	Ref
1 or more times	0.54 (0.51, 0.57)	1.24 (0.75, 2.06)	0.54 (0.51, 0.57)	1.25 (0.76, 2.07)	0.42 (0.39, 0.45)	0.99 (0.59, 1.66)	0.40 (0.37, 0.43)	1.18 (0.69, 2.00)
**History of COVID-19 infection**								
Yes	0.56 (0.51, 0.62)	1.20 (0.88, 1.65)	0.57 (0.51, 0.62)	1.25 (0.92, 1.70)	0.47 (0.41, 0.52)	1.36 (1.00, 1.86)	0.44 (0.39, 0.50)	1.34 (0.98, 1.84)
No	0.52 (0.49, 0.56)	Ref	0.52 (0.49, 0.56)	Ref	0.40 (0.37, 0.44)	Ref	0.38 (0.35, 0.42)	Ref
**Body mass index**								
Healthy (18.5 to <25)	0.54 (0.48, 0.59)	Ref	0.55 (0.50, 0.60)	Ref	0.46 (0.40, 0.51)	Ref	0.42 (0.36, 0.47)	Ref
Unhealthy (<18.5 or ≥25)	0.53 (0.50, 0.57)	0.96 (0.70, 1.31)	0.53 (0.49, 0.57)	0.90 (0.65, 1.23)	0.41 (0.37, 0.44)	0.79 (0.57, 1.09)	0.39 (0.36, 0.43)	0.88 (0.63, 1.24)
**Physical activity**								

© 2026 Tesfaye L et al. *JAMA Network Open*.

Characteristics	Make sure you understand		Explain things		Spend enough time		Help deal with uncertainty	
	PM (95% CI)	aOR (95% CI)	PM (95% CI)	aOR (95% CI)	PM (95% CI)	aOR (95% CI)	PM (95% CI)	aOR (95% CI)
Sufficient (≥150 minutes per week)	0.54 (0.50, 0.58)	Ref	0.53 (0.49, 0.57)	Ref	0.43 (0.39, 0.47)	Ref	0.41 (0.37, 0.45)	Ref
Insufficient (<150 minutes per week)	0.52 (0.47, 0.57)	0.88 (0.65, 1.20)	0.54 (0.49, 0.59)	1.05 (0.77, 1.43)	0.41 (0.36, 0.47)	0.94 (0.69, 1.29)	0.38 (0.33, 0.43)	0.86 (0.62, 1.18)
**Past month cigarette smoking**								
Yes	0.51 (0.45, 0.58)	0.88 (0.60, 1.28)	0.53 (0.46, 0.60)	0.96 (0.66, 1.41)	0.41 (0.35, 0.48)	0.93 (0.63, 1.37)	0.41 (0.35, 0.48)	1.09 (0.74, 1.61)
No	0.54 (0.50, 0.58)	Ref	0.54 (0.50, 0.57)	Ref	0.43 (0.39, 0.46)	Ref	0.39 (0.36, 0.43)	Ref
**Past month e-cigarette use**								
Yes	0.51 (0.42, 0.59)	0.85 (0.55, 1.30)	0.54 (0.46, 0.62)	1.05 (0.67, 1.63)	0.43 (0.35, 0.51)	1.03 (0.65, 1.63)	0.42 (0.34, 0.51)	1.14 (0.73, 1.79)
No	0.54 (0.50, 0.57)	Ref	0.53 (0.50, 0.57)	Ref	0.42 (0.39, 0.46)	Ref	0.39 (0.36, 0.43)	Ref
**Alcohol misuse**								
Yes	0.54 (0.43, 0.65)	1.02 (0.58, 1.78)	0.51 (0.40, 0.62)	0.87 (0.51, 1.50)	0.45 (0.34, 0.56)	1.14 (0.65, 2.02)	0.34 (0.24, 0.45)	0.71 (0.39, 1.29)
No	0.53 (0.50, 0.57)	Ref	0.54 (0.51, 0.57)	Ref	0.42 (0.39, 0.45)	Ref	0.40 (0.37, 0.43)	Ref
Not applicable (<21 years of age)	0.55 (0.37, 0.72)	1.08 (0.44, 2.64)	0.53 (0.35, 0.71)	0.97 (0.38, 2.44)	0.43 (0.26, 0.61)	1.03 (0.41, 2.58)	0.49 (0.30, 0.68)	1.55 (0.57, 4.19)
**Past month marijuana or cannabis use**								
Yes	0.53 (0.46, 0.59)	0.95 (0.66, 1.37)	0.48 (0.42, 0.55)	0.72 (0.50, 1.03)	0.39 (0.33, 0.46)	0.82 (0.56, 1.21)	0.36 (0.30, 0.42)	0.75 (0.52, 1.09)
No	0.54 (0.50, 0.57)	Ref	0.55 (0.52, 0.59)	Ref	0.43 (0.40, 0.47)	Ref	0.41 (0.38, 0.45)	Ref
**Device access**								
Yes	0.53 (0.50, 0.56)	Ref	0.54 (0.51, 0.57)	Ref	0.42 (0.39, 0.45)	Ref	0.40 (0.37, 0.43)	Ref
No	0.32 (0.000005, 0.99)	0.34 (0.00, --)[a]	0.30 (0.000003, 0.99)	0.30 (0.00, --)[a]	0.37 (0.000292, 0.99)	0.79 (0.000093, 6717.09)	0.36 (0.000057, 0.99)	0.82 (0.000014, 49778.44)

© 2026 Tesfaye L et al. *JAMA Network Open*.

Characteristics	Make sure you understand		Explain things		Spend enough time		Help deal with uncertainty	
	PM (95% CI)	aOR (95% CI)	PM (95% CI)	aOR (95% CI)	PM (95% CI)	aOR (95% CI)	PM (95% CI)	aOR (95% CI)
**Internet access**								
Yes	0.53 (0.50, 0.56)	Ref	0.54 (0.50, 0.57)	Ref	0.42 (0.39, 0.45)	Ref	0.40 (0.37, 0.43)	Ref
No	0.63 (0.22, 0.91)	1.64 (0.18, 14.36)	0.65 (0.34, 0.87)	1.76 (0.37, 8.35)	0.57 (0.28, 0.82)	2.08 (0.47, 9.02)	0.47 (0.20, 0.76)	1.44 (0.31, 6.65)
**Digital health literacy**								
Using technology to process health information	0.55 (0.52, 0.59)	0.63 (0.37, 1.07)	0.57 (0.54, 0.61)	**0.33 (0.19, 0.59)**	0.44 (0.40, 0.48)	0.66 (0.38, 1.15)	0.43 (0.39, 0.47)	**0.52 (0.29, 0.93)**
	0.50 (0.45, 0.55)		0.45 (0.40, 0.50)		0.39 (0.35, 0.44)		0.36 (0.31, 0.40)	
Understanding of health concepts and language	0.50 (0.45, 0.54)	**1.89 (1.15, 3.12)**	0.47 (0.43, 0.52)	**2.92 (1.73, 4.93)**	0.40 (0.36, 0.45)	1.41 (0.83, 2.40)	0.36 (0.31, 0.40)	**1.97 (1.17, 3.33)**
	0.58 (0.53, 0.63)		0.61 (0.57, 0.66)		0.44 (0.40, 0.49)		0.44 (0.40, 0.49)	
Ability to actively engage with digital services	0.52 (0.49, 0.56)	1.29 (0.84, 2.00)	0.51 (0.48, 0.55)	**1.65 (1.04, 2.62)**	0.42 (0.38, 0.45)	1.12 (0.71, 1.77)	0.40 (0.36, 0.44)	0.94 (0.59, 1.50)
	0.56 (0.51, 0.60)		0.58 (0.53, 0.62)		0.43 (0.38, 0.48)		0.40 (0.35, 0.44)	
Feel safe and in control	0.51 (0.47, 0.55)	**1.44 (1.05, 1.98)**	0.53 (0.49, 0.57)	1.09 (0.78, 1.52)	0.37 (0.33, 0.41)	**2.08 (1.44, 3.00)**	0.36 (0.33, 0.40)	**1.63 (1.15, 2.31)**
	0.56 (0.52, 0.60)		0.54 (0.50, 0.58)		0.47 (0.43, 0.50)		0.43 (0.39, 0.46)	
Motivated to engage with digital services	0.53 (0.49, 0.56)	1.19 (0.71, 1.99)	0.51 (0.48, 0.55)	1.71 (1.00, 2.91)	0.41 (0.37, 0.45)	1.26 (0.72, 2.19)	0.38 (0.34, 0.42)	1.62 (0.92, 2.83)
	0.55 (0.50, 0.60)		0.58 (0.53, 0.64)		0.44 (0.39, 0.50)		0.44 (0.38, 0.49)	
Access to digital services that work	0.52 (0.47, 0.57)	1.19 (0.71, 1.99)	0.51 (0.46, 0.56)	1.42 (0.85, 2.37)	0.39 (0.33, 0.45)	1.49 (0.84, 2.62)	0.33 (0.28, 0.39)	**2.23 (1.26, 3.93)**
	0.55 (0.50, 0.59)		0.56 (0.51, 0.61)		0.45 (0.40, 0.49)		0.45 (0.40, 0.49)	

© 2026 Tesfaye L et al. *JAMA Network Open*.

Characteristics	Make sure you understand		Explain things		Spend enough time		Help deal with uncertainty	
	PM (95% CI)	aOR (95% CI)	PM (95% CI)	aOR (95% CI)	PM (95% CI)	aOR (95% CI)	PM (95% CI)	aOR (95% CI)
Digital services that suit individual needs	0.51 (0.46, 0.55)	1.41 (0.90, 2.22)	0.51 (0.47, 0.56)	1.36 (0.84, 2.19)	0.41 (0.36, 0.46)	1.17 (0.73, 1.90)	0.40 (0.35, 0.45)	0.97 (0.61, 1.56)
	0.56 (0.51, 0.61)		0.56 (0.51, 0.61)		0.43 (0.39, 0.48)		0.40 (0.35, 0.44)	

Abbreviations: PM= predicted marginals, aOR= adjusted odds ratio, CI= confidence interval, Ref= reference group, AIAN= American Indian or Alaska Native, NHPI= Native Hawaiian or Pacific Islander, AA= African American.
Logistic regression analysis modeled the probability of 1= always. Bolded cells represent significant results where the confidence interval does not include the null value, 1.
Predicted marginals are shown for top age of the 4 age brackets as they appear in Table 1, whereas predicted marginals for digital health literacy are shown for 25*th* and 75*th* percentile of the domain score.
[a] aOR was undefined.

© 2026 Tesfaye L et al. *JAMA Network Open*.

eFigure. Adjusted odds ratios for participants' reporting of optimal patient-centered communication among those who had 1 or more telehealth visits in the past year, *N* = 2754.

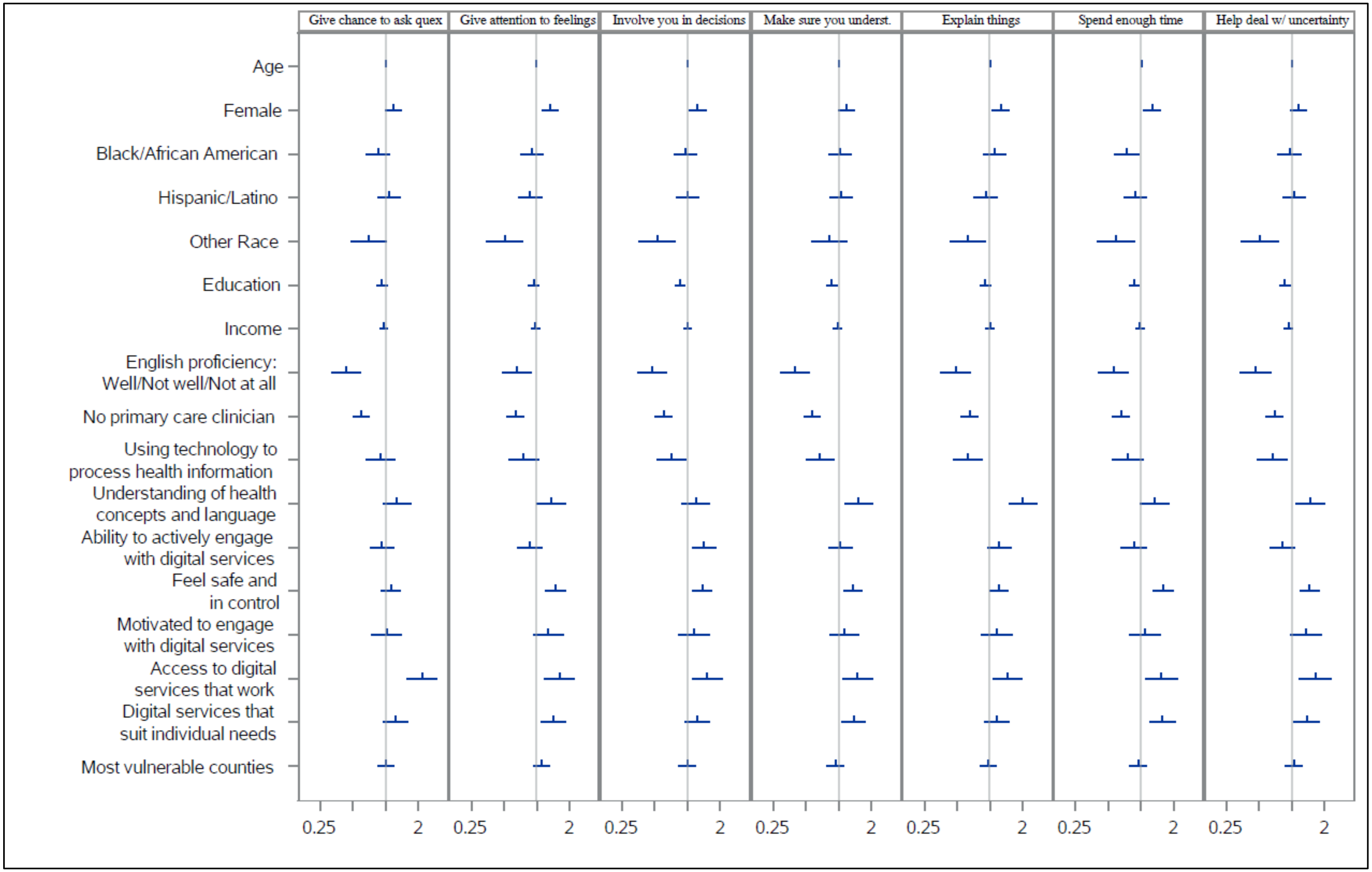

© 2026 Tesfaye L et al. *JAMA Network Open*.
