# Supplementary material for: Patient-Centered Communication in Telehealth Settings
Source: JAMA Netw Open. 2026 Jan 29;9(1):e2556291. doi: 10.1001/jamanetworkopen.2025.56291 (PMC12856684; doi:10.1001/jamanetworkopen.2025.56291)
Supplement: Supplement 2. — Data Sharing Statemen [file jamanetwopen-e2556291-s002.pdf]

Data Sharing Statement

Tesfaye. Patient-Centered Communication in Telehealth Settings. *JAMA Netw Open*.
Published January 29, 2026. doi:10.1001/jamanetworkopen.2025.56291

Data
Data available: Yes
Data types: Deidentified participant data
How to access data: Data are available upon request from the corresponding author.
When available: With publication

Supporting Documents
Document types: None

Additional Information
Who can access the data: Researchers whose proposed use of the data has been approved.
Types of analyses: For a specified purpose.
Mechanisms of data availability: With a signed data access agreement.
